# Prevalent and sex-biased breathing patterns modify functional connectivity MRI in young adults

**DOI:** 10.1038/s41467-020-18974-9

**Published:** 2020-10-20

**Authors:** Charles J. Lynch, Benjamin M. Silver, Marc J. Dubin, Alex Martin, Henning U. Voss, Rebecca M. Jones, Jonathan D. Power

**Affiliations:** 1 Brain and Mind Research Institute, Weill Cornell Medicine, 1300 York Avenue, New York, NY 10065 USA; 2 Sackler Institute for Developmental Psychobiology, Department of Psychiatry, Weill Cornell Medicine, 1300 York Avenue, New York, NY 10065 USA; 3 Department of Psychiatry, Weill Cornell Medicine, 1300 York Avenue, New York, NY 10065 USA; 4 National Institute of Mental Health, 10 Center Dr., Bethesda, MD 20892 USA; 5 Department of Radiology, Weill Cornell Medicine, Citigroup Biomedical Imaging Center, 516 East 72nd Street, New York, NY 10021 USA

**Keywords:** Cognitive neuroscience, Respiration

## Abstract

Resting state functional connectivity magnetic resonance imaging (fMRI) is a tool for investigating human brain organization. Here we identify, visually and algorithmically, two prevalent influences on fMRI signals during 440 h of resting state scans in 440 healthy young adults, both caused by deviations from normal breathing which we term deep breaths and bursts. The two respiratory patterns have distinct influences on fMRI signals and signal covariance, distinct timescales, distinct cardiovascular correlates, and distinct tendencies to manifest by sex. Deep breaths are not sex-biased. Bursts, which are serial taperings of respiratory depth typically spanning minutes at a time, are more common in males. Bursts share features of chemoreflex-driven clinical breathing patterns that also occur primarily in males, with notable neurological, psychiatric, medical, and lifespan associations. These results identify common breathing patterns in healthy young adults with distinct influences on functional connectivity and an ability to differentially influence resting state fMRI studies.

## Abstract (web summary)

Functional connectivity measured from fMRI data is widely used in neuroscience. Here the authors report an association between two types of breathing signature and obtained BOLD data, and associated sex differences.

## Introduction

Functional magnetic resonance imaging (fMRI) scanning of subjects at rest has become a major neuroimaging paradigm, termed functional connectivity or resting state MRI^1^. In these scans, subjects lie quietly, often staring at a crosshair, for 5–15 min or more, performing no particular instructed task while fMRI data are acquired. Correlations in task-free fMRI signals are thought to reflect the functional relatedness of the tissues producing those signals, and the spatial topography of signal correlations has been leveraged to yield new and increasingly refined macro-scale maps of the human brain^2–4^. These scans have the potential to deliver diagnostic and prognostic information, and large studies are now underway that use resting-state fMRI scans as cornerstones of the datasets, e.g., the ABCD study scanning 10,000 children for biomarkers of developmental trajectories^5^.

Breathing modifies the concentration of carbon dioxide in arterial blood, which is a potent modulator of cerebral blood flow, and thus the fMRI signal^6,7^. When subjects breathe deeply or quickly (i.e., hyperpnea) they exhale more CO_2,_ arterial pCO_2_ drops_,_ cerebral blood flow decreases, and fMRI signals decrease; conversely, if breathing is shallow or slow (or stopped) (i.e., hypopnea or apnea), less CO_2_ is released, arterial pCO_2_ rises, cerebral blood flow increases, and fMRI signals increase. In this manner, breathing patterns can influence resting-state fMRI scans.

Breathing occurs in multiple forms. The basic respiratory rhythm is a cyclic rhythm termed eupnea, which moves a tidal volume of air into and out of the lungs in each breath. But a variety of deviations from eupnea exist, from the sighs exhibited by all humans that reinflate collapsed portions of the lung, to yawns of boredom or sleepiness, to more marked forms of disordered breathing, including cluster breathing, ataxic breathing, or periodic breathing (e.g., Hunter–Cheyne–Stokes), forms of respiration often associated with heart disease or neurological injury^6^. Beyond having different generative neural mechanisms^8^, different kinds of breathing may have distinct biophysical correlates and consequences for neuroimaging.

Little is known about the breathing characteristics of healthy young adults lying at rest in an MRI scanner, the kind of subject that forms the backbone of the functional connectivity literature, despite the potential for breathing to systematically influence fMRI signals. To address this issue, we jointly examined respiration and fMRI signals in a large, publicly available data set of healthy young adults with large amounts of scan time per subject, the Young Adult release of the Human Connectome Project (HCP). In this report, we describe effects seen in 440 h of scanning in 440 subjects (ages 22–36, mean 28.6, 228 males, 212 females). Such quantities of data stand in contrast to the prior fMRI literature on respiration, which usually involved small numbers of relatively short recordings^9–12^.

The sheer size of the Young Adult HCP data set provides an unprecedented window into the respiratory behavior of humans quietly resting in scanners. In these subjects, beyond eupnea, we find two prevalent patterns in respiration with distinct correlates in fMRI signals and distinct influences on functional connectivity. One pattern represents isolated deep breaths and is not sex-biased. The other, termed bursts, is sex-biased, and we link this pattern to sex-biased breathing patterns in the respiratory literature. Patterns were congruently recognized by human raters and an algorithmic scoring system. Collectively, these results demonstrate a prevalent and sex-biased form of breathing in healthy young adults with substantial influence on functional connectivity measures that resemble a form of breathing traditionally studied in older, medically ill patients. The clinical literature suggests that these breathing patterns will be influenced by sex hormones, by age, cardiovascular, neurological, and psychiatric illness, among other factors.

## Results

### Two distinct breathing patterns in subjects at rest

We begin by presenting individual instances of respiratory patterns, then group descriptions, then demographic differences in pattern prevalence, and then the spatiotemporal effects of the breathing patterns and their distinct consequences for fMRI signal covariance. We focus initially on visual presentations, for these were how we first recognized the patterns.

To detect influences of respiration on resting-state fMRI signals, we created and viewed plots of 1760 scans representing 440 h of scanning in 440 young adults. In addition to eupnea, we came to recognize two common patterns of respiration. One pattern was known to us, which we term a single deep breath, a lone breath considerably larger than the surrounding breaths. The other pattern was unfamiliar to us, and is undescribed in the neuroimaging literature to our knowledge; we call it a burst pattern.

**Fig. 1:**
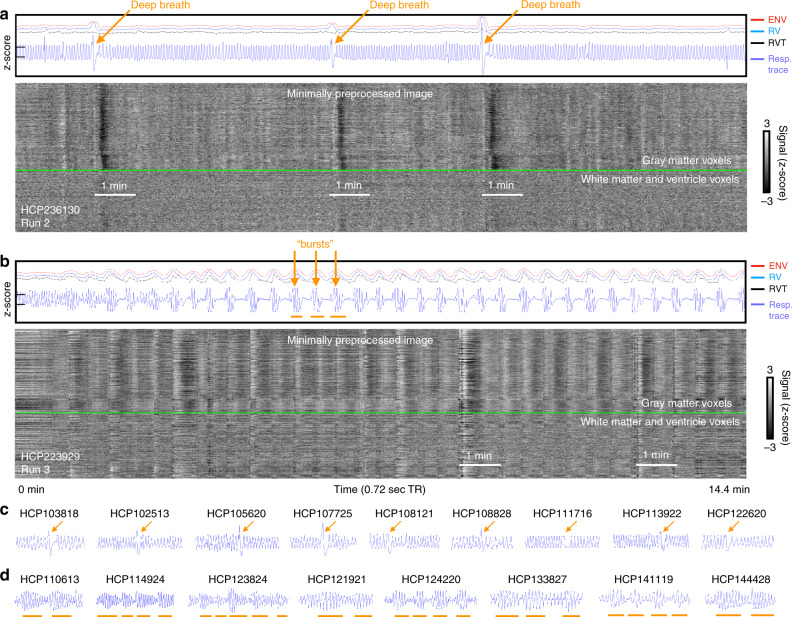
Gray plots of scans containing deep breaths and bursts. Gray plots containing **a** deep breaths and **b** bursts. Upper panels show the *z*-scored respiratory belt traces in blue (*y*-ticks at left are *z* = −1 and 1), and 3 commonly derived respiratory measures (ENV, RV, and RVT, with vertical offsets to enable non-overlapping visualization; scales are identical in all figures). In the grayscale heat maps, all in-brain fMRI signals are shown organized by anatomical compartment, with a green line separating gray matter from white matter and ventricle signals. In **a**, three deep breaths are indicated by arrows, with major decreases (vertical black bands) in fMRI signal following each of the breaths. In **b**, over two dozen bursts are present (arrows mark several), with accompanying modulations of fMRI signals. Supplementary Figs. 1 and 2 show comprehensive versions of each scan. Additional exemplars of deep breaths (**c**) and bursts (**d**) are shown at the bottom. The code to produce gray plots was published in ref. ^49^, and comprehensive gray plots of all 17,640 scans are shown in Supplementary Movie 1.

The two patterns are illustrated in Fig. 1. The fMRI scans are flattened into grayscale heat maps, with all in-brain voxels defining the *Y* axis and time on the *X* axis. Signals from gray matter voxels are above the bright green lines, and from white matter and ventricles below. The respiratory belt trace is shown in blue, and several deep breaths are marked by orange arrows in an otherwise eupneic scan in Fig. 1a. Three respiratory measures derived from the respiratory trace often used to model respiratory effects in the fMRI literature, are also shown (ENV, RV, and RVT, respectively, gauging the envelope of the belt trace, windowed variance in the trace, and the rate of air movement), often displaying abnormalities at these deep breaths. In the fMRI signals, throughout the gray matter, there are brief signal increases (vertical white bands) just after the deep breath is taken, followed by prominent signal decreases (vertical black bands). Figure 1b illustrates a burst respiratory pattern: a serial, rhythmic set of tapers in breathing depth (this example has apnea between bursts), with rhythmic correlates in fMRI signals. Fuller versions of these images, including fMRI signals before and after denoising, are shown in Supplementary Figs. 1 and 2, illustrating that signal effects are present both before and after FIX-ICA denoising and that the patterns are also linked to head motion and image quality measures. These scans were chosen for their stark examples of the breathing patterns, but there are many forms of deep breaths and bursts, illustrated in Fig. 1c, d and later figures. In something as straightforward as a single deep breath (e.g., Fig. 1c), a variety of waveforms are possible, including floor and ceiling effects and slippage of the respiratory belt at peak inspiration; readers wishing to gain skill in recognizing patterns are encouraged to consult Supplementary Note 1 and to view all 1760 gray plots in Supplementary Movie 1 (1.4 GB, download at https://osf.io/u35f8/).

**Fig. 2:**
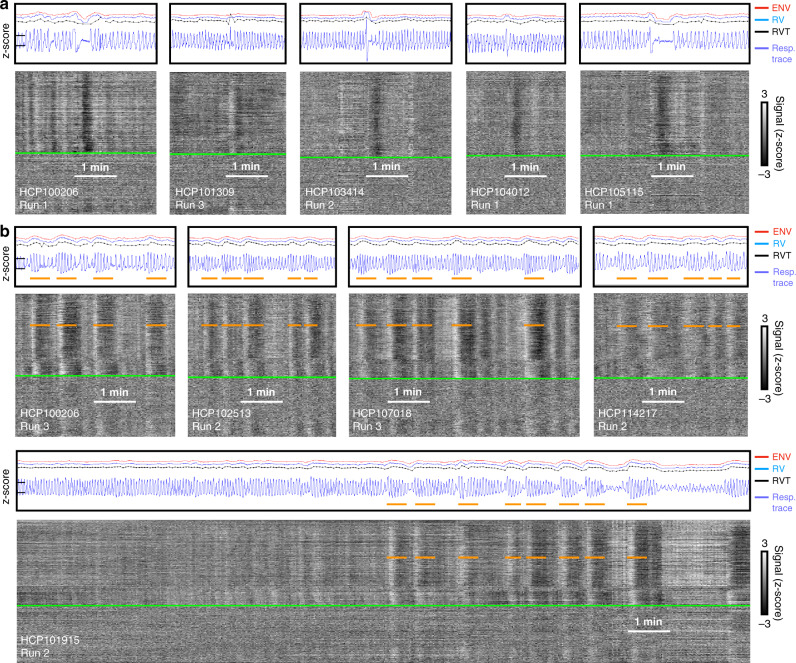
Examples of deep breaths and bursts. Plots are formatted as in Fig. 1. Note in deep breaths **a** the slowness of the breaths, the possibility of transient apnea afterward, the incongruence of the respiratory measures ENV, RV, and RVT, and the presence of fMRI signal decreases in each instance. Note in bursts **b** the repeated, serial modulation of breathing amplitude, the congruency of ENV, RV, and RVT, and the rhythmic correlates in fMRI signals.

To help convey the variety of respiratory waveforms denoting single deep breaths, five instances are shown in Fig. 2a (fuller images are shown in Supplementary Fig. 3, see also dozens of scans with deep breaths marked in Supplementary Movie 2). These isolated, deep breaths are often accompanied by subsequent breathing pauses of variable duration (often just a few seconds but sometimes lasting 10 or 20 s; brief central apneas are known sequelae of deep breaths^13^). In each of these instances, after an initial delay, a black band in the gray plot reflects a pan-brain decrease in fMRI signals lasting until ~30 s after the breath, consistent with a cerebral blood flow decrease after a transient increase in ventilation.

To help convey the variety of respiratory waveforms denoting bursts, five instances are shown in Fig. 2b (fuller images are in Supplementary Fig. 4, see also dozens of scans with bursts marked in Supplementary Movie 3). In this pattern, a burst of deep breaths occurs which tapers into shallow breaths, often serially followed by additional bursts. These burst respiratory patterns differ from single deep breath patterns in several respects. First, whereas single deep breaths often occur in isolation, series of bursts often span several minutes at a time, with individual bursts often lasting ~30–50 s. Second, the burst patterns are usually quite evident in ENV, RV, and RVT traces, which all tend to concordantly display large wavelike modulations (in contrast, note the lack of concordance in some deep breaths of Fig. 2). Third, the typical fMRI signal response is an initial signal increase followed by a prolonged decrease (see orange lines marking brief white then longer black bands), with durations approximately matching those of the respiratory burst with an added lag of signal decrease. The subject at the bottom is shown for the entirety of a scan, illustrating how eupnea evolves into a burst pattern, with the emergence of fMRI signal correlates.

Each subject had four 14.4-min long scans, and it was plain that, within a subject, one scan could display normal tidal breathing, but a different scan could contain markedly different breathing, with accompanying differences in fMRI signals (see two examples in Supplementary Fig. 5). It was also plain that, within scans, different breathing patterns could dominate at different times (see two examples in Supplementary Fig. 6). Because deep breaths and bursts were both prevalent, and organized, we focused on these respiratory events (though other forms of more disorganized breathing exist, see an example in Supplementary Fig. 5, upper right).

### Properties of respiratory patterns

**Fig. 3:**
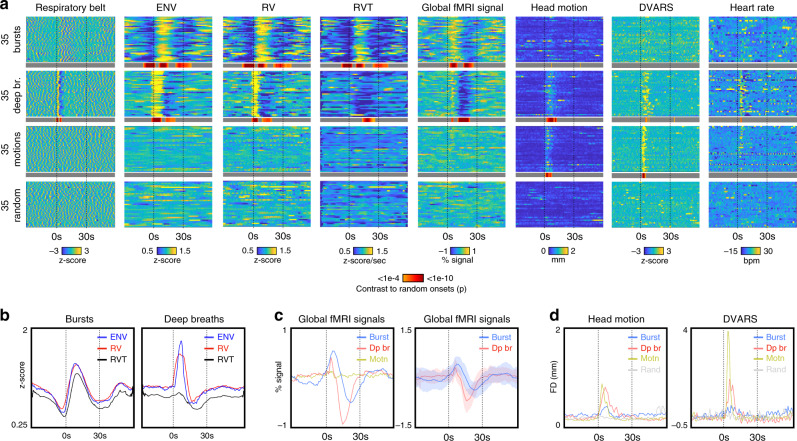
Properties of deep breaths and bursts. **a** Heat maps at the top illustrate 90-s segments surrounding visually identified events. In different subjects, 35 examples each of bursts, deep breaths, and isolated non-respiratory motions were identified from respiratory belt traces and motion traces. In the motion-exhibiting subjects, a random set of timepoints was also selected. Illustrated are respiratory belt traces, three respiratory measures (ENV, RV, and RVT), the global (gray matter average) fMRI signal, head motion (framewise displacement, following filtering, and 4-TR calculation as in ref. ^16^), and DVARS (*z*-scored). A gray/red heatmap represents statistically significant differences from the random events (two-sample *t*-test, two-tailed) beyond *p* < 0.001, illustrated on a logarithmic scale capped at *p* < 1e−10. The basis of bursts and deep breaths are apparent in the respiratory belt images. **b** Mean signals of ENV, RV, and RVT congruently mark bursts, but deep breaths display differences across the respiratory measures, with RVT having little positive deflection. **c** Mean global fMRI signals differ across patterns: deep breaths have brief signal increases and marked signal decreases with nadir near 15 s, and 30 s to resolution, on average. Bursts have more marked positive deflections, and slower timecourses on average, resolving near 40 s on average. Motion produces no global fMRI signal changes. Shade plots reflect mean and std. **d** Deep breaths display considerable motion and DVARS changes time-locked to event onsets; bursts have smaller time-locked modulations that do not achieve significance. Source data are provided as a source data file.

To more formally characterize deep breaths and bursts, the onsets of 35 bursts, 35 deep breaths, and 35 non-respiratory head motions were visually identified (Supplementary Data 1 lists onsets; Supplementary Fig. 7 illustrates 6 onsets of each kind in gray plots, see Supplementary Movies 2–4 for all onsets marked in gray plots). The non-respiratory motion onsets were identified to address the possibility that the fMRI signal changes during deep breaths or bursts related to head motion from breathing. A random onset in a random scan of the motion subjects was also set as a control condition. Relevant signals were extracted from 30 s prior to 60 s after onsets, illustrated in Fig. 3a, with statistical contrast to random onsets shown as a thin gray-red heatmap under the main heat maps (coloring only *t*-tests of *p* < 0.001). The basis of burst and deep breath identification is evident in the respiratory belt heat maps. Plots in Fig. 3b show mean values across certain events. Bursts show marked, congruent signatures in ENV, RV, and RVT (Fig. 3b). Deep breaths also have marked signatures, but they differ across measures, with RVT having no mean positive deflection (explained in ref. ^14^). Global fMRI signals differ across patterns, with deep breaths on average displaying a brief, steep signal increase then a marked signal trough with nadir near 15 s after onset, and resolution 30 s after onset. Bursts have slower trajectories on average, peaking later and higher, and exhibiting prolonged troughs with nadir over 20 s after onset, with a resolution around 40 s after onset. Individual event durations are naturally modulated by the depth of breathing, the presence and duration of apnea after deep breaths, and the duration of a burst taper. We selected a non-respiratory-motion group as a control because deep breaths display considerable motion at the onset of the breath, evident in the head motion and DVARS heat maps. However, neither the motion-displaying group nor the random group showed any global fMRI signal fluctuations, effectively ruling out motion as a cause of the patterned global signals. That motion did not produce global changes is consistent with the fact that multi-echo studies show that global fMRI signals are overwhelmingly T2* signals (compatible with respiration), not S0 artifacts caused by head motion^15^. Heart rate is routinely elevated for several seconds after deep breaths, whereas no average effect is noted for bursts. Because deep breaths very reliably elevate heart rates, we were surprised that deepened breathing in bursts did not produce much modulation; review of individual scans indicates that some subjects reliably display cardiac modulation by bursts, but others display no modulation (Supplementary Fig. 8). Other non-HCP resting-state fMRI datasets also exhibit bursts, and, in those datasets, as in the HCP data, there is not a reliable link between bursts and heart rate modulation (see Supplementary Note 2 and Supplementary Fig. 9).

### Group contrasts reveal a sex bias in burst patterns

To better understand why the respiratory patterns occur, we sought subject-level factors that scaled with these two respiratory patterns. The HCP data set has hundreds of behavioral, demographic, physiologic, and imaging measures for each subject. To mine such information, we needed either groups to contrast displaying different breathing patterns, or numeric indices of patterns (e.g., for regression). We pursued both paths simultaneously but prioritized the group contrast approach because we could carefully select and thus confidently characterize breathing patterns in the groups.

For exploratory purposes, we defined three groups with clearly different breathing patterns: subjects with unambiguous, marked bursts in most or all scans and few or no single deep breaths (the burst group), subjects with unambiguous, marked single deep breaths in most or all scans but few or no bursts (the deep breath group), and subjects whose scans displayed neither bursts nor single deep breaths (the clean group). Subjects were selected based on respiratory belt traces and signal heat maps alone, without knowledge of other properties of the subject. This procedure identified three groups of 21 subjects, all unrelated. Subject identity is restricted due to associations with psychiatric instrument scores and substance use in the groups (described below); investigators with HCP Restricted Access will find a Subject Key associated with this paper identifying the groups. Full details of the statistical contrasts of these groups are reported in Supplementary Note 3, Supplementary Fig. 10, and Supplementary Data 2. Instructions to access Subject Keys are in the “Methods” section.

The three groups were associated with far more HCP variables than expected by chance, including alcohol use (bursts), cigarette use (deep breaths), and strikingly included dozens of structural imaging variables that differed uniformly by group, distinguishing the burst group (thinner cortex) from other groups (smaller brains) (Supplementary Fig. 10). These findings were all subsumed, and nearly all explainable, by the following fact: males were 6/21 of the clean group, 5/21 of the deep breath group, and 14/21 of the burst group. The groups were formed without knowledge of the sex of participants, and it is very unlikely (joint probability *p* = 3.3e−5) that such unbalanced sex compositions would emerge three times in random group formation.

### Ratings of gray plots reveal a sex bias in burst patterns

In parallel with the group analyses, authors J.D.P. and C.J.L. independently rated 1596 scans (subjects 1–399) after training together on subjects 400–440, making binary decisions on the presence of deep breaths and bursts in each scan. Ratings were made purely in terms of gray plots without knowledge of any demographics, including sex. The group results above were discovered after 100 subjects had been rated (with good-to-excellent inter-rater reliability, Cohen’s kappas were 0.79 for bursts and 0.73 for deep breaths). Significant sex differences in bursts but not deep breaths were present within these first 100 subjects for both raters, were again separately present in the next 299 subjects rated for both raters, and were also present when subjects in the above-defined groups were excluded and/or when only one subject per family contributed. For simplicity, we report ratings of the entire 399-subject cohort.

**Fig. 4:**
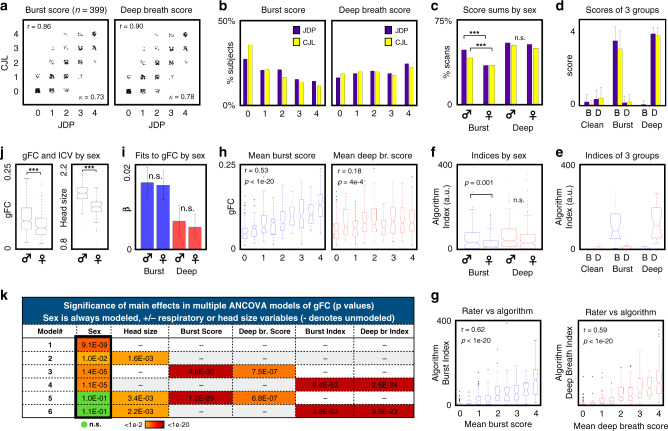
Rater scorings and algorithmic indices detect sex effects and global functional connectivity influences. **a** Plots of total scans (of 4) with bursts and deep breaths for both raters, with score correlations and Cohen’s kappas inset, for *N* = 399 subjects (all panels display results from *N* = 399 subjects except **d** and **e**, which concern the three 21-subject groups). **b** Histograms of scores across subjects for both patterns, showing raters by color. **c** Bar graph of percent scans of each sex displaying patterns. Chi-squared tests of bursts yield *p* = 3.4e−8 and 8.8e−7 for J.D.P. and C.J.L. (denoted by ***), effects unchanged by excluding members of the three groups. No significant differences are seen by sex in deep breath scores. **d** Bar plots showing mean values with std error bars of the ratings in a clean, burst, and deep breath groups (each with *N* = 21 unrelated subjects). B and D denote burst and deep breath. Desired respiratory properties are found in each group. **e** Algorithm indices of the three 21-subject groups, corroborating rater scores and confirming desired breathing patterns (compare with **d** directly above). Box plots show median and 25th and 75th percentiles as boxes, whiskers encompass 99% of normally distributed data, outliers are individually marked (all box plots in later panels follow this format). **f** Algorithm indices of breathing patterns by sex, with significant differences by two-sample *t*-test in bursts but not deep breaths (compare with **c** directly above). **g** Box plots of algorithm indices for each pattern as a function of mean rater score, demonstrating significant Pearson correlations of humans and algorithm ratings. **h** Box plots of gFC as a function of mean pattern scores, showing much stronger effects of bursts on gFC, quantified by Pearson correlation. **i** Betas of multiple linear regression of pattern scores in gFC (gFC = b0 + b1*burst_score + b2*deep_breath_score), performed in each sex separately, showing much stronger effects of bursts. Bars show mean values, error bars show 95% confidence intervals; fits do not differ by sex (both n.s. by two-sample two-sided *t*-test, uncorrected for multiple comparisons). **j** Box plots of gFC and head size (intracranial volume, ICV) by sex, both significantly different by sex by two-sample two-sided *t*-test (*p* = 9.1e−9 and <1e−20, respectively). **k** Color chart of significance of main effects of multiple ANCOVA models. Sex effects become insignificant when both head size and respiratory variables are modeled. Source data are provided as a source data file, though group identity is redacted.

Identical rater decisions were made in 87% of scans on bursts and in 89% of scans on deep breaths, yielding Cohen’s kappa values of 0.73 and 0.78 overall. Due to the number of ambiguous decisions that must be made in subtle instances of patterns, or amidst disorganized and chaotic breathing styles, we prioritized the totals over four scans in each subject, referred to as pattern scores. These scores correlated at *r* = 0.86 (*p* < 1e−20) for bursts and 0.90 (*p* < 1e−20) for deep breaths between raters, illustrated in Fig. 4a. These numbers indicate that human raters reliably recognize the breathing patterns; interested readers may use the Supplementary pattern training module to learn the patterns (download at https://osf.io/u35f8/).

Deep breaths occurred in about 85% of subjects, and it was common for subjects to display deep breaths in most or all scans (Fig. 4b). Bursts, on the other hand, were absent in about 30% of subjects, and it was relatively uncommon for subjects to display bursts in all scans. In both sets of ratings, chi-squared tests for sex differences were significant for bursts (*p* = 3.4e−8 and 8.8e−7) but not for deep breaths (Fig. 4c), effects unchanged when excluding the groups mentioned above. Overall, bursts were identified in 45% of male scans and 35% of female scans, whereas deep breaths were identified in 54% of male scans and 52% of female scans. Scores of each pattern were uncorrelated across all subjects, for each rater, and within each sex (*r* < 0.1 for all). Thus, the breathing patterns appear to occur independently across subjects. The pattern scores within the 3 previously contrasted groups accord with the desired group breathing properties (Fig. 4d).

### Automated detection

To begin to move beyond rater decisions, which are tedious and time-consuming, we devised an algorithm to index breathing patterns based on joint information in respiratory traces and global fMRI signals. The algorithm creates probabilities of respiratory patterns from the respiratory belt traces and multiplies these probabilities with the match of global fMRI time series to templates of deep breaths and bursts, thus requiring simultaneous evidence from both sources (respiratory trace and global fMRI signal) to begin indexing patterns. This approach performs well in many situations, but haphazard, disorganized breathing can cause high indices on either pattern, thus causing us to algorithmically discount scans with markedly variable respiratory rates. This algorithm recaptures the significant differences in group breathing styles (Fig. 4e) and the significant sex difference in bursts but not deep breaths (Fig. 4f). Indices significantly correlate with rater scores on both patterns (Fig. 4g). These results collectively give good confidence in ratings and group formations and demonstrate a proof-of-principle algorithmic approach to this issue. A fuller description of the algorithm, with illustrations in single scans, is in Supplementary Note 4.

### Influence of respiratory patterns on global covariance

Respiratory events, by influencing cerebral blood flow, add broadly shared variance to all voxel signals, seen repeatedly in this paper as the white and black vertical bands in gray plots. To index such global effects on functional connectivity, in each subject, the median correlation of all gray matter voxel signals in each of the four scans was calculated in the minimally preprocessed data, and the mean of these values over all scans was computed (termed global functional connectivity, gFC).

Robust increases of gFC are seen with increasing rater scores, and the scaling is stronger for bursts (*r* = 0.53, *p* < 1e−20) than for deep breaths (*r* = 0.18, *p* = 4e−4) (Fig. 4h). Similarly, algorithm indices scale with gFC, more for bursts (*r* = 0.59, *p* < 1e−20) than for deep breaths (*r* = 0.29, *p* = 7e−9). The higher values for the indices relative to scores may reflect their ability to scale with prevalence within-run (rather than binary rater decisions), or the fact that the indices incorporate template fits to global fMRI signals. Multiple linear regression of scores and indices yielded betas twice as high for bursts as for deep breaths and additionally demonstrated that fits to gFC for each pattern did not differ by sex (Fig. 4i shows betas (slopes) for score fits to gFC by sex).

Though bursts and deep breaths produce the same effects in gFC in each sex, because bursts are more common in males, gFC may be increased in males relative to females. As Fig. 4j shows, males do have higher gFC. However, males also have larger heads with brains closer to scanner receive coils, meaning signal-to-noise ratios may differ by sex as well, providing an additional potential explanation for gFC differences. We therefore modeled gFC via ANCOVA as a function of sex, head size, and respiratory variables, and only eliminated sex differences when both head size and respiration were accounted for (Fig. 4k). Though motion does not cause global signals (which should largely drive gFC), as a precaution we also added motion covariates (FD_original_, FD_filtered_, and FD_filtered,4-TR_, following^16^) and the data quality covariate DVARS to models 1–4; these additions did not eliminate significant gFC sex differences in any model and often failed to significantly fit as main effects when respiratory variables were present.

### Spatiotemporal effects of bursts and deep breaths in fMRI

**Fig. 5:**
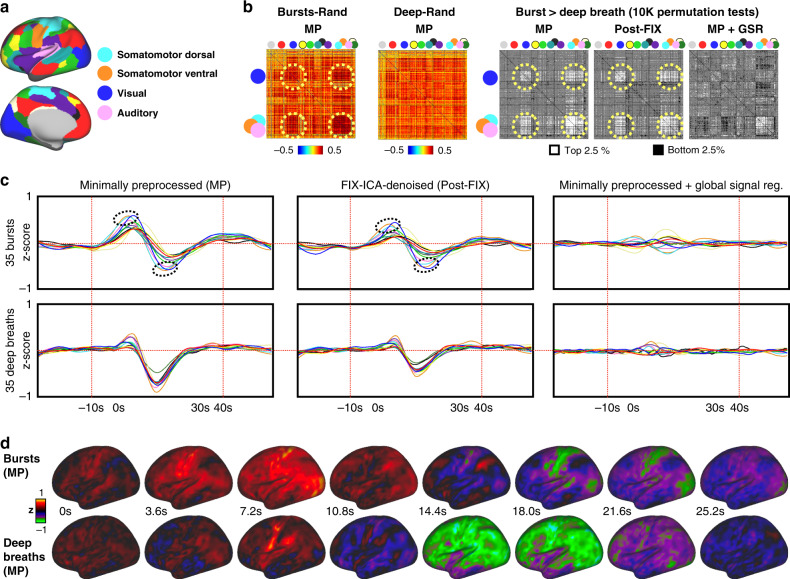
Spatiotemporal properties of bursts and deep breaths. **a** Color legend of network locations and colors from ref. ^17^, with text labeling of the networks of particular interest for this paper (full legend in Supplementary Fig. 11). **b** Correlation matrices are derived from spans of −10 to 40 s about the event onsets shown in Fig. 3 in minimally preprocessed data (red dotted lines in **c**), and show mean differences of 35 bursts and 35 deep breaths compared to 35 random onsets, only coloring cells significant at *p* < 0.05 by 10,000 permutation tests (nearly all cells are significant; non-significant cells are colored gray). In grayscale matrices at right, matrices of bursts were contrasted to deep breaths via 10,000 permutation tests, and the top and bottom 2.5% of actual differences among permutation ranks are illustrated (in white and black; gray is insignificant) in matrices for minimally preprocessed (MP), FIX-ICA-denoised (Post-FIX), and minimally preprocessed time series plus global signal regression (GSR). Differences exist under each processing strategy, prominently including visual, auditory, and somatomotor cortex (blue, pink, orange, and cyan). **c** Mean signals of 35 patterns from each kind of time series in **b**, with mild smoothing, colored by the legend above. Dotted ovals encircle the peaks and troughs of the aforementioned sensorimotor networks. **d** Surface representations of the events, the same data in **b** and **c**, in minimally preprocessed time series. Supplementary Movie 5 animates these patterns and those of non-respiratory motion onsets and random onsets.

We next asked whether there were non-global profiles of the breathing patterns in functional connectivity and whether such profiles differed by pattern. We first focused on covariance during the breathing patterns. Using the sets of 35 events from Fig. 3, we extracted the time series spanning −10 to +40 s about the onsets and computed mean correlation matrices in a commonly used parcellation scheme^17^, illustrated in Fig. 5a. Permutation tests among patterns yielded significant differences of each pattern from random onsets (only cells significant at *p* < 0.05 by 10,000 permutation tests are colored; nearly all cells are significant), and from each other, shown in Fig. 5b for several versions of signal processing. Mean signals within the resting state networks are plotted in Fig. 5c, illustrating the basis of the correlation matrices. Several spatially specific effects are present. For the present purposes, two points are emphasized. First, in all forms of signal processing, significant spatially specific effects exist. Second, in the minimally preprocessed data (and in FIX-ICA-denoised data), which best represents the original respiratory effects, there is a striking elevation of correlations in a primary sensory and motor distribution encompassing visual, auditory, motor, and somatosensory cortex (dotted ovals in Fig. 5b). Signals in these networks peak high and early (dotted ovals in Fig. 5c), and have deep and early troughs, relative to other networks. For completeness, time series are also shown on a brain surface in Fig. 5d (and in Supplementary Movie 5), comprehensively illustrating both global and focal effects in each pattern.

### Covariance associated with bursts and deep breaths

**Fig. 6:**
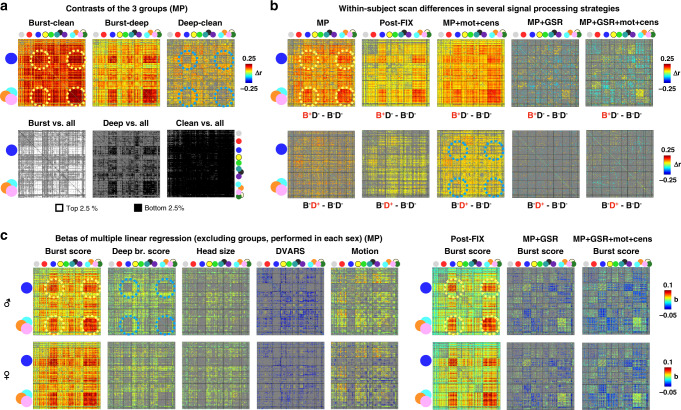
Functional connectivity associated with breathing patterns. All images color only contrasts or differences significant at *p* < 0.05 by 10,000 permutation tests (gray cells are insignificant). **a** Contrasts of the three groups. The top row shows mean differences between groups. The bottom row shows the rank of mean group correlations amidst random, unrelated groups drawn from the entire cohort. **b** Mean within-subject differences between scans without breathing patterns (B−D−) and scans with bursts (B+D−, top row) or deep breaths (B−D+, bottom row) (using only scans where both raters fully agreed). **c** Betas of multiple linear regression performed separately in each sex, using unrelated, non-group-member subjects only. Regressors were *z*-scored, and all betas are shown for minimally preprocessed (MP) data. Betas for bursts alone are shown for several other data processing strategies (Supplementary Fig. 11 shows full sets of betas). In each of these three main analyses (**a**, **b**, **c**), bursts strongly associated with an increase in sensorimotor correlations (yellow dotted circles), and deep breaths lack elevation in these regions (blue dotted circles).

Whereas Fig. 5 focused on effects during breathing patterns, Fig. 6 focuses on correlation structures of entire scans associated with (but not necessarily caused by) the breathing patterns. Effects in minimally preprocessed data are the focus, but some major data processing strategies are also shown. As in Fig. 5, all matrices only color effects significant at *p* < 0.05 by 10,000 permutation tests (gray is used for insignificant cells).

First, we examined the correlation structures of the three groups (Fig. 6a). The burst group had correlations broadly elevated above both the clean and deep breath groups, with especially high elevations in the sensorimotor distribution (dotted yellow circles). The deep breath group had broadly higher correlations compared to the clean group, notably avoiding the sensorimotor distribution (dotted blue circles), despite bursts having no role in these scans or contrasts. Comparing the groups to randomly formed groups drawn from all unrelated subjects recapitulated these findings; these latter analyses can be viewed as contrasts of relatively pure breathing patterns with typical breathing (i.e., randomly selected baseline mixtures of the breathing patterns).

Second, we examined within-subject differences between scans with neither respiratory pattern and scans with either bursts (Fig. 6b, top row) or deep breaths (bottom row). Within-subject burst effects are widespread elevations with sensorimotor emphasis (dotted yellow circles) and are present in minimally preprocessed and FIX-ICA-denoised data, and also in data that undergoes motion regression and censoring (third column). When global signals are removed, significant effects persist but in an altered distribution, reflecting the fact that a lagged signal structure exists in bursts that is not captured in the mean signal. This respiratory effect becomes more pronounced when motion regression and censoring are performed along with global signal regression, underscoring that these patterns are not motion-caused signals. Within-subject deep breath effects, on the other hand, are widespread elevations without global signal regression, which are largely eliminated with global signal regression, reflecting the fact that the major modulation is tightly time-locked and similar in all networks. There is a hint of the sensorimotor non-elevation seen in the group contrasts in certain processing strategies (dotted blue circles).

Third, we examined betas of multiple linear regressions across subjects, separately in each sex (males in the top row of Fig. 6c, females in the bottom row, only unrelated, non-group subjects used). Very similar spatial beta structures were seen in males and females (compare top and bottom rows). Again, bursts associated with global elevations that were especially pronounced in sensorimotor distributions (dotted yellow circles), and deep breaths associated with milder widespread elevations that conspicuously avoided sensorimotor distributions (dotted blue circles). Nuisance effects of DVARS and head motion are congruent by sex and distinct from those of respiratory patterns in all processing strategies (best illustrated in Supplementary Fig. 11).

For convenience, matrices from Figs. 5 and 6 are arranged by respiratory pattern pattern in Supplementary Fig. 12, along with related effects in instructed breathing paradigms.

### Potential influences of sleep or arousal

Here, we attempt to document relationships of the respiratory patterns to arousal or sleep, factors that are well-known to modify breathing. Our ability to address these questions is limited in HCP data, which includes no hard measure of arousal or sleep, but several expected associations of breathing and sleep can be (indirectly) tested. It should first be stated that studies in young adults, whether by polysomnography or by survey, do not find sex differences in delay to sleep in the age range of the HCP subjects (though sex differences in delay to sleep do emerge later in life, after menopause)^18,19^. Large studies of excessive daytime sleepiness also routinely find no sex effect^20,21^. And studies of sleep onset during fMRI in young adults do not report sex effects^22^. There is thus little a priori reason to expect for a sex difference in tiredness or sleep onset to be the cause of sex-biased breathing effects in young adults.

One prediction is that deep breaths should associate with sleep. In the respiratory literature, deep breaths are associated with many factors including tiredness and sleep^13^. No hard measure of sleep exists in HCP data, but a list of sleepy subjects was kept by scanner technicians, which includes 37% of all HCP subjects. Of our three groups, 71% of the deep breath group was on that list (*p* = 0.0014), compared to 38% of the clean group (n.s.), and 41% of the burst group (n.s.), giving face plausibility to the validity of the list and indicating that subjects exhibiting many deep breaths are likely enriched for people yawning. Relatedly, we examined gray plots of individual scans in which subjects were documented as definitely sleeping (outside the groups) and we could discern no visual signature of sleep, certainly not by respiratory pattern.

Another testable proposition is that sex-biased bursts are merely snores or obstructive sleep apnea. Obstructive sleep apnea is strongly potentiated by obesity^23^, and there was no correlation between burst scores and body mass index (BMI) in either sex (male *r* = 0.07, *p* = 0.32; females *r* = −0.03, *p* = 0.66), nor did males and females significantly differ in BMI, which was in the mid-20s for both sexes. Collectively, these results support central, not obstructive, causes of bursts (though some instances could be obstructive).

Another testable proposition is that the breathing patterns become more likely as scans progress, perhaps reflecting an influence of arousal (if not necessarily sleep). Our visual impressions from examining all scans were that deep breaths seemed to occur at any point of a scan, including the very beginning, and were not notably concentrated at the end, whereas bursts seemed to be uncommon at the beginning of scans and to emerge later in scans. More formally, in both males and females, algorithm indices for both patterns rose as scans progressed, and *t*-tests of indices in minutes 1–4 compared to 11–14 of scans were significant for both patterns in both sexes (both *p*’s < 1e−16 for bursts, both *p*’s < 0.02 for deep breaths). These statistical effects accord with our visual impressions.

Collectively, these observations indicate that deep breaths are associated with sleepiness, that bursts are probably central rather than obstructive phenomena, and that sex biases in sleep onset or tiredness are unlikely to be causes for sex differences in bursts. They also indicate that bursts become more likely as scans progress in both sexes.

## Discussion

In this paper, we described two respiratory patterns that differentially bias functional connectivity, both commonly seen in young adults lying in MRI scanners. This work represents our first major effort toward developing an event-related framework for understanding effects in spontaneous fMRI time series, following argumentation in ref. ^24^. Distinct respiratory effects can occur within-scan, between scans in subjects, and across subjects and populations. This discussion links respiratory patterns to effects in the neuroimaging literature and lays out a potential mechanistic basis of bursts that could explain why bursts occur more in males. It concludes by noting settings in which differential expression of respiratory patterns may be anticipated. A Supplementary Discussion elaborates on concepts described only briefly in the main text, especially regarding mechanisms and clinical associations of periodic breathing.

Both deep breaths and bursts had brain-wide effects on fMRI signals, causing increased global functional connectivity, and the effects were most marked for bursts. The increased prevalence of bursts in males contributed to higher average fMRI signal correlations compared to females. These differences were eliminated once both brain volume and respiratory patterns were accounted for. To clarify, we are not broadly asserting that all functional connectivity sex differences are due to respiration or even necessarily that the specific differences we removed were due to respiration (a third shared variable could be at work); we are asserting that respiration is capable of causing sex differences in functional connectivity.

The respiratory patterns had distinct spatiotemporal effects, with bursts strongly elevating correlations among sensorimotor brain regions, whereas deep breaths had their weakest effects in such regions. Signal modulations were fairly time-locked across the brain in deep breaths, but bursts unfolded with multi-second lags between different parts of the brain. These distinctions, joined with distinct cardiac effects, indicate that distinct cardiopulmonary and neurophysiological events are occurring during the patterns. An important topic of future work will be to disentangle pCO_2_-related cerebral blood flow effects during each respiratory pattern from the blood flow consequences of the neural activity that triggers and tracks and guides each pattern.

It is noteworthy that the sensorimotor pattern has emerged in numerous areas of fMRI research, including studies localizing global fMRI signals at rest^25,26^, and studies of arousal and sleep^27–30^. The extent to which bursts (or deep breaths) define what is measured as the global fMRI signal is presently unknown. Given the lively debates over retaining or discarding global fMRI signals, it will be important to understand how various denoising or data processing strategies impact one or both of these respiratory patterns. Similarly, it will be of interest to examine these phenomena in the context of caffeine administration, vigilance tasks, lag structure, or quasiperiodic patterns (e.g., refs. ^31–34^). As detailed below, there are reasons to suspect that psychiatric symptoms will also scale with these patterns; indeed, our clean group without bursts or deep breaths reported a colloquially “clean” lifestyle, with unusually little substance use or psychiatric symptomatology (Supplementary Note 3).

By our estimate, in young adults, deep breaths occurred in about half of all scans, and in well over three-quarters of subjects, numbers that are unsurprising from a respiratory perspective. Adult humans inhale deeply several times an hour to counter mild changes in blood gas tensions and to reinflate collapsed alveoli, and such physiologic sighs are especially common in the supine position used for scanning^13^. In addition, yawns are likely to occur in some subjects.

The burst pattern occurred in over one-third of scans and in two-thirds of subjects, with a tendency to occur in males. The lack of correlation between BMI and burst prevalence in either sex suggests a central rather than obstructive origin of the pattern, though some instances might be obstructive. A centrally oriented explanation is that the patterns arise via sex-biased respiratory control parameters that are unmasked as subjects relax in the scanner. Our leading hypothesis is that the pattern emerges via parameters governing the chemoreflex, which could explain both the waveforms and the sex bias of bursts, as detailed below.

**Fig. 7:**
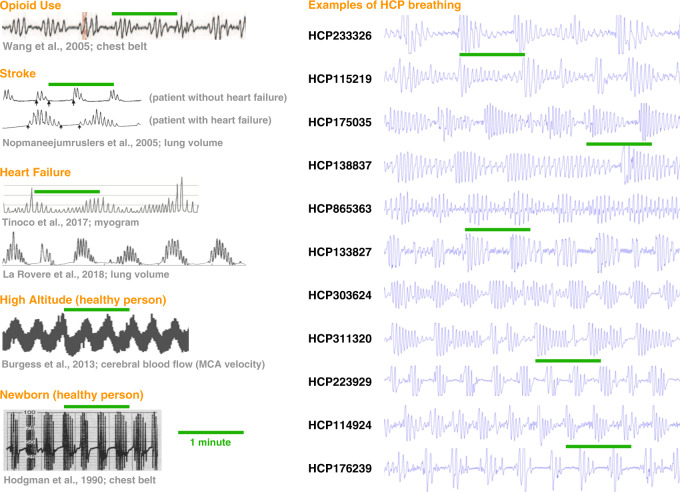
Comparison of periodic breathing waveforms and bursts. At left, single-subject waveforms of periodic breathing in opioid use, stroke, heart failure, at high altitude, and in newborns. These are all conditions and situations in which periodic breathing is commonly encountered. At right, illustrations of bursts in 11 HCP subjects. All plots are on the same time scale, and the green lines measure 1 min. Note the long cycle times seen in patients with heart failure (in the Stroke and Heart failure sections), reflecting, in part, an exaggerated delay in central detection of changes in arterial gas tensions (see Supplementary Discussion for more detail). The stroke example illustrates shows the added effect of delayed circulatory time in heart failure. Images at left modified from^46,54–58^ with permission. Images from ref. ^55^ adapted with the permission of the American Thoracic Society. Copyright © 2020 American Thoracic Society. All rights reserved. *The American Journal of Respiratory and Critical Care Medicine* is an official journal of the American Thoracic Society. Readers are encouraged to read the entire article for the correct context at https://europepmc.org/article/med/15665317. The authors, editors, and The American Thoracic Society are not responsible for errors or omissions in adaptations.

The generation of waxing and waning patterns of breathing occurs via interactions among the mechanisms controlling breathing (this discussion is simplified, see the Supplementary Discussion and ref. ^35^ for detail); when such patterns attain cyclic stability they are called periodic breathing (Fig. 7 shows periodic breathing in several situations in which it is routinely seen). Breathing rhythms are generated in the medulla and are under three kinds of control: volition, a waking neural drive during wakefulness and rapid eye movement (REM) sleep, and a chemoreflex loop that centrally senses pH and thereby pCO_2_^8^. The chemoreflex is central to this discussion. Every person has preferred set points for arterial pCO_2_, called the resting pCO_2_, and fluctuations about resting pCO_2_ engage a negative feedback loop: at high arterial pCO_2_, the chemoreflex stimulates breathing in order to reduce pCO_2_, but below a certain value of pCO_2_—the apneic threshold—the chemoreflex ceases to stimulate breathing. Cyclic waxing and waning of respiration generally occur in the following manner: (excessive) ventilation pushes pCO_2_ below the apneic threshold, causing chemoreflex respiratory drive to fade or cease, after which pCO_2_ rises and pO_2_ falls, eventually triggering the resumption of (excessive) ventilation and the start of the next cycle.

Key to initiating and propagating such cycles is the CO_2_ reserve – the difference between resting pCO_2_ and the apneic threshold – which exhibits sex differences due to gonadal hormones. Women have the same resting pCO_2_ levels as men, but lower apneic thresholds, and thus larger CO_2_ reserves^36^. Sex hormones influence these parameters: administration of testosterone to women raises the apneic threshold without altering resting pCO_2_, thus reducing CO_2_ reserve^37^. Smaller CO_2_ reserves mean a smaller ventilatory perturbation is needed to trigger apnea and cycles of waning and waxing breathing, and thus larger CO_2_ reserves make apneic events less likely, leading to the expectation that women should be less likely to initiate and perpetuate periodic breathing than males. In a recent study, when healthy adults were taken to high altitude (Mount Everest), forcing a lowered resting pCO_2_ (due to hyperventilation), women displayed far less central apnea, and periodic breathing specifically, than men^38^. These manipulations (testosterone, hypoxia) highlight the role of sex hormones in shaping chemoreflex responses, which is the basis of a well-established tendency for females to display less apnea than males. Waxing and waning cycles of breathing are usually studied during sleep and measured via the apnea/hypopnea index (AHI), indexing the number of events per hour, and numerous large respiratory studies detect robust sex differences in AHI scores, with women displaying fewer episodes than men^39–42^. Further evidence of the role of sex hormones in apnea is that AHI sex biases are reduced after menopause, that hypogonadal males have lower AHI than weight-matched peers^43^, and that women with testosterone-producing tumors have higher AHI than weight-matched peers^44^.

In short, the sex biases of the burst pattern could potentially arise via sex-hormone dependent properties of the chemoreflex loop that potentiate sex differences in apnea and hypopnea. Such disordered breathing is—by far—most commonly studied in older, clinical populations and during NREM sleep, when the chemoreflex properties are maximally exposed (although recent studies have begun to document such breathing in the daytime^45,46^). Though lessening of the wakefulness drive would help to reveal instabilities in the chemoreflex loop, the extent to which sleep or reduced arousal is contributing to bursts in the HCP data is unclear, and new datasets with concurrent monitoring of respiration and arousal will be needed to properly address the issue. The issue is important because brief arousals can result from hypoxia after apnea, and can perpetuate new cycles of central apnea by transiently (excessively) increasing ventilation. The variety of forms that periodic breathing can take, and their relation to multiple parameters of the chemoreflex loop and resemblances to bursts, are discussed at greater length in the Supplementary Discussion.

The influence of breathing on fMRI signals is marked, and our results suggest situations where breathing biases may be expected. The Supplementary Discussion reviews literature suggesting the following associations. Yawns will be more prevalent in tired subjects, and in subjects on medications causing sedation. To the extent that bursts share the respiratory mechanisms that drive increased AHI in the respiratory literature, one would anticipate increases in bursts (1) over the lifespan in both sexes (especially after age 60), (2) in males relative to females at all ages (a bias lessening after menopause, probably with onset around menarche), (3) in subjects on opioids and other respiratory suppressants, (4) in subjects with brain injuries (e.g., strokes), and (5) in subjects with medical conditions like heart failure. Large psychiatric studies have reported longitudinal dose-response associations of AHI with depressive symptoms^47^, and community samples routinely obtain cross-sectional associations of depression and AHI^39^. Fluctuations in gonadal hormones over pregnancy, over menstrual cycles, or by sex-hormone therapies are also likely to influence burst prevalence. Medications influencing cholinergic, noradrenergic, and serotonergic inputs to wakefulness respiratory drive may influence the emergence of bursts, and may have a role in the association of depression and AHI scores. Given the relative ease of using the respiratory belts provided with many scanners, it would be prudent to collect such physiology records during fMRI scans (see ref. ^14^ for discussion of implementations).

## Methods

### Data and subject selection

The 900-subject public release of the Human Connectome Project (HCP) Young Adult data was obtained. Subjects were young adults drawn from the geographic region about St. Louis, Missouri, and were identified via a Missouri state registry of siblings sets that include twins. Subjects were healthy in a broad sense: study criteria excluded sibships containing siblings with diabetes, hypertension, or neurological or psychiatric disorders. The HCP-YA protocol was approved by The Washington University in St. Louis Institutional Review Board, WUSTL DHHS Federalwide Assurance #FWA00002284 BJH DHHS Federalwide Assurance #FWA00002281 SLCH DHHS Federalwide Assurance #FWA00002282, and the study was conducted in accord with the Declaration of Helsinki. Subjects provided written consent to participate and were compensated monetarily for participation. Each subject underwent four 14.4-min fMRI scans (two scans on two days each) while staring quietly at a crosshair at an altitude of ~450 feet above sea level. During scanning, physiology data was acquired via the Siemens Physiology Monitoring Unit (PMU), which is standard equipment that accompanies the scanner for purposes of cardiac and physiological gating. The signals acquired were 400 Hz recordings of an abdominal belt and a finger pulse oximetry waveform. The Siemens respiratory record is obtained via a pressure hose connected to a respiratory cushion placed under an elastic belt strapped around the subject’s abdomen, and output is in arbitrary units. All resting-state fMRI scans were obtained, including minimally preprocessed and FIX-ICA-denoised images, along with their accompanying head position and physiology files.

Of the HCP 900-subject release, based on visual assessment of the physiology traces, 440 subjects had four 14.4-min resting-state fMRI scans with complete accompanying physiological data in which we believed we could reliably identify peaks in all cardiac and respiratory traces (all physiological data and decisions about quality can be seen in the Supplementary material of ref. ^48^). Only these 440 subjects were analyzed further. Characteristics of the subjects were: age 28.6 ± 3.8 (range 22–36), 228 males and 212 females; BMI 26.5 ± 5.0 (range 16.5–43.9).

For the 440 subjects believed to have high-quality physiology data, the following files were obtained: four resting-state fMRI scans transformed to atlas space (in each subject’s/MNINonLinear/Results folder): [RUN] = REST1_LR, REST1_RL, REST2_LR, REST2_RL (this order is runs 1–4 in the text). rfMRI_[RUN].nii.gz and rfMRI_[RUN]_hp2000_clean.nii.gz scans were obtained, representing minimally preprocessed and FIX-ICA-denoised data. For each of these scans, the [RUN]_Physio_log.txt and Movement_Regressors_dt.txt files were also obtained. Structural scans transformed to atlas space were also obtained (in each subject’s /MNINonLinear/folder): the T1w.nii.gz and the aparc+aseg.nii.gz files, representing the anatomical T1-weighted scan and its FreeSurfer segmentation.

### Image and parameter processing

The aparc+aseg.nii.gz file for each subject underwent a set of serial erosions within white matter and ventricle segments, exactly as in ref. ^25^. Masks of cortical gray matter, the cerebellum, and subcortical nuclei were extracted, as were serially eroded layers of superficial, deep, and deepest (with respect to distance from the gray matter) masks of the white matter and ventricles. These masks, together, include all in-brain voxels of these tissue types and are used to extract certain signals and to order signals for gray plots^49^. For the purpose of making useful gray plots, because of the considerable thermal noise in HCP scans, a within-mask 6 mm FWHM Gaussian kernel was applied to the data using the above masks (illustrated for HCP data in ref. ^49^). This blurring does not mix tissue compartments, due to the use of masks, beyond partial volume effects present in the voxels themselves.

Respiratory belt and pulse oximeter traces (sampled at 400 Hz) first underwent visual inspection in their entirety to determine if the quality was sufficient for reliable peak detection since traces are often partially or fully corrupted. Only subjects with traces deemed likely to successfully undergo peak detection in all runs were analyzed^48^. After selection, for respiratory traces, an outlier replacement filter was used to eliminate spurious spike artifacts (Matlab command: filloutliers(resp_trace,‘linear’,‘movmedian’,100)) and the traces were then gently blurred to aid peak detection (Matlab command: smoothdata(resp_trace,‘sgolay’,400)) (a 1-s window for a 400 Hz signal). These treated respiratory traces are the ones shown in Figures.

Following prior literature, several respiratory measures were derived from the treated respiratory belt trace. First, the envelope of the trace over a 10-s window (at 400 Hz) was calculated after ref. ^15^ (Matlab command: envelope(zscore_resp_trace,4000,‘rms’)). Second, the RV measure, defined as the standard deviation of the treated respiratory trace within a 6-s window, was calculated following^12^ (Matlab command: movstd(zscore_resp_trace, 2400,‘endpoints’,‘shrink’)). Finally, an RVT measure, defined for all peaks as ((peak-prior trough)/(time between peaks)), was calculated. Peak detection on the trace yielded peaks (and troughs, using the inverted trace) for calculations (Matlab command: (findpeaks(zscore_resp_trace,‘minpeakdistance’,800,‘minpeakprominence’,.5))). The minimum peak distance presumes breaths occur more than 2 s apart. If a peak did not have a preceding trough prior to the previous peak, no value was scored at that peak. All traces and derived measures were visually checked to ensure that outliers and abnormalities would not drive results. These three measures were termed ENV, RV, and RVT in figures. The RVT calculated is the “core” computation studied in ref. ^14^, which behaves like the full computation of ref. ^10^.

Pulse oximeter traces underwent *z*-scoring then peak detection (Matlab command: findpeaks(zscore_pulseox,‘minpeakdistance’,180,‘minpeakprominence’,.5)). Heart rate was calculated from the interval between peaks. The minimum peak distance presumes heart rates are under 133 beats per minute. Peak amplitude was calculated from the height of the peak relative to the previous trough. Cardiac traces are prone to transient disruptions when fingers move, and it is laborious to check and correct cardiac measures due to the large numbers of peaks and troughs. A limited number of cardiac records are therefore used in this report, but those select traces and their derived measures were visually checked to ensure accuracy.

The data quality measure DVARS was calculated after refs. ^50,51^ as the root mean squared value in the brain at each timepoint of all voxel time series differentiated in time by backward differences. DVARS by convention is 0 at the first timepoint.

Head position was taken from the Movement_Regressors_dt.txt files. In gray plots, these position parameters are displayed after subtracting the first timepoint value from the time series (so that all traces start at zero). Head motion was represented by Framewise Displacement (FD) measures, following ref. ^51^, wherein all position measures were differentiated in time by backward differences, rotational measures were converted to arc displacement at 5 cm radius, and the sum of the absolute value of these measures was calculated. To suppress tidal respiratory motion, head position traces were filtered with a stopband of 0.2–0.5 Hz following ref. ^16^. FD is typically calculated by backward differences to the preceding timepoint (here 720 ms prior), but historically FD measures using sampling rates of 2–4 s were common; for comparison to such measures, FD was also calculated by backward differences over four timepoints (4 * 720 ms = 2.88 s effective sampling rate) where indicated, exactly as in ref. ^52^. Thus, where indicated, head motion measures of FD_original_, FD_filtered_, and FD_filtered,4-TR_ are examined.

### Gray plot formation

Gray plots were created of each scan, exactly as shown in Figs. 1 and 2, and exactly following procedures outlined in refs. ^14,16^. Examination of these 4 * 440 = 1,760 gray plots led to the recognition of the burst pattern described throughout the paper, as well as the single deep breaths also characterized.

The 35 bursts, 35 deep breaths, and 35 non-respiratory motions shown in Fig. 3 were chosen by visual inspection of respiratory and motion records, with visually marked onsets shown in Supplementary Movies 2–4 and onsets listed in Supplementary Data 1. Random onsets were defined in the motion subjects using randomly selected runs and onsets. Properties of scans in the 90 s surrounding the onsets are shown in heat maps. Thin gray bars with red heat maps show the significance of unpaired two-sample *t*-tests at each timepoint compared to the random onset group. Heart rate was derived from peak-to-peak intervals in pulse oximetry data and was visually verified. Plots show the mean properties of the 35 onsets, and shade plots show mean and standard deviation.

### Group formation

Author JDP examined all 440 subjects, noting subjects with many scans with only deep breaths, many scans with only bursts, or many scans with only normal tidal breathing patterns. These lists were then screened for any siblings, and siblings were discarded in a manner yielding the largest equally-sized remaining groups, resulting in three groups of 21 subjects, all unrelated. Statistical contrasts of the group demographic and other properties are described in the Supplementary Materials. Because of associations reported to medical and psychiatric variables, the identities of these groups are restricted to registered HCP users who have been granted Restricted Access, via a Subject Key. To access the Keys: (1) Sign in to https://db.humanconnectome.org; (2) Navigate to and open the WU-Minn HCP data set by clicking the “Open Dataset” tab; (3) Click the “Subject Keys” tab; (4) There are three subject keys listed under “Published Subject Keys for This Dataset” associated with this manuscript. Click on a subject key to obtain a description and access the data.

“Lynch_etal_2020_NatureCommunications_BurstGroup”

“Lynch_etal_2020_NatureCommunications_DeepBreathGroup”

“Lynch_etal_2020_NatureCommunications_CleanGroup”

Note**:** Only HCP users with restricted data access will be able to use subject keys. If you receive an error message (e.g., “Restricted Data Access Required!”) you must request restricted data access.

### Ratings of breathing patterns in scans

Two authors (J.D.P. and C.J.L.) jointly examined the scans of subjects 400–440 to discuss breathing patterns, then independently rated scans 1–399 with binary decisions in each scan about the presence (1) or absence (0) of deep breaths and bursts in each scan. Cohen’s kappas were calculated for the ratings, shown in Fig. 4, yielding high inter-rater reliability. The likelihood of obtaining sex differences in each breathing pattern was determined by Chi-squared tests in each rater, and both raters obtained sex effects of bursts but not of deep breaths. The sum over the four scans in each subject of each type of breathing pattern was called the pattern score.

### Algorithmic indexing of breathing patterns

The automated algorithm is described in detail in the Supplementary Materials. In brief, priors were derived from respiratory belt traces indicating, separately, the likelihood of deep breaths and bursts, and these priors were multiplied into the fits of fMRI templates of each breathing pattern to global fMRI signals. The total values over each scan for each breathing pattern were obtained and averaged in each subject. Both indices were downweighed by the variation in breathing rate of a scan, to dampen outliers due to haphazard breathing. Indices were correlated to rater pattern scores and were compared in each breathing pattern by sex using unpaired two-sample *t*-tests in Fig. 4.

### Functional connectivity measures

We first describe global functional connectivity (gFC) analyses. The pairwise correlations of all voxels within a subject’s gray matter mask were computed in each fMRI scan, and the median value in each scan was taken, followed by Fisher-*Z* transformation. These values represent the central tendency of gray matter functional connectivity, which is what one would expect for respiratory phenomena to most directly influence, since cerebral blood flow changes have brain-wide consequences for fMRI signals. These values were averaged over a subject’s scans for the purposes of cross-subject correlations, linear regressions, and ANOVA/ANCOVA in Fig. 4. gFC and rater scores were compared by correlation. Differences by sex were compared by unpaired two-sample *t*-test. gFC was fit to pattern scores separately in males and females using the formula gFC = b0 + b1*burst_score + b2*deep_breath_score, yielding similar fits in each sex. In Fig. 4k, ANOVA (model 1) and ANCOVA (models 2–6) was used to model gFC as a function of sex alone or sex plus other variables, and main effects of the explanatory variables are color-coded in Fig. 4k. Model 1 is an ANOVA with only sex, Model 2 adds head size in ANCOVA, and the other models are ANCOVAs with the indicated terms present, always significantly fit, with the exception of the sex variable, which becomes insignificant in the final two models (green cells). DVARS and the three versions of head motion were added to models 1–4 but failed to negate the sex effect in any model, and often failed to even fit as main effects when respiratory variables were present.

Here we describe network analyses. The 333-parcel scheme of Gordon et al.^53^ was used to sample images, and the cluster assignments of that paper define the resting-state networks in this paper, illustrated in Fig. 5a (see Supplementary Fig. 11 for a full list of networks). Correlation computations were performed via Fisher-*Z* transforms but were converted to Pearson *r* values for figures and reporting. For all matrices in Figs. 5 and 6, and Supplementary Figs. 11 and 12, only cells significant at *p* < 0.05 are colored (always determined by 10,000 permutation tests specific to the matrix; gray cells are insignificant).

Minimally preprocessed (MP) and FIX-ICA-denoised time series were sampled, and additionally several signal processing steps were applied to generate a few additional commonly used kinds of signals: MP with the mean gray matter signal removed (MP+GSR, global signal regression), MP data with six motion regressors and their derivatives regressed out and censoring timepoints with *z*-scored DVARS (from minimally preprocessed data) values >2 (MP+mot+cens), or both of those steps applied to MP data along with GSR (MP+GSR+mot+cens). These maneuvers simply give readers a broad sense of when, where, and how severely certain effects manifest in different signal processing regimes.

In Fig. 5, using the sets of 35 onsets defined for Fig. 3, parcel time series from −10 to +40 s about the onset were used to generate correlation matrices for each kind of onset (burst, deep breath, motion, random), and the mean values for breathing patterns are shown, masked by significance in permutation tests with the random matrices. Contrasts of breathing patterns are shown in several data processing strategies (permutations between pattern matrices). Mean parcel time series are shown in Fig. 5c, with red dotted lines denoting the span used for correlations. Mean values of grayordinates are shown in MP data in Fig. 5d and Supplementary Movie 5.

In Fig. 6, correlations from entire scans are used. Figure 6a illustrates the subtraction of mean matrices of the groups, screened by permutation tests between groups. The bottom row of Fig. 6a shows the values of the observed group matrices among random groups drawn from all subjects. Figure 6b illustrates the within-subject differences between scans with and without certain breathing patterns. The top row shows scans with bursts and not deep breaths (B+D−) compared to scans without either pattern (B−D−), the bottom row a comparable contrast for deep breaths. Only scans in which raters had completely agreed on the presence or absence of patterns were used, and all unrelated qualifying subjects possessing the needed scan subtypes were used in each kind of contrast, with 50–70 subjects in each contrast. Mean differences are shown in several data processing strategies, masked by permutation tests with randomly swapped scan subtype labels. Figure 6c illustrates betas in multiple linear regression of breathing pattern scores with head size and nuisance variables, all scaled identically and performed separately in each sex, with all regressors standardized prior to running the model, and masked by significance among betas from randomly permuted subjects. Betas for bursts are shown for several processing strategies. Full fits to all variables are shown in Supplementary Fig. 11 for several processing strategies.

### Reporting summary

Further information on research design is available in the Nature Research Reporting Summary linked to this article.

## Supplementary information

Supplementary Information

Description of Additional Supplementary Files

Supplementary Data 1

Supplementary Data 2

Supplementary Movie 2

Supplementary Movie 3

Supplementary Movie 4

Supplementary Movie 5

Reporting Summary

## Data Availability

HCP data are publicly available at www.humanconnectome.org. Source data are available at https://osf.io/u35f8/ (10.17605/OSF.IO/U35F8). That link also contains all Supplementary movies (~2 GB). Certain HCP data are restricted to protect subject privacy, such as genetic, medical, and neuropsychiatric information. The three 21-subject groups of this paper demonstrate associations to such variables, and their identities are only available by Subject Key within HCP Restricted Access accounts and are otherwise obscured in the publicly available data. Source data are provided with this paper.

## Source data

Source Data

## Code availability

Matlab-based gray plot modules to train users on breathing patterns, Matlab code implementing the automated indexing of patterns presented in this paper, and Matlab code recreating certain figures including inputs and sample output is available at https://osf.io/u35f8/ (10.17605/OSF.IO/U35F8). Certain elements of our original code are redacted to protect the privacy of the above-mentioned groups.
