# Supplementary material for: Prevalent and sex-biased breathing patterns modify functional connectivity MRI in young adults
Source: Nat Commun. 2020 Oct 20;11:5290. doi: 10.1038/s41467-020-18974-9 (PMC7576607; doi:10.1038/s41467-020-18974-9)
Supplement: Supplementary file 1 — Supplementary Information [file 41467_2020_18974_MOESM1_ESM.pdf]

Supplementary Methods

Instructed breathing data. These imaging data are reported in (1, 2). For the single subject of Supplementary Figure 12A, on 5 consecutive days a single subject was scanned using a multi-band multi-echo CMRR sequence while performing 4 widely spaced deep breaths 70 seconds apart, with visual cues to breath, for a total of 20 instances of instructed deep breaths. After multi-echo ICA denoising, the 30 seconds (-5 to 25 sec) around the breath were extracted, and 30 seconds (30-60) following that breath were also selected, forming epochs with and without deep breaths. Correlations, differences, and permutations follow the form of Figure 5B. For reference and comparison, a single HCP subject with 10 deep breaths is shown, with similar waveforms (but much lower signal-to-noise due to small voxels, fewer events, and single-echo sequence).

Supplementary Note 1

Factors influencing detection of bursts and temporal occurrence of bursts. We have previously examined in detail the resting state fMRI scans of some 120 subjects using techniques very similar to those used in this paper, totaling over 12 hours of scan time (3-5). If bursts have such pronounced properties, why have we not previously noticed them? Several factors contributed.

First, our previous scans (often 6-8 min) were too short to permit more than a handful of bursts to occur, whereas the HCP scans are long enough that a dozen or more bursts may occur, making bursts more recognizable as a stereotyped phenomenon. We did note substantial modulations of respiratory depth previously, and some of these modulations in retrospect we would now call bursts (e.g., the bottom left scan in Figure 6 of (3)), but we did not understand (or consider) at the time that the modulations might be a more general phenomenon.

Second, it was the occurrence of a few pristine examples of bursts in the HCP data that drew our attention to the phenomenon, which then became easier to recognize more generally. Though the respirations illustrated in this paper are chosen for their clarity, most subjects exhibit a mixture of these patterns as well as a variety of other respiratory irregularities that defy easy categorization. A brief examination of the gray plot movie of all subjects will quickly make the variety of respiratory waveforms in most scans evident to any reader. The large size of the HCP dataset thus facilitated recognition of the burst pattern by providing a small number of unmistakable examples: of the 1,760 scans we examined, probably less than 30 had bursts as sustained and distinct as the bursts illustrated in Figure 1.

Third, both our anecdotal impressions and the algorithm indices suggest that bursts become more prevalent towards the middle and end of the 14.4-minute HCP scans (unlike deep breaths, which seem to occur anywhere in a scan period, and have only a very mild propensity to increase with time in the scanner in our algorithm indices, see end of the main Results). In this sense, if bursts are uncommon in the first few minutes of scanning, there may be a disproportionately low chance of observing the pattern in shorter scans compared to longer scans (our prior scans were mostly 6-8 minute scans). Two examples of intra-scan emergence of

burst patterns emerging around minutes 10 and 12 are shown in Supplementary Figure 6, and many other similar examples exist.

In short, we believe we overlooked bursts in previous studies because our relatively short scans gave a limited opportunity for bursts to appear serially (especially if bursts are relatively uncommon earlier in scans or scanning sessions), and because the HCP data is an order of magnitude larger than our prior datasets, providing many clear instances of the pattern.

Supplementary Note 2

Cardiac correlates of bursts. Cardiac correlates of deep breaths but not bursts were seen in Figure 3. We had visually checked the pulse oximetry data of Figure 3, eliminating spurious calculations as a potential cause of absent burst heart rate effects. We additionally examined many scans individually to see if we somehow selected bursts in a way that missed an effect (though our burst instances were chosen without any knowledge of cardiac properties). In the HCP data, intermittent examples can be found of bursts modulating heart rate (typically an elevation in rate at onset of the burst), but frequently in bursts there is no apparent relationship to heart rate (Supplementary Figure 8). This intermittent, unreliable cardiopulmonary relationship in bursts stands in contrast to the reliable modulations of heart rate by deep breaths. We additionally examined our prior datasets in which we had noted sinus arrhythmia and deep breath effects and identified (in retrospect) several subjects with burst patterns (Supplementary Figure 9). Few scans exhibited a notable, systematic relationship with heart rate, which was small increases in heart rate at the start of the burst, similar to what was sometimes observed in HCP data (Supplementary Figure 9). We were unable to discern any property of a burst that would indicate that it would or would not have a heart rate correlate. These modest and intermittent heart rate findings of bursts are consistent with studies in periodic breathing, where heart rates are sometimes, but sometimes not, subtly increased with the cessation of hypopnea and onset of hyperpnea (6).

Supplementary Note 3

Contrasts of single deep breath, burst, and clean groups of subjects. This paper documents two distinct kinds of respiratory patterns. The deep breaths should mainly comprise physiologic sighs, which are universal in humans and serve to counter hypoxia and the natural deflation and collapse of lung compartments over time, and the form of sighs called yawns, which one would expect to more closely relate to fatigue or drowsiness. We know of no way to discriminate these two phenomena within deep breaths given the present data. The burst pattern is possibly compatible with ventilatory control instability during state fluctuations between wakefulness and light sleep, which would fit the tendency of this pattern to occur later in scans. Our initial suspicion was thus that both deep breaths and bursts might relate to drowsiness or alertness. However, a common explanation for both patterns would suggest that the patterns would tend to co-occur in runs or subjects, which does not appear to be true (Figure 4). The independence of these respiratory patterns instead suggests that there may be distinct processes or factors that scale with these respiratory phenomena. For exploratory purposes, we chose to examine

three select, well-defined groups: subjects with marked bursts in most or all scans and few or no single deep breaths, subjects with marked single deep breaths in most or all scans but few or no bursts, and subjects whose scans displayed few to no bursts or single deep breaths.

Respiratory traces and signal heat maps were examined to identify the three groups of subjects above; selection was made without knowledge of demographic or behavioral characteristics, and without knowledge of summary motion or respiratory measures. This procedure initially identified 21 subjects per group, all unrelated. Each of these subjects had plentiful burst or single breath patterns throughout their scans or exhibited neither pattern (the clean subjects).

To establish null expectations for finding significant differences in groups of 21 subjects, hundreds of 21-subject cohorts were randomly formed from all unrelated subjects and then contrasted with 10,000 randomly formed 21-subject cohorts drawn from the same subjects. Significant differences were defined by means in the cohort of interest among the 250 lowest or 250 highest values found among the 10,000 permutations. Across 782 variables, these contrasts tended to yield 32 significant differences, with a range of 21-47 over several hundred random cohorts-of-interest. 25 of these random results are shown in the heat map in Supplementary Figure 10. When the three groups of interest were contrasted via the same procedure, bursts yielded 31 significant variables, single deep breaths yielded 62 significant variables, and clean scans yielded 72 significant variables. These results are shown at the bottom of the heat map (results are listed in detail in Supplementary Table 2). In the heat map, it is apparent that the variables are clumped in unusual ways in the groups of interest (variables are listed in order of the HCP spreadsheets, which groups variables by kind).

The most striking findings are collections of strong, uniform associations with structural brain images in each of the groups of interest. Single deep breath and clean groups each have dozens of associated FreeSurfer brain volume and cortical surface area measurements, which are uniformly lower in these subjects. The burst group exhibits a distinct structural finding: nearly a dozen variables uniformly exhibit decreased cortical thickness measurements. These structural variables are notable both for their number and for their uniformity in the directionality of the effect within each group. Random cohorts-of-interest displayed far fewer structural findings, and they were always directionally mixed (i.e., thicker in one location, thinner in another).

The behavioral and physiological findings for the groups are less striking but are reported for completeness and because there are some suggestive associations that could be tested in future, separate subjects. The clean group reports falling asleep quickly at night and sleeping well, reports low alcohol use, reports starting tobacco use late in life (if ever), reports little pain interfering with their lives, and scores low on the Achenbach Adult Self Report in terms of the total score, the number of critical items (e.g., can't sit still, cries, sees things, harms self, etc.), and on specific indices of rule-breaking behavior, externalizing traits, and antisocial traits. These variables are notable for their uniform directionality pointing towards a "clean" lifestyle in the colloquial sense, even though the subjects were identified purely by respiratory patterns and associated fMRI signals; in the clean group there are no variables pointing towards psychiatric or physical suffering or substance use. By contrast, the burst group has associations with

increased alcohol consumption, alcohol dependence, and higher blood sugar levels, and the single deep breath group has associations with same-day tobacco use and low hematocrit. It is worth reiterating that sibships with diabetes and hypertension were excluded from the HCP study and thus sensitivity to measures related to those conditions is limited. Other variables are associated with the groups (all are listed in Supplementary Table 2), but the ones mentioned here are the most prevalent and salient.

The structural imaging variables comprise roughly half of the associated variables across all groups, and distinct patterns distinguish bursts (decreased cortical thickness) from single deep breath and clean scans (decreased volume and cortical surface area). This finding may be biological, or it could relate to data quality or artifacts, and we therefore examined the characteristics of the cohorts for potential explanations of the structural distinctions. In terms of motion, whether measured by $FD_{original}$ or $FD_{filtered,4\text{-}TR}$, the burst and deep breath groups are not different than random groups, and the clean group exhibits very little motion. Motion thus does not account for these structural distinctions in any obvious way. As might be expected, variance in RVT, RV, and ENV strongly distinguishes burst from single breath and clean groups: burst scans have among the highest respiratory variance, whereas single deep breath and clean scans have among the lowest (because they lack bursts). Additionally, the deep breath and clean groups have among the highest mean respiratory measure values (because they lack the tapers of bursts). Distinctions between groups in respiratory measures thus match the structural distinctions. Given that structural brain features are known to relate to age, body size, and sex, these variables were also examined. Age was not different between groups, nor was BMI. The burst group was slightly taller than the other groups but not significantly so, and the burst group was heavier than the other groups ($p = 0.04$ compared to deep breath and $p = 0.06$ compared to clean groups by two-sample t-test). Eliminating a single subject from each group (a heavy one from the burst group and light ones from the deep breath and clean groups) eliminated these between-group differences from even trend-level significance, while preserving all of the distinctions noted above when the permutation tests were re-run with the 20-subject groups. Additionally, the re-run groups did not differ in age, height, weight, or BMI from random groups at even trend-level significance, though the deep breath and clean groups were in the 5-10th percentiles for weight and height.

However, no amount of post-hoc balancing could rectify the striking disparity in sex composition of the groups: the burst group was 2/3 male whereas the deep breath and clean groups were both 3/4 female. Many of the associations of variables above could be attributed to sex effects. It is well-established that males have brain volumes approximately 10-15% larger than women, even after controlling for height (e.g., (7, 8), for extensive review see (9)). Similarly, cortical thickness has repeatedly been found to be thinner in males (10, 11). It is also well-established that males drink more alcohol, and drink more problematically, than women (12, 13).

The groups were formed without knowledge of the sex of participants, and it is unlikely that such unbalanced sex compositions would emerge 3 times in random group formation (given the

sex composition of the subjects, the joint probability of selecting an equivalently or more biased sample is p = 3.3e-5).

Supplementary Note 4

Automated breathing indices. Here we present a proof-of-principle algorithm that uses respiratory traces and global fMRI signals (from minimally preprocessed data) as substrates to gauge the likelihood of bursts and deep breaths in scans. As illustrated in Figure 4, this classification algorithm replicates and reinforces the main findings of this paper: i) our 3 groups of investigator-chosen subjects (clean, bursts, deep breaths) are markedly differentiated in terms of algorithmic respiratory events, ii) our investigator ratings of total bursts and deep breaths in subjects agree well with the algorithmic indices, and iii) significant sex differences in bursts but not deep breaths are algorithmically detected. Individual scans will be illustrated to show how the algorithm performs in unambiguous instances of respiratory patterns, as well as in more ambiguous and haphazard breathing patterns, thus illustrating the challenges faced by the next generation of algorithms. The templates described below are available for download along with Matlab code implementing the algorithmic approach (https://osf.io/u35f8/).

High-level description. At each timepoint of a scan, the algorithm creates priors (probabilities) for deep breath and burst likelihood from the respiratory traces and then multiplies these priors into the similarity of the global fMRI timeseries to fMRI templates of bursts and deep breaths. Identification of a pattern thus requires simultaneous evidence of the breathing pattern in respiratory traces and in fMRI signals.

Medium-level description: The algorithm 1) searches respiratory traces for unusually long and deep breaths and creates a probability vector for deep breaths, 2) calculates variance in the envelope of respiratory traces as a gauge of burst likelihood and suppresses that variance where deep breaths are likely, 3) calculates a probability vector of bursts from the suppressed envelope of respiratory variance in combination with similarity of respiratory traces to respiratory trace templates of bursts derived in single subjects, 4) slides windowed fMRI templates of bursts and deep breaths along the global fMRI timeseries to create covariance vectors expressing the match of global fMRI timeseries to the respiratory event fMRI templates, 5) multiplies the respiratory-trace-derived probability vectors into the fMRI-signal-derived template-matching vectors, and 6) downweighs the products for both patterns by indicators of haphazard breathing, namely variance in breathing rates and respiratory power at low frequencies.

Fine-grained description: Event template construction, fMRI templates, fMRI covariance vectors: We visually identified 10 subjects with plentiful deep breaths and 10 subjects with plentiful bursts, visually marked the start of events in each subject, and extracted the respiratory traces and global fMRI timeseries surrounding each event. In the 10 deep breath subjects, 6-26 usable events were marked per subject (mean 15, std 6.65, 130 events total), and in the 10 burst subjects, 10-18 usable events were marked per subject (mean 12.3, std 2.7, 115 events total). Two authors (JDP, CJL) jointly agreed on event markings, global fMRI signals

surrounding the events were extracted, after which event onsets were permitted to adjust several timepoints forward and backward to maximize cross-correlation of the global fMRI signals, which yielded shifts centered on zero with >95% events shifted less than 5 timepoints (<3.6 sec) and median correlations of event waveforms over 0.8 in both patterns. A small minority of events whose cross-correlation maximized at farther shifts, or whose cross-correlation was under 0.55, were discarded as unrepresentative (8/123 burst events, and 20/150 deep breaths, typically due to severe motion artifacts, not included in totals reported above). All subjects, all retained events, and the mean within-subject global fMRI timeseries (the fMRI templates) are shown in Supplementary Figure 13: fMRI waveforms for deep breaths and bursts are internally consistent and highly correlated within each pattern, and the patterns have low zero-lag correlations across patterns (but substantial lagged correlations, illustrated and addressed below). These fMRI templates were slid along each minimally preprocessed fMRI scan to yield vectors of covariance at each timepoint for both respiratory pattern templates in all scans of all subjects (part 4 of the medium-level description above).

Deep breath identification. Deep breaths tend to be slow and are defined as drawing 2-5x the normal tidal volume in the pulmonary literature. Elsewhere, we have demonstrated in HCP data the slowness of deep breaths and their larger-than-normal amplitude, but also the fact that respiratory traces during fMRI scans are often flawed, corrupted, or haphazard, factors that influence our approach to identification of deep breaths. First, we blurred respiratory traces over 5 seconds and used a peak-finding algorithm with minimum 2-second breath separations to define breath peaks and inter-peak intervals. The median was removed from these breath intervals and breaths lasting > 1.5 sec longer than the median were identified. To help boost near-threshold breaths, breaths lasting > 1.3 sec longer than the median were gently augmented by the envelope of respiration if the envelope was much higher than average at that point (which usually happens in deep breaths). This boosting step helped differentiate bursts from deep breaths because whereas deep breaths usually display increased envelope just as breathing slows, burst breathing slows (i.e., begins to potentially meet deep breath criteria) in the taper at the same time as the envelope usually decreases. Thus, the boost operates almost exclusively to augment identification of deep breaths. Following boosting, breath duration values were multiplied by the height of the peak-finding peak so long as the peak exceeded the median peak value, with attenuation for highly variable breathing rates. Thus, the deep breath probability vector was maximized at times of slowed breathing with larger-than-normal amplitude in the respiratory traces, and explicitly attempted to avoid slowed breathing with lower-than-normal amplitude (characteristic of bursts). Multiplication of this probability vector into the 10 deep breath fMRI template covariance vectors (described above) yielded a final set of 10 product traces indicating likelihood of a deep breath with consequences in fMRI signals, a single example is shown in Supplementary Figure 14. The area under these curves gauges deep breath effects in the scan. Supplementary Figure 15 shows a scan illustrating the interaction of deep breath probability with the fMRI templates: the deep breath near timepoint 800 yields a large product trace and a large area under the curves, whereas the deep breath near timepoint 70 had modest effects in the fMRI signal and thus creates a smaller product. The area under the curve thus can be thought of as the severity of the pattern, encompassing its prevalence and the magnitude of its effects in fMRI signals. This

joint approach is needed because numerous irregularities in the respiratory traces would otherwise produce numerous false positive deep breaths without the extra requirement that the typical deep effect is also seen in fMRI signals (illustrated in Supplementary Figure 16).

Burst probability suppression by deep breaths. Bursts are mainly characterized by changes in the amplitude of breathing while (often) maintaining a relatively steady breathing rate. The envelope of the respiratory trace is therefore a suitable indicator of burst probability. However, deep breaths can also modulate the envelope, with frequency characteristics overlapping those of bursts, precluding spectral separation. We therefore used deep breath probability vectors to suppress identification of bursts, convolving the deep breath probability vector with a gaussian, and using that vector to suppress identification of bursts at or near high-probability deep breaths. Supplementary Figure 14 illustrates the suppression vector as the dotted line over and flanking the deep breath probability vector, seen also in Supplementary Figures 15-17. Supplementary Figure 17 in particular shows a deep breath occurring in the midst of a series of bursts, with proper identification of both the deep breath and the flanking bursts due to the suppression vector. Leading and lagging suppression occurs because envelope changes extend in time due to windowed calculation, and is also used to address the lagging and leading correlation of the burst and deep breath fMRI templates (i.e., a true deep breath will virtually always have preceding and following covariance with the burst fMRI template, see for example the elevations in faded red lines in Supplementary Figure 14 before and after the deep breath). The action of the suppression vector is seen in the third panel, where a maroon trace shows envelope variance after suppression, with a bright red trace underneath it showing the original variance (i.e., when bright red is seen, that variance has been suppressed by deep breath probability).

Burst identification. The 10 subjects providing fMRI templates for bursts also provided respiratory trace templates at the same events, from which the envelope was calculated. Using sliding windows, we obtained the covariance between the template respiratory envelopes and respiratory envelopes of all scans, thus providing a gauge of burst likelihood via respiratory template matching. The envelope had already been suppressed by deep breath likelihood as explained above, and we additionally calculate the windowed variance of the suppressed envelope, which is frequently markedly elevated in bursts. These two vectors – a variance vector and a covariance vector (multiplied by a gaussian with scaling by low-frequency respiratory power, often characteristic of bursts) – were summed to yield the probability vector for bursts. This probability vector was then multiplied into the fMRI template covariance vector for bursts for each of the 10 templates, and the area under the curves served as the gauge of burst severity. Example of pure bursts are seen in Supplementary Figure 18 and 19.

Performance in single scans. Supplementary Figures 14-19 have illustrated successful identification of fairly clear events. To illustrate the algorithm properly assigning no events to a scan, see Supplementary Figure 20 or 21. Our development of the algorithm focused on successful identification of clear events and avoiding identification of events when clear events were absent. In these situations, the algorithm performs well. However, in subjects with more chaotic breathing patterns, the respiratory traces can include exceptionally long pauses, huge

inspirations, or haphazard rates, all of which challenge the template-based and outlier-based approach of the algorithm. Supplementary Figures 22 and 23 illustrate such scans. Readers may view all scans of all subjects in an online movie.

Haphazard scans will add noise to group-level analyses on the classifier's results. Rather than judging scans and excluding exceptionally haphazard ones, we identified two markers of haphazard scans – variability in breathing duration (the dotted red trace in the third panels) and very low frequency power near 0.03 Hz in respiratory trace – to yield a subject score of haphazardness. Areas under the curves were divided by this score to attenuate the influences of these difficult-to-assess scans. Note that this score has no relation to fMRI signals or scan properties, it is only derived from the respiratory belt (meaning that, for example, potential sex differences in scan properties due to head size, or motion-related scan qualities, cannot bias this measure). Also note that this measure has no inherent relation to the area under the curve scores for deep breaths or bursts, and that it is applied identically to product scores of both breathing patterns. This correction is applied at the group level (but corrections applied per-scan vs. per-subject yield the same result). We call the final result an algorithm index to help distinguish it from human rater scores.

Comment on implementation and release. Our visual presentation of the algorithm here utilizes gray plots with many traces and the full fMRI timeseries illustrated, but all that is actually needed for the algorithm to run are respiratory traces, respiratory templates, fMRI templates, and global fMRI signals for a dataset. Templates are included in the online materials of this paper, as is code, and deriving the global fMRI signal in a dataset is a commonplace maneuver. Thus, anyone may implement, and modify, this routine.

Algorithmic classifier performance. Beyond performance in single scans, three claims of the paper are evaluated: 1) Do the rater scores scale with algorithm indices, 2) Do the clean, deep breath, and burst groups index appropriately, and 3) Are sex differences present in burst but not deep breath indices? Answers in the affirmative on all points are illustrated in Figure 4 of the main text. Rater scores of total bursts over 399 subjects (1,596 scans) were correlated, separately in males and females, with algorithmic indices at $r = 0.57$ ($p < 1e-20$) and 0.60 ($p < 1e-20$). For deep breaths, in males and females, correlations were $r = 0.62$ ($p < 1e-20$) and 0.53 ($p < 1e-20$). Thus, our visual ratings correspond to the algorithmic indices. As shown in Figure 4, the clean, deep breath, and burst groups were highly dissimilar in algorithmic indices, with exceedingly low indices in the clean group on both breathing patterns, high indices of deep breaths and not bursts in the deep breath group, and high indices of bursts but not deep breaths in the burst group. ANOVA yielded effects in each breathing pattern index across groups, and post-hoc t-tests were significant. In terms of sex differences, rater scores of breathing patterns over 399 subjects are plotted in Figure 4 next to the algorithmic indices. Both indicate a sex differences in bursts, which are more common in men, and a lack of sex differences in deep breaths.

Summation. The presented algorithmic identification of bursts and deep breaths combines information in respiratory traces and global fMRI timeseries to create indices of the severity of

each pattern in a scan. Reasonably clear examples of burst and deep breaths are usually well-identified in time, and, across 399 subjects, visual ratings made prior to the algorithm scaled with algorithm indices. The algorithm affirmed respiratory pattern properties of the 3 groups used to initially detect sex differences and reaffirmed the general finding of sex differences in bursts over hundreds of subjects. Collectively, these results give firm support to the major findings of the paper, and also demonstrate a proof-of-principle automated breathing pattern identifier that works in a large majority of examined scans. Haphazard and unusual breathing patterns challenge the automated approach (and human raters) and represent a target for the next generation of techniques for identifying breathing patterns.

Supplementary Discussion

On periodic breathing. Periodic breathing has been intensely studied because it contributes to sleep disordered breathing (SDB), a medically deleterious condition characterized by periods of hypoxia and subsequent arousal caused by either obstructive or central sleep apnea. SDB is associated with risk for stroke, hypertension, heart failure, ischemic heart disease, premature death, and depression (14). Many major studies of SDB have been conducted in tens of thousands of patients via polysomnography, and the apnea-hypopnea index (AHI) is the cornerstone of defining severity. For example, a recent major study used a 4-tier system classifying sleep disordered breathing as absent, mild, moderate, or severe, according to whether patients had <5, 5-15, 15-30, or >30 events per hour, where events were apnea (>90% drop in airflow from baseline for >10 sec) or hypopnea (>30% drop in airflow from baseline for >10 sec with either arousal or >3% oxygen desaturation) (15). We cannot employ these definitions in the HCP data, but our approach of scoring (from 0-4) the number of runs displaying bursts over an hour's scanning broadly mirrors this approach. Based on comparison to images in the literature, it is likely that many of the bursts would qualify as hypopnea or apnea, though we lack airflow and oxygenation records to prove the case.

Periodic breathing is typically studied during sleep and in clinical populations. Of special interest to understanding bursts during MRI scans at rest in our healthy HCP subjects are 1) investigations of PB in the general, non-clinical population, and 2) attempts to link daytime PB to nighttime PB. In the most recent large study of SDB in the general population (15), among 2,121 residents of Lausanne, Switzerland aged 49-69 with mean BMI of 26 who underwent polysomnography, 84% of males and 61% of females had AHI scores of >5 per hour, and AHI scores were 2-3 times higher in men than in women at all ages studied. Further, AHI was associated with depression, diabetes, hypertension, and metabolic syndrome. These findings resemble those of a 2010 polysomnography study of 1,042 Brazilian subjects selected to statistically represent the adult population of Sao Paulo, where male sex and increased age were strong predictors of increased AHI scores, effects that extended down to age ranges of 20-29 years old and to subjects with BMI <25 (16). Other studies in the general population have also yielded robust effects of sex and age on AHI scores (17, 18), and clinical studies focused on central sleep apneas confirm a strong male bias of such phenomena (19). Of interest to the neuroimaging community, given the cross-sectional association of SDB to depression above (and in other studies), is a longitudinal polysomnography study of 1,408 Wisconsin state

employees reporting a dose-response association of SBD and depression (20). Note that these studies do not generally distinguish between AHI due to central versus obstructive causes of hypopnea or apnea (also note that from the point of view of fMRI imaging correlates of burst phenomena, it is not clear that it would strongly matter whether hypopnea is central or obstructive, since both should modify cerebral blood flow and create the potential for repeated hypoxia).

There is thus a strong precedent in the general population for males to exhibit increased hypopnea and apnea relative to females during sleep, consistent with the sex bias seen in our burst effect. A related question, given the daytime eyes-open fMRI recordings in the HCP dataset, is how PB during sleep relates to PB during daytime wakefulness, a topic that has only recently started to receive attention and only in clinical populations. Daytime PB in heart failure patients is prevalent: in 380 consecutive heart failure patients who underwent 10-minute monitoring at rest during the day, daytime PB was present in 145 (40%) (21). Other studies using 24-hour respiratory monitoring find similar prevalence of daytime PB in heart failure (22). PB during the day also predicts PB at night: 20-minute daytime monitoring of 147 heart failure patients revealed PB in 59% of patients, all centrally generated patterns, and in a 53-subject subset of these patients that also underwent polysomnography, daytime PB was a significant predictor of sleeping PB (23). A more recent study of 337 consecutive heart failure patients who underwent 10-min daytime recordings and polysomnography found daytime PB in 158 (47%) of patients, and also found that nighttime AHI was higher in patients with daytime PB, exclusively due to central and not obstructive events. These results broadly support daytime and nighttime PB being part of a mechanistic continuum, at least within heart failure patients.

PB can be unmasked in healthy adults under particular conditions. Under normoxic conditions, typical adults breathe eupnically while awake, with slightly reduced but highly regular tidal volume and regularity during non-rapid-eye-movement (NREM) sleep, and with markedly more irregular breathing patterns, both in terms of rate and depth, during rapid-eye-movement (REM) sleep (24). In the same healthy subjects, under hypoxic conditions (e.g., with inhaled gases in a laboratory), PB emerges during NREM sleep but not during REM or wakefulness. PB can be provoked during wakefulness in healthy subjects by vigorous exercise under hypoxic conditions (25), and is seen during NREM sleep at high altitude in healthy subjects, with a sex bias (26). Similarly, PB is seen in heart failure patients prominently during NREM sleep but also during wakeful exercise and during daytime recordings. PB during sleep is associated with large, accompanying periodic oscillations in cerebral blood flow.

On periodic breathing mechanisms. Three distinct control systems govern breathing: voluntary control (not discussed further), an automatic-metabolic feedback loop governed primarily by pCO_2 (and pH), and the state of wakefulness (27). The relevant neural circuitry is complicated, but, schematically, a central respiratory pacemaker exists in the medulla, which is modulated by respiratory centers in the medulla and pons that are sensitive to pH and pCO_2, which act to increase ventilation when pCO_2 rises and/or pH drops (CO_2 combines with water to form bicarbonate, a weak acid and a principal buffer of the blood). Wakefulness contributes respiratory drive via suprapontine input, driven largely by activity in the reticular activating

system, activity that is in turn dependent on noradrenergic, serotonergic, and cholinergic nuclei in the brainstem. During wakefulness and REM sleep, these centers contribute respiratory drive via suprapontine input, but this input is greatly diminished during NREM sleep.

Mechanistically, PB is believed to reflect instability in the chemoreflex feedback loop controlling ventilation, such that (excessive) ventilation pushes pCO_2 below a certain apneic threshold, causing respiratory drive to fade or cease, after which pCO_2 rises and eventually passes the threshold, triggering resumption of (excessive) ventilation. PB with varying characteristics (e.g., cycle times, hypopnea vs apnea, crescendo and decrescendo characteristics) can result from modifying the gain of the chemoreceptors, the apneic threshold, external inputs to either of those factors, and the delay between altering pCO_2 at the lungs and detecting pCO_2 centrally (28). For example, the prevalence of PB in heart failure is believed, among multiple mechanisms, to stem from the fact that blood takes an unusually long amount of time to circulate from the lungs to the brain (where pCO_2 is centrally sensed) due to low cardiac output. Stated differently, in heart failure, centrally-guided respiratory efforts respond to centrally-detected pCO_2 at a substantial lag from the actual pCO_2 levels at the lungs, causing respiratory drive inappropriate to and out of sync with the actual gas tensions of blood at the lungs.

The prevalence of PB in NREM sleep, relative to wakefulness and REM sleep, is believed to result from the loss of the wakefulness respiratory drive, which leaves ventilation governed nearly entirely by the pCO_2 feedback loops. A critical parameter for determining whether PB occurs is not the absolute value of pCO_2, but rather whether the apneic threshold for pCO_2 – the threshold below which rhythmic breathing ceases – is numerically close to the eupneic pCO_2, referring to steady-state pCO_2. If those thresholds are close, then relatively small increases in ventilation can drive pCO_2 below the threshold, initiating apnea and the periodic cycle. Experimental values of such parameters in young adult volunteers are eupneic pCO_2 of 45 mm Hg during wakefulness, of 48 mm Hg during NREM sleep, and apnea thresholds of 43 mm Hg (29). One reason PB is less common in females than males is that pre-menopausal women have lower apneic thresholds, meaning that larger ventilatory perturbations from eupnea are needed to drop below the apneic pCO_2 threshold (29). This sex difference in apneic threshold appears to be related to the balance of androgens and estrogens, for administering testosterone to premenopausal women elevates the apneic threshold without altering eupneic pCO_2 (30). Relatedly, SDB becomes markedly more prevalent and sex-balanced at post-menopausal ages (15), obese hypogonadal males appear relatively unlikely to develop SDB (31), and weight-matched females with androgen-producing tumors appear unusually susceptible to SDB (32).

A key element to the propagation of periodic breathing is that resumption of breathing overcorrects desired gas tensions. Such overshoots can arise from multiple factors, such as delays in central chemoreception of pCO_2, or excessive gain on respiratory drive, but they can also occur due to state changes. For example, during NREM sleep, some hypoxic/hypercarbic periods end in arousals, briefly restoring awake set-points for pCO_2, resulting in strong, transient ventilatory efforts due to wakefulness drive that rapidly bring pCO_2 under the apneic threshold, which, as the subject falls back into NREM sleep, and wakefulness drive fades, then

causes apnea to resume. Not all cycles of PB during sleep demonstrate arousal on EEG, and, since PB occurs during wakeful exercise, such cycles of arousal are not necessary for PB to occur, but much PB during NREM sleep likely exhibits transient arousals timed to increases of ventilation (33). Such intermittent arousals may account for the variable association of heart rate with burst onsets in the HCP data.

Comparison of burst records to periodic breathing records in the literature (see Figure 7). PB demonstrates different properties depending on the context in which it occurs. PB occurs in the first few weeks of life in infants, and in healthy adults in hypoxic conditions (e.g., with inhaled gases or at high altitude). The most common clinical context is heart failure, where PB is found in large fractions of patients. PB is also prevalent among patients with strokes, and in patients on high doses of opioids, but PB has different properties in these settings.

The circulation time of blood is strongly linked to the duration of the hyperpneic (ascending) arms of PB but not length of the apneic (descending) arms, which largely explains the exceptionally long cycles of PB seen in heart failure (on average, 60 seconds) (34). In stroke patients, those without left ventricular dysfunction (i.e., heart failure) displayed PB cycles on average 20 seconds shorter than those with left ventricular dysfunction, changes attributable to the hyperpneic but not apneic portions of PB (35). Thus, in subjects with normal heart function, one would expect asymmetric PB waveforms with steep, brief ascending portions, which is the form generally seen in bursts in HCP subjects.

PB is common in patients with strokes. Although classically described as occurring due to massive bilateral lesions, more recent, larger studies controlling for cardiac factors find no effect of lesion size or severity, and obtain prevalence estimates by polysomnography of 20% among 185 patients (36) or 19% of 93 patients (35). The PB is often transient, occurring in the acute period and resolving within months, and the occurrence of PB is more likely in patients with concomitant cardiac dysfunction. PB with cycle times of 25-40 seconds are commonly seen in the absence of marked cardiac dysfunction (an example is shown in Figure 7).

PB can occur with both acute and chronic opioid use, and the severity of hypoxia can be dramatic, with AHI scores far above 60 events per hour (37, 38). Estimates in methadone clinic populations are that 30% of patients exhibit central apnea (39). An example of opioid-induced PB is shown in Figure 7, exhibiting a typical cycle time of 25 seconds. PB can resolve on discontinuation of opioids (40).

The above studies used polysomnography, and in them it is common to find a male sex bias in PB prevalence, a tendency of PB to occur in NREM sleep, and a tendency for greater prevalence in older subjects, consistent with effects noted previously.

To aid recognition of PB, Figure 7 plots instances of each of these kinds of PB from the literature, as well as several instances of bursts in HCP subjects. In comparison to bursts, the published PB are much more regular in their periodicity. The PB illustrations are from polysomnography when patients were asleep, fully exposing the mechanisms of the automatic-

metabolic loop. By contrast, in the HCP data there is no firm evidence that subjects are sleeping and there is likely (fluctuating) wakefulness drive influencing ventilation, which we suspect leads to a less regular form of the chemoreflex waxing/waning cycles. Cycle times in HCP subjects can be long, approaching a minute, but more typically they are closer to 30 second cycles.

Supplementary References

1. J. D. Power, C. J. Lynch, A. W. Gilmore, S. J. Gotts, A. Martin, Reply to Spreng et al.: Multiecho fMRI denoising does not remove global motion-associated respiratory signals. *Proc Natl Acad Sci U S A* **116**, 19243-19244 (2019).

2. J. D. Power *et al.*, Characteristics of respiratory measures in young adults scanned at rest, including systematic changes and "missed" deep breaths. *Neuroimage* 10.1016/j.neuroimage.2019.116234, 116234 (2019).

3. J. D. Power, M. Plitt, T. O. Laumann, A. Martin, Sources and implications of whole-brain fMRI signals in humans. *Neuroimage* **146**, 609-625 (2017).

4. J. D. Power, A simple but useful way to assess fMRI scan qualities. *Neuroimage* **154**, 150-158 (2017).

5. J. D. Power *et al.*, Ridding fMRI data of motion-related influences: Removal of signals with distinct spatial and physical bases in multiecho data. *Proc Natl Acad Sci U S A* **115**, E2105-E2114 (2018).

6. K. A. Franklin, E. Sandstrom, G. Johansson, E. M. Balfors, Hemodynamics, cerebral circulation, and oxygen saturation in Cheyne-Stokes respiration. *J Appl Physiol (1985)* **83**, 1184-1191 (1997).

7. E. Luders, H. Steinmetz, L. Jancke, Brain size and grey matter volume in the healthy human brain. *Neuroreport* **13**, 2371-2374 (2002).

8. C. M. Leonard *et al.*, Size matters: cerebral volume influences sex differences in neuroanatomy. *Cereb Cortex* **18**, 2920-2931 (2008).

9. M. Peters *et al.*, Unsolved problems in comparing brain sizes in Homo sapiens. *Brain Cogn* **37**, 254-285 (1998).

10. E. Luders *et al.*, Gender effects on cortical thickness and the influence of scaling. *Hum Brain Mapp* **27**, 314-324 (2006).

11. E. R. Sowell *et al.*, Sex differences in cortical thickness mapped in 176 healthy individuals between 7 and 87 years of age. *Cereb Cortex* **17**, 1550-1560 (2007).

12. R. W. Wilsnack *et al.*, Gender differences in alcohol consumption and adverse drinking consequences: cross-cultural patterns. *Addiction* **95**, 251-265 (2000).

13. R. W. Wilsnack, S. C. Wilsnack, A. F. Kristjanson, N. D. Vogeltanz-Holm, G. Gmel, Gender and alcohol consumption: patterns from the multinational GENACIS project. *Addiction* **104**, 1487-1500 (2009).

14. W. Randerath *et al.*, Definition, discrimination, diagnosis and treatment of central breathing disturbances during sleep. *Eur Respir J* **49** (2017).

15. R. Heinzer *et al.*, Prevalence of sleep-disordered breathing in the general population: the HypnoLaus study. *Lancet Respir Med* **3**, 310-318 (2015).

16. S. Tufik, R. Santos-Silva, J. A. Taddei, L. R. Bittencourt, Obstructive sleep apnea syndrome in the Sao Paulo Epidemiologic Sleep Study. *Sleep Med* **11**, 441-446 (2010).

17. J. Duran, S. Esnaola, R. Rubio, A. Iztueta, Obstructive sleep apnea-hypopnea and related clinical features in a population-based sample of subjects aged 30 to 70 yr. *Am J Respir Crit Care Med* **163**, 685-689 (2001).

18. T. Young *et al.*, The occurrence of sleep-disordered breathing among middle-aged adults. *N Engl J Med* **328**, 1230-1235 (1993).

19. D. D. Sin *et al.*, Risk factors for central and obstructive sleep apnea in 450 men and women with congestive heart failure. *Am J Respir Crit Care Med* **160**, 1101-1106 (1999).

20. P. E. Peppard, M. Szklo-Coxe, K. M. Hla, T. Young, Longitudinal association of sleep-related breathing disorder and depression. *Arch Intern Med* **166**, 1709-1715 (2006).

21. M. T. La Rovere *et al.*, Clinical relevance of short-term day-time breathing disorders in chronic heart failure patients. *Eur J Heart Fail* **9**, 949-954 (2007).

22. T. Brack *et al.*, Daytime Cheyne-Stokes respiration in ambulatory patients with severe congestive heart failure is associated with increased mortality. *Chest* **132**, 1463-1471 (2007).

23. R. Poletti *et al.*, Risk factors and prognostic value of daytime Cheyne-Stokes respiration in chronic heart failure patients. *Int J Cardiol* **137**, 47-53 (2009).

24. A. Berssenbrugge, J. Dempsey, C. Iber, J. Skatrud, P. Wilson, Mechanisms of hypoxia-induced periodic breathing during sleep in humans. *J Physiol* **343**, 507-524 (1983).

25. E. Hermand, A. Pichon, F. J. Lhuissier, J. P. Richalet, Periodic breathing in healthy humans at exercise in hypoxia. *J Appl Physiol (1985)* **118**, 115-123 (2015).

26. P. N. Ainslie, S. J. Lucas, K. R. Burgess, Breathing and sleep at high altitude. *Respir Physiol Neurobiol* **188**, 233-256 (2013).

27. S. Javaheri *et al.*, Sleep Apnea: Types, Mechanisms, and Clinical Cardiovascular Consequences. *J Am Coll Cardiol* **69**, 841-858 (2017).

28. M. C. Khoo, Determinants of ventilatory instability and variability. *Respir Physiol* **122**, 167-182 (2000).

29. X. S. Zhou, S. Shahabuddin, B. R. Zahn, M. A. Babcock, M. S. Badr, Effect of gender on the development of hypocapnic apnea/hypopnea during NREM sleep. *J Appl Physiol (1985)* **89**, 192-199 (2000).

30. X. S. Zhou, J. A. Rowley, F. Demirovic, M. P. Diamond, M. S. Badr, Effect of testosterone on the apneic threshold in women during NREM sleep. *J Appl Physiol (1985)* **94**, 101-107 (2003).

31. E. Harman, J. W. Wynne, A. J. Block, L. Malloy-Fisher, Sleep-disordered breathing and oxygen desaturation in obese patients. *Chest* **79**, 256-260 (1981).

32. R. B. Fogel *et al.*, Increased prevalence of obstructive sleep apnea syndrome in obese women with polycystic ovary syndrome. *J Clin Endocrinol Metab* **86**, 1175-1180 (2001).

33. G. D. Pinna *et al.*, Sleep-wake fluctuations and respiratory events during Cheyne-Stokes respiration in patients with heart failure. *J Sleep Res* **23**, 347-357 (2014).

34. M. J. Hall *et al.*, Cycle length of periodic breathing in patients with and without heart failure. *Am J Respir Crit Care Med* **154**, 376-381 (1996).

35. C. Nopmaneejumruslers, Y. Kaneko, V. Hajek, V. Zivanovic, T. D. Bradley, Cheyne-Stokes respiration in stroke: relationship to hypocapnia and occult cardiac dysfunction. *Am J Respir Crit Care Med* **171**, 1048-1052 (2005).

36. Y. Kim, S. Kim, D. R. Ryu, S. Y. Lee, K. B. Im, Factors Associated with Cheyne-Stokes Respiration in Acute Ischemic Stroke. *J Clin Neurol* **14**, 542-548 (2018).

37. M. P. Tovar-Torres, C. Bodkin, N. L. Sigua, A 44-year-old woman with excessive sleepiness. Opioid-induced central sleep apnea. *Chest* **146**, e204-e207 (2014).

38. M. Mogri, M. I. A. Khan, B. J. B. Grant, M. J. Mador, Central sleep apnea induced by acute ingestion of opioids. *Chest* **133**, 1484-1488 (2008).

39. D. Wang *et al.*, Central sleep apnea in stable methadone maintenance treatment patients. *Chest* **128**, 1348-1356 (2005).

40. M. J. Davis, M. Livingston, S. M. Scharf, Reversal of central sleep apnea following discontinuation of opioids. *J Clin Sleep Med* **8**, 579-580 (2012).

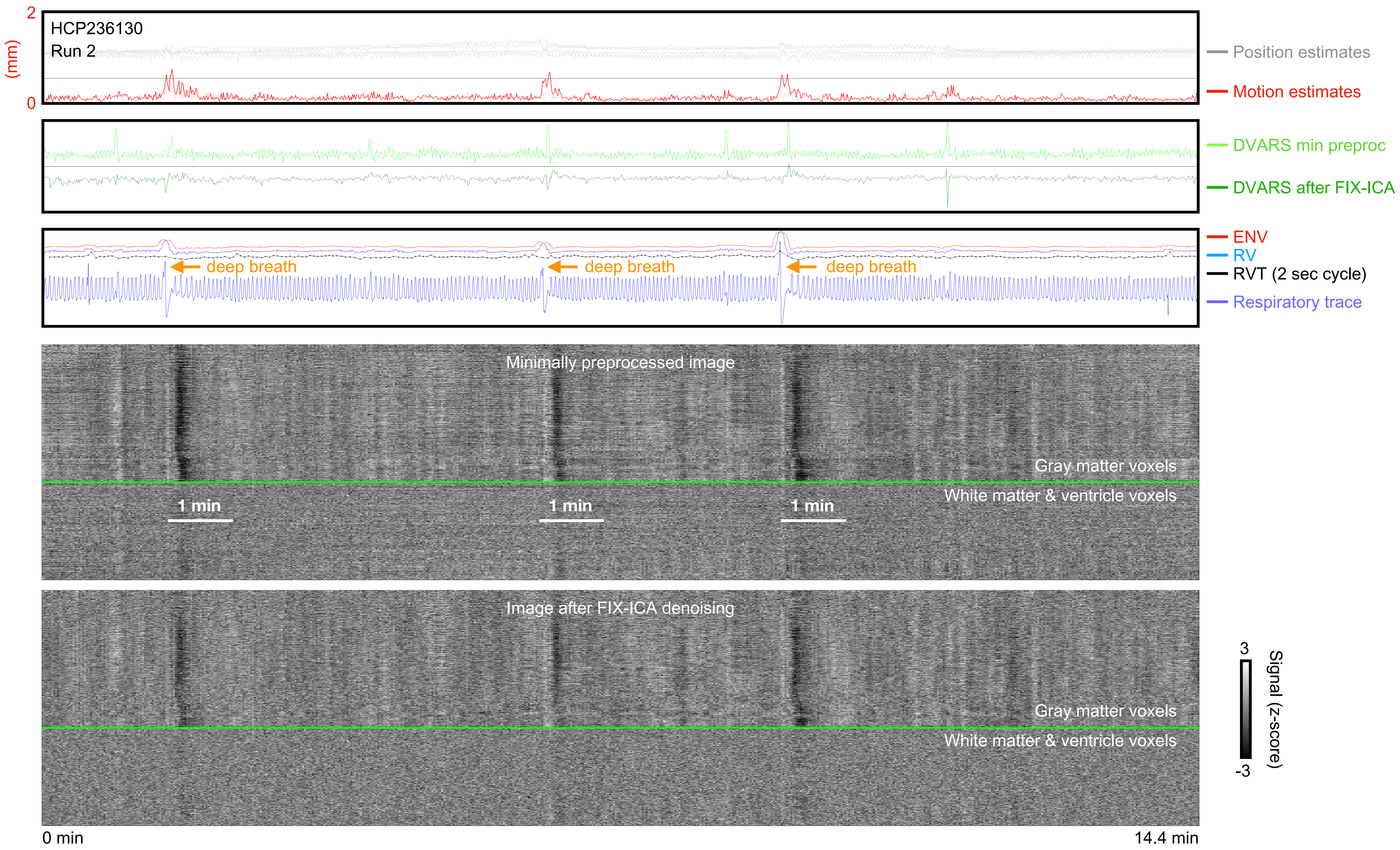

Supplementary Figure 1: Example of deep breaths in a gray plot. This image shows data from a single resting state scan. In the first panel, in gray, are realignment parameters indicating head position, and in red, a framewise displacement (FD) measure of head motion derived from the position traces (calculated over 2.88 sec in bright red, and over 720 ms in light red). In the second panel, in bright and dark green are DVARS values in minimally preprocessed (i.e., undenoised) and FIX-ICA-denoised data, respectively. The third panel shows the respiratory belt trace in blue, and the 3 derived respiratory measures ENV, RV, and RVT. The bottom two panels show heat maps of all in-brain voxel signals organized by anatomical compartment, with gray matter above the bright green lines and white matter (and ventricles) below the lines. Signals before and after FIX-ICA are shown, which are what the DVARS traces above are derived from. Bright orange arrows indicate 3 deep breaths, which all display head motion, DVARS abnormalities, and gray matter signal changes lasting ~25 seconds.

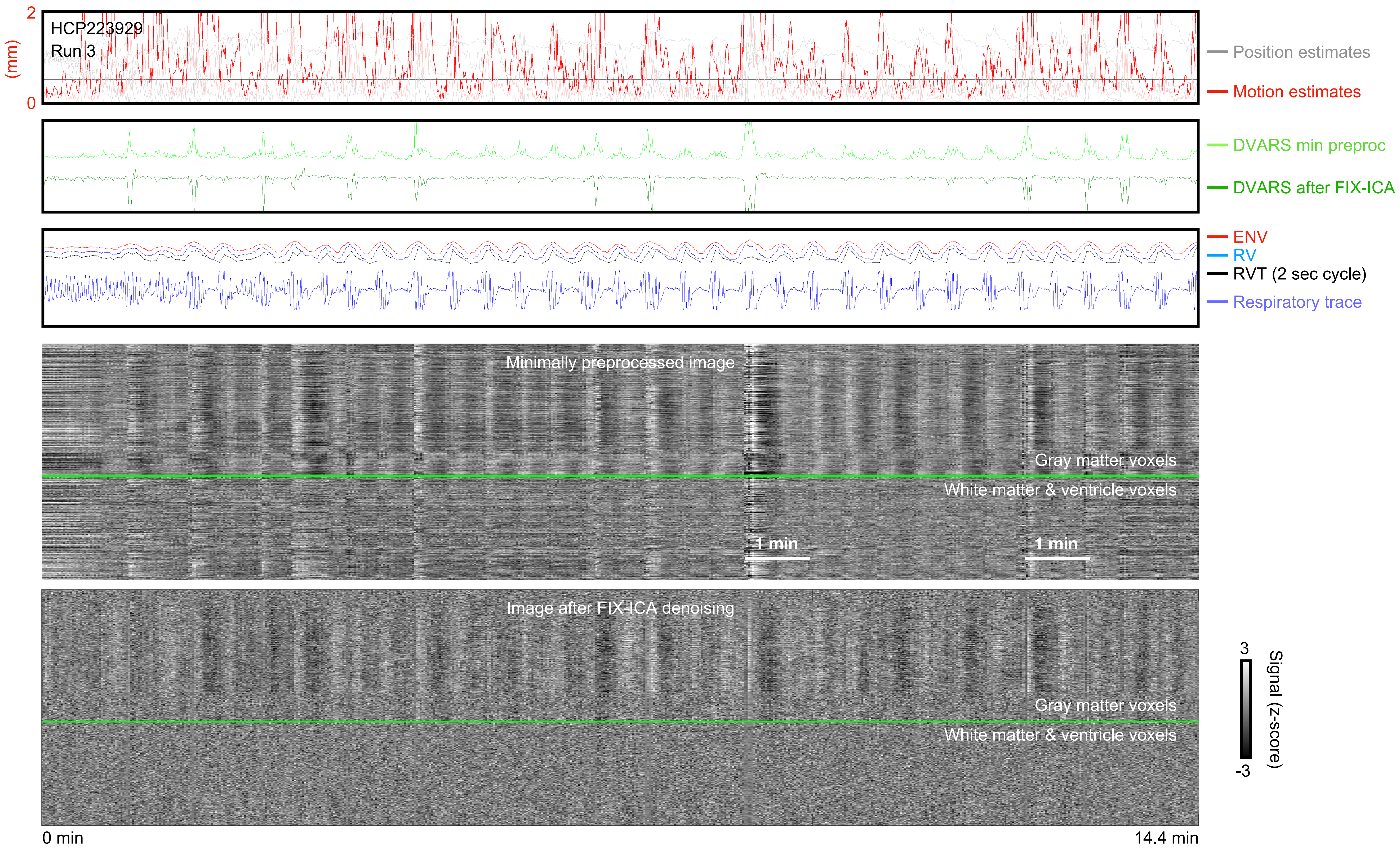

Supplementary Figure 2: Example of bursts in a gray plot. Data in the style of Supplementary Figure 1 is shown, but for a different scan. See Supplementary Movie 1 for such plots of all 1,760 scans entering this report.

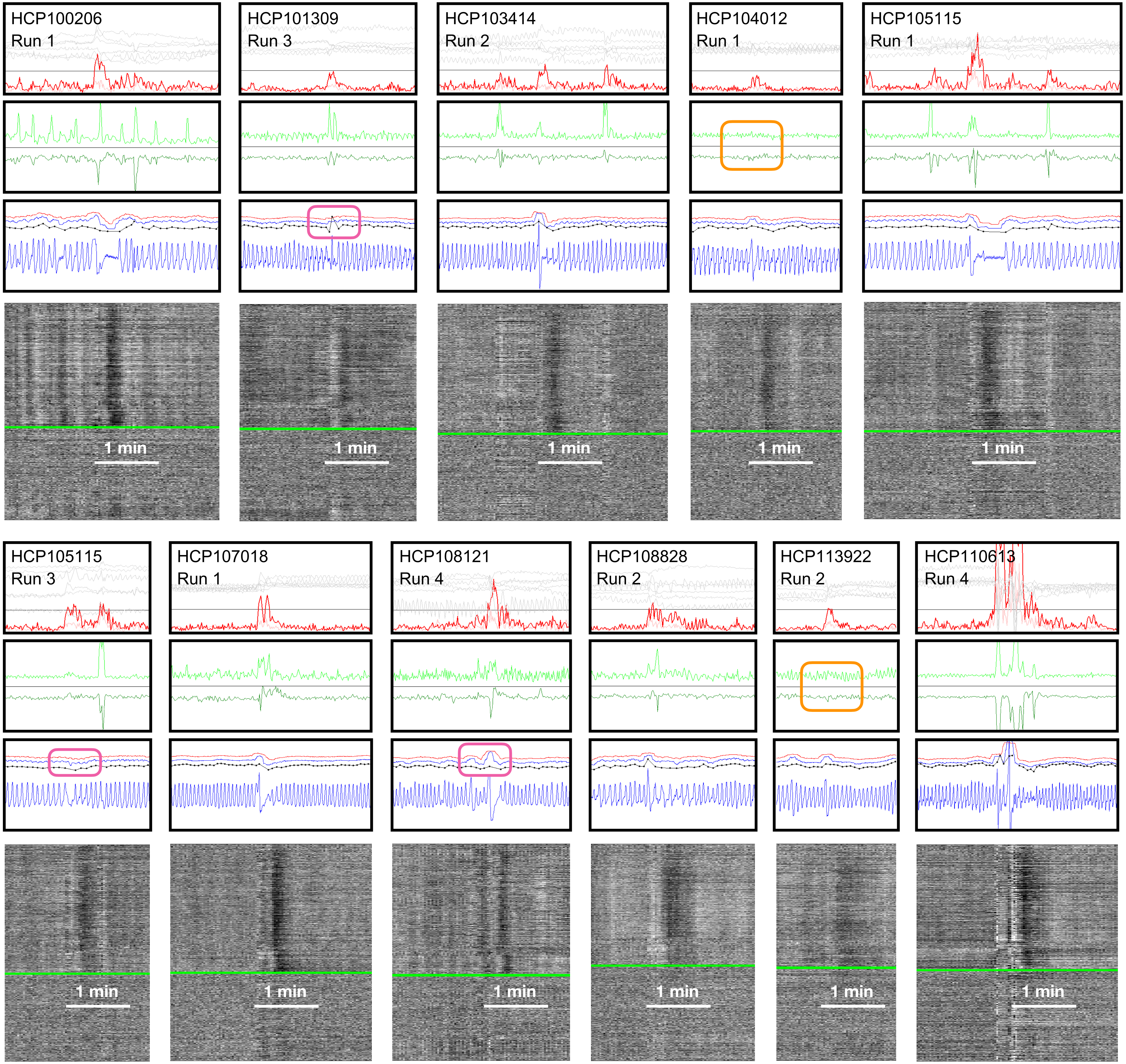

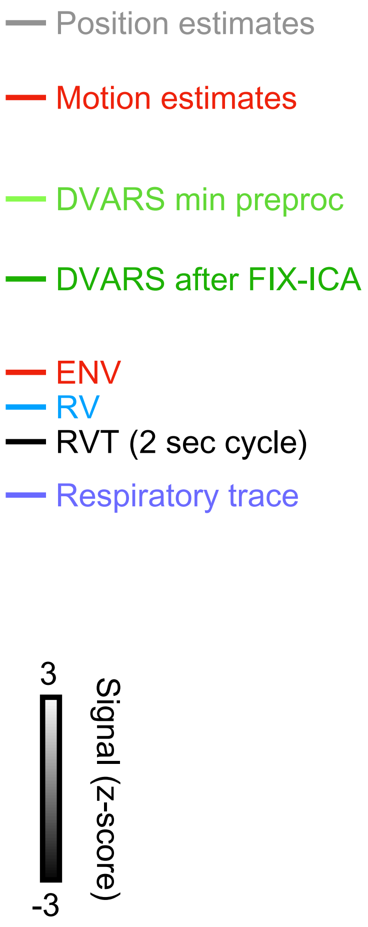

Supplementary Figure 3: Examples of single deep breaths, paralleling Figure 2. For 11 scans with relatively isolated deep breaths (see blue respiratory traces), excerpts of figures in the style of Supplementary Figure 1 are shown. Note that in these instances, transient head motion always accompanies the deep breath. Often, but not always (orange boxes), DVARS spikes and “dips” (after FIX-ICA denoising) accompany the breaths. And often, but not always (magenta boxes), one or more respiratory measures indicate an abnormality at the time of the deep breath. The gray plots are of minimally preprocessed data.

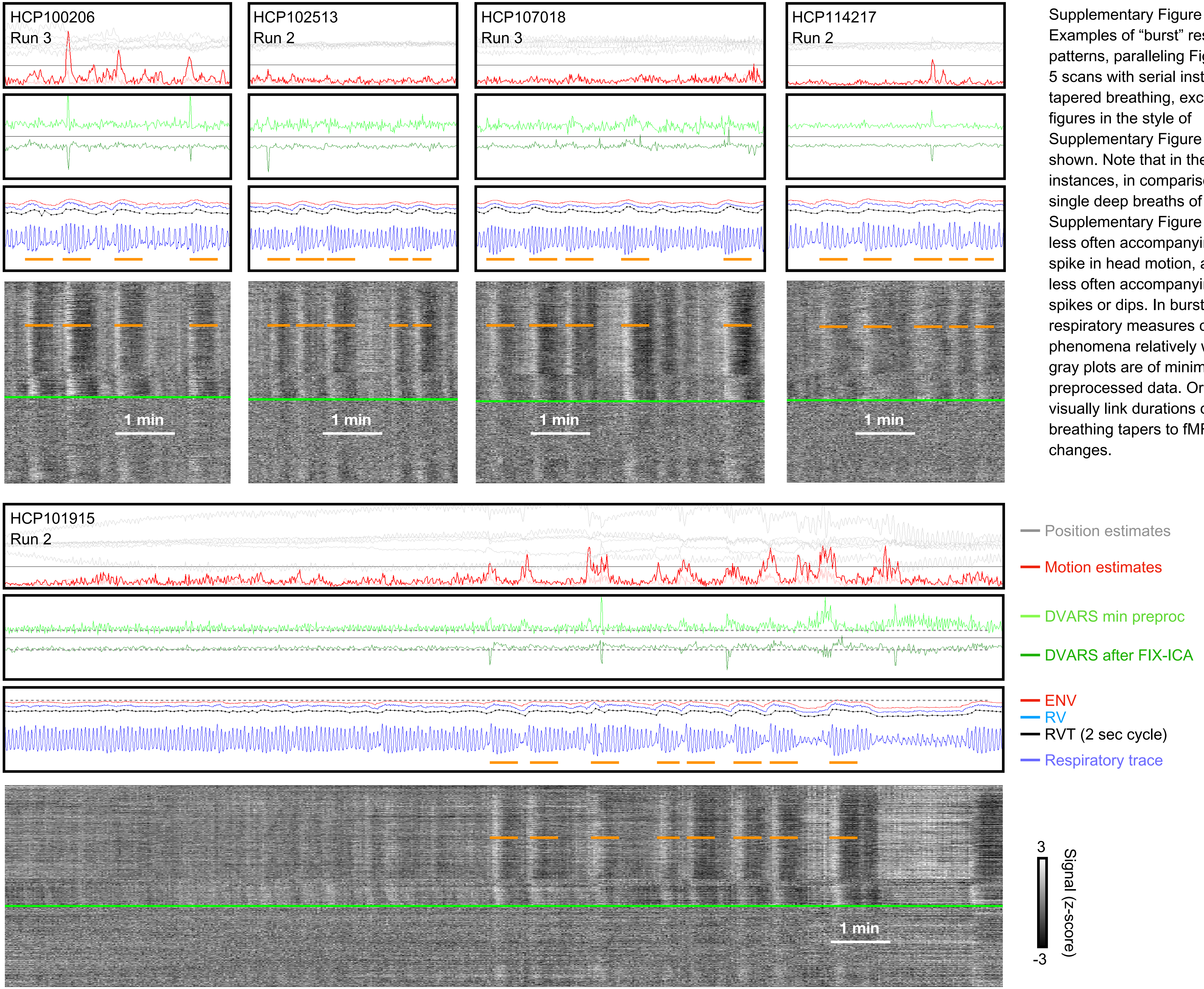

Supplementary Figure 4: Examples of "burst" respiratory patterns, paralleling Figure 2. For 5 scans with serial instances of tapered breathing, excerpts of figures in the style of Supplementary Figure 1 are shown. Note that in these instances, in comparison to the single deep breaths of Supplementary Figure 3, there is less often accompanying a clear spike in head motion, and also less often accompanying DVARS spikes or dips. In burst patterns, respiratory measures capture the phenomena relatively well. The gray plots are of minimally preprocessed data. Orange bars visually link durations of breathing tapers to fMRI signal changes.

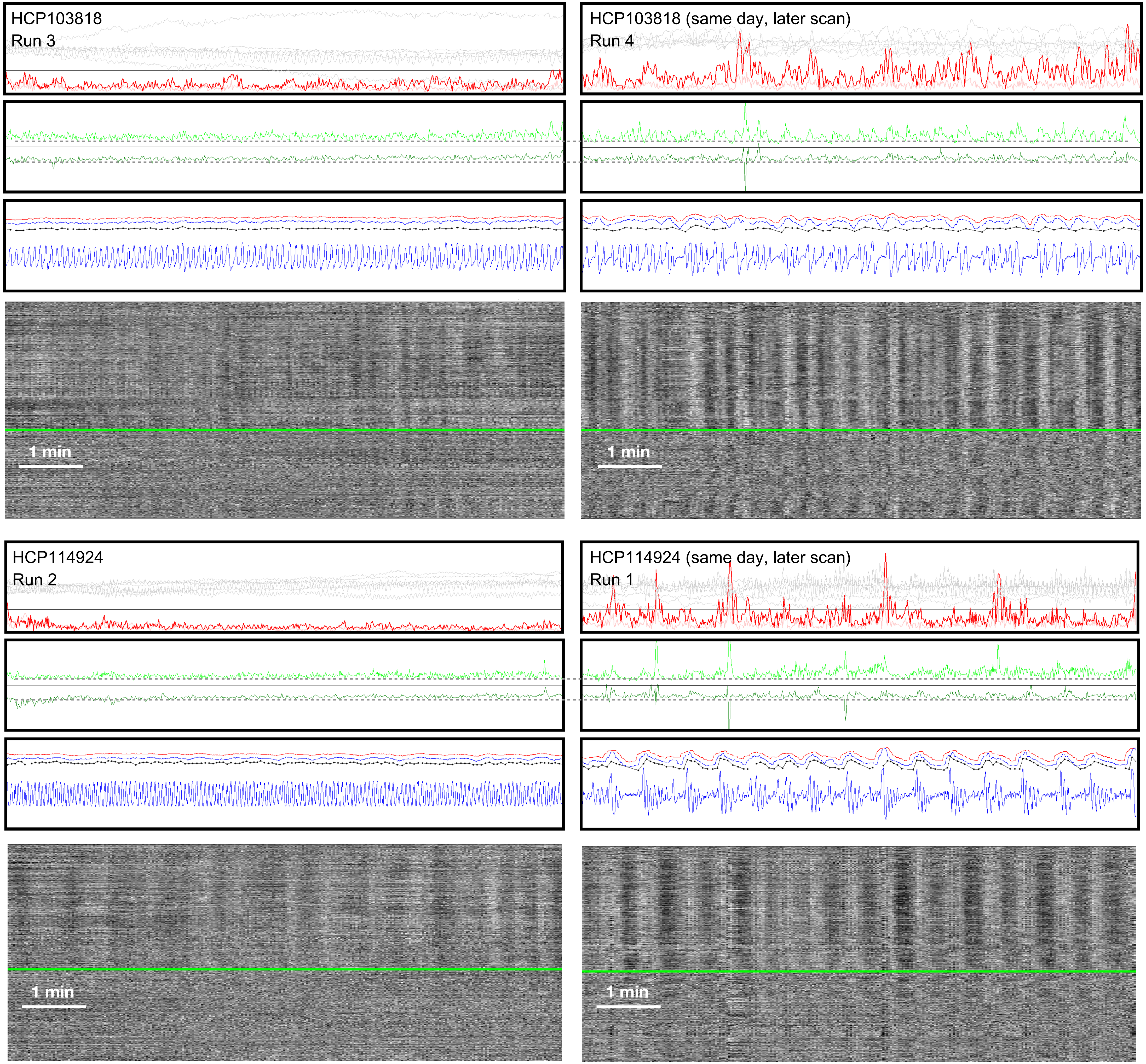

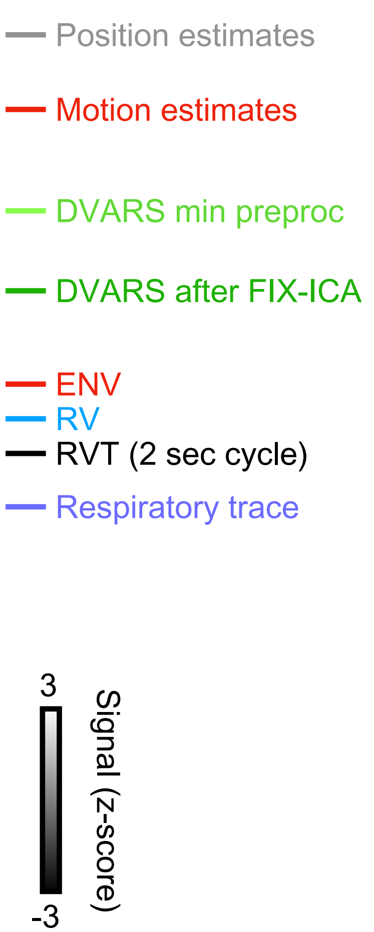

Supplementary Figure 5: Examples of differential breathing within subjects across scans on the same day. These figures follow the conventions of Supplementary Figure 1. The scans at the right are the “later” scans of the day. Both subjects exhibit eupnea in the first scan, whereas the top subject has irregular rate and many pauses in the later scan, and the bottom subject exhibits bursts.

Supplementary Figure 6: Examples of increased respiratory variability over the progression of a single scan, in two subjects. Figures follow conventions of Supplementary Figure 1.

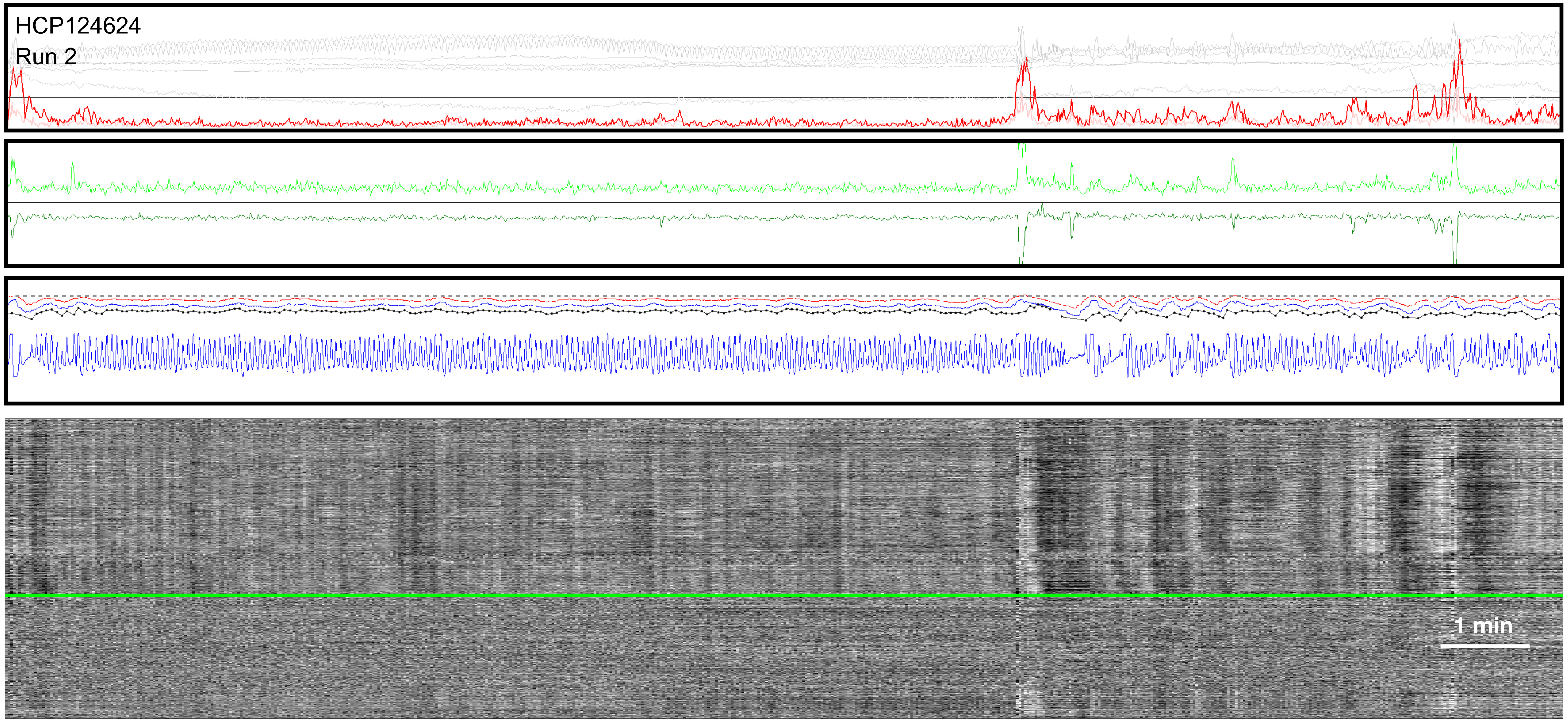

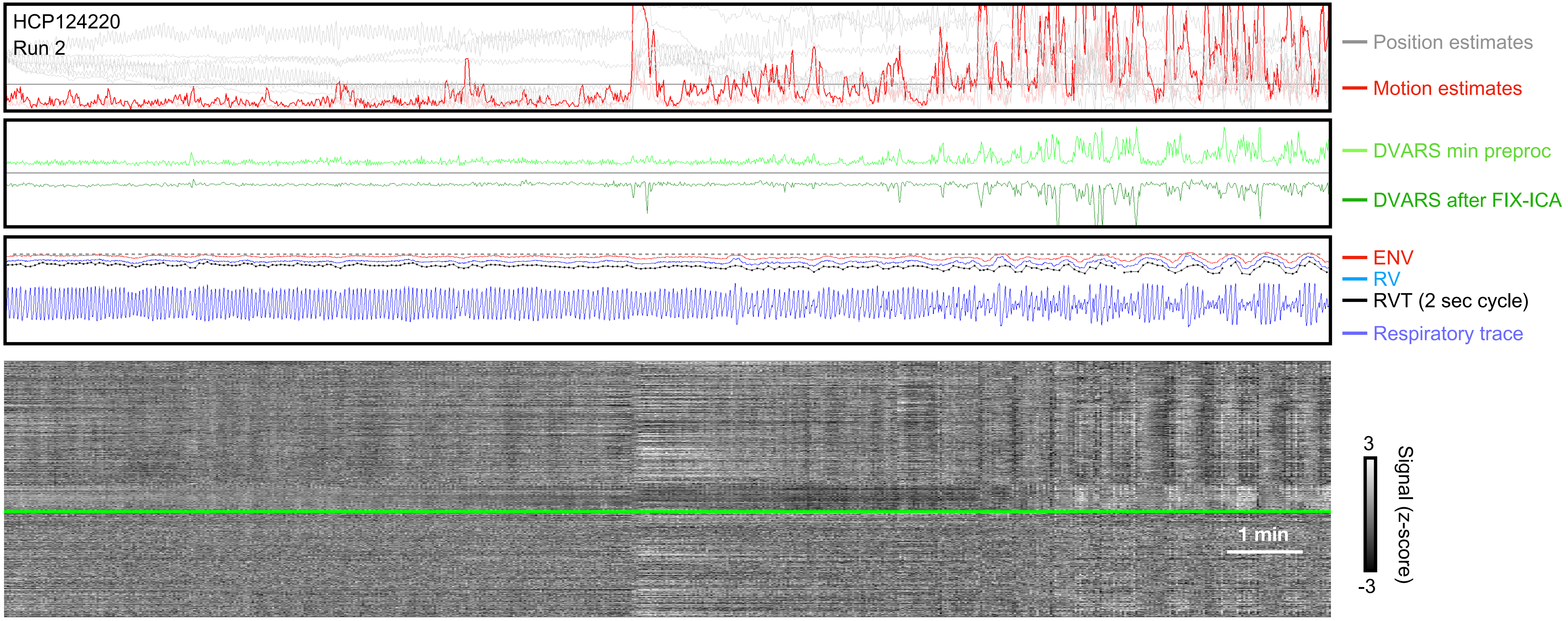

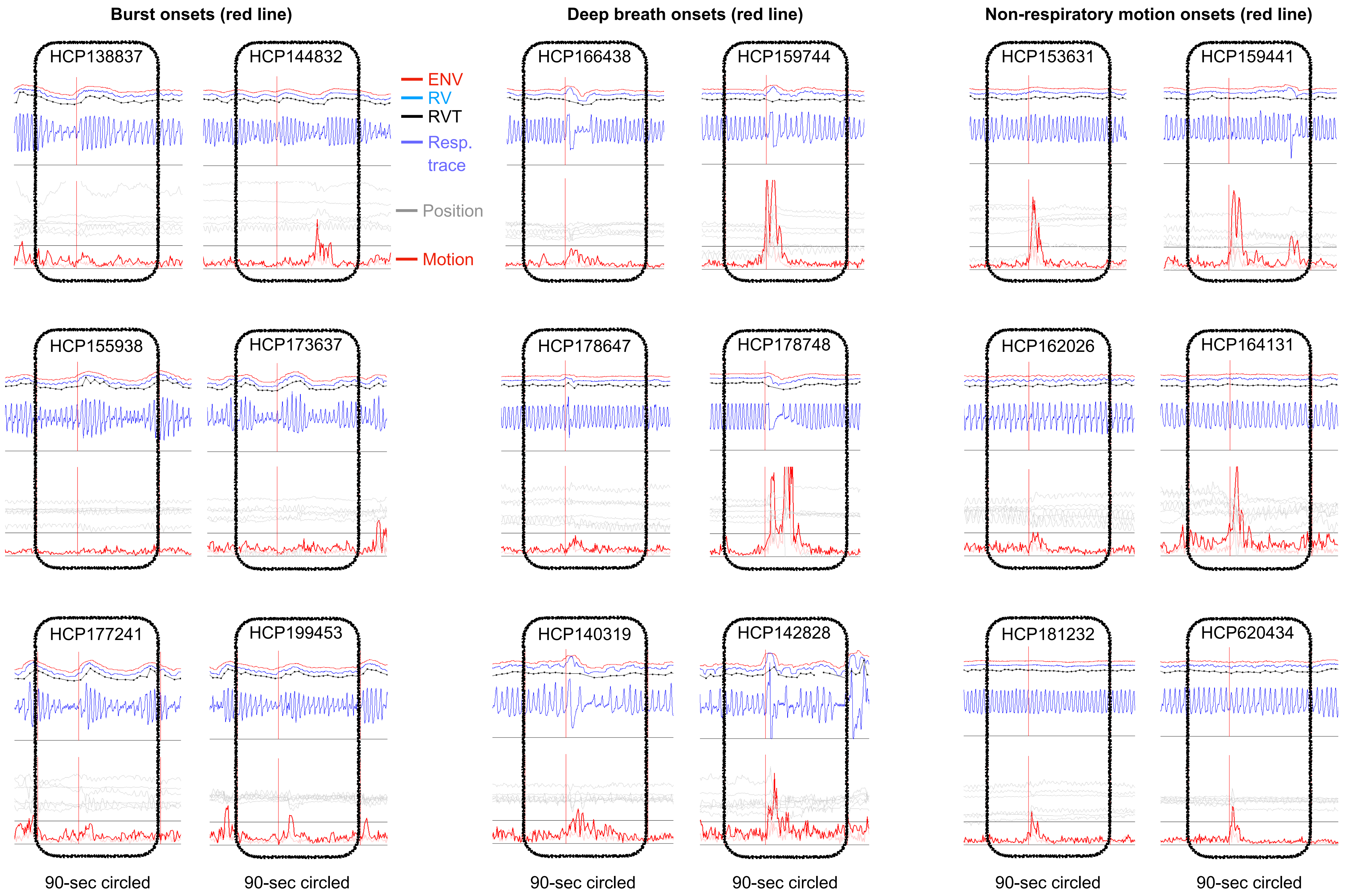

Supplementary Figure 7: Examples of onset placement. Red vertical lines denote onsets (t=0) and circles enclose the time spanning 30 seconds prior to onset to 60 seconds after onset. All 35 onsets for each of the 3 categories (burst, deep breaths, and non-respiratory motions) are illustrated in complete gray plots in Supplementary Movies 2, 3, and 4. Note the occasional motion time-locked to bursts, frequent motion time-locked to deep breaths, and abscence of respiratory abnormalities during non-respiratory motions. Scales are constant, and identical to those of other figures.

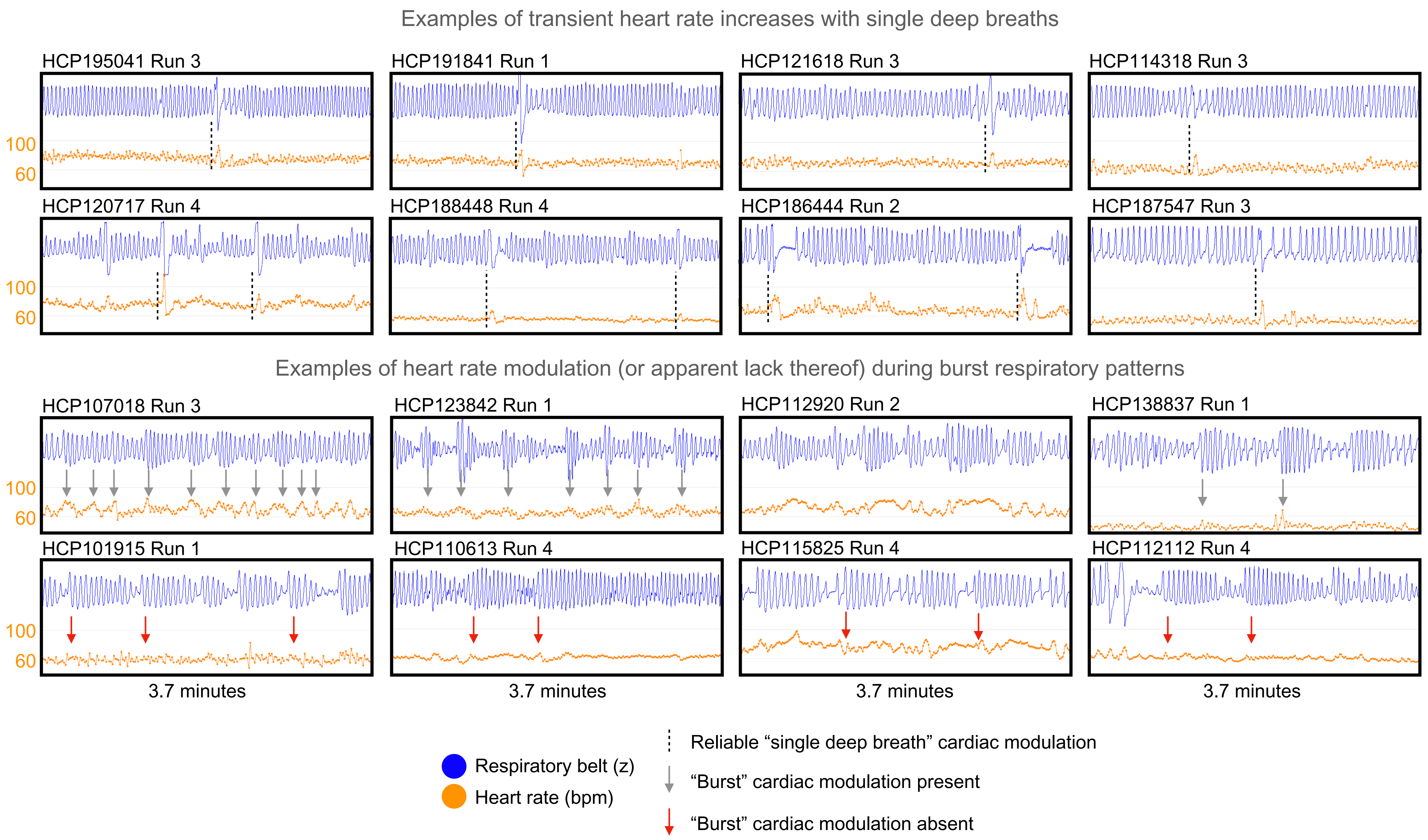

Supplementary Figure 8: Examples of heart rate modulation by respiratory pattern. Top rows show transient heart rate increases during and after single deep breaths. Bottom rows show modulation of heart rate by burst patterns in some subjects (gray arrows) but not in others (red arrows).

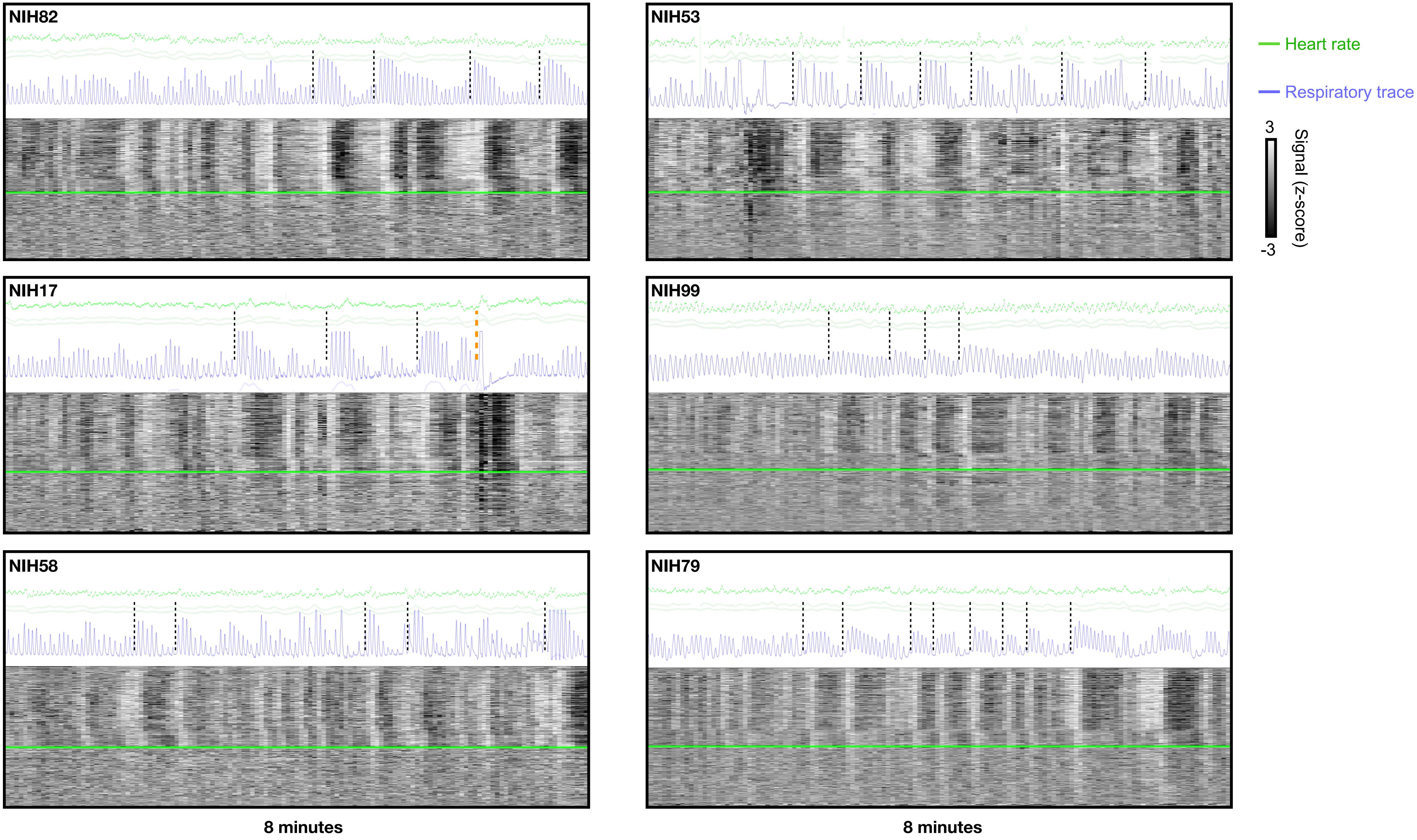

Supplementary Figure 9: Gray plots illustrating scans with notable bursts in NIH subjects of Power et al., 2017. Blue traces show respiratory belt data, green traces are heart rate calculated from pulse oximetry records, and grayscale images are of undenoised data in the style of Supplementary Figure 1. Dotted black lines mark the start of bursts, and a dotted orange line shows the start of a deep breath in NIH17. The first subject (NIH82) shows rather clear heart rate elevations at the start of bursts, but most bursts in other subjects have mild, unreliable, or undiscernable heart rate effects.

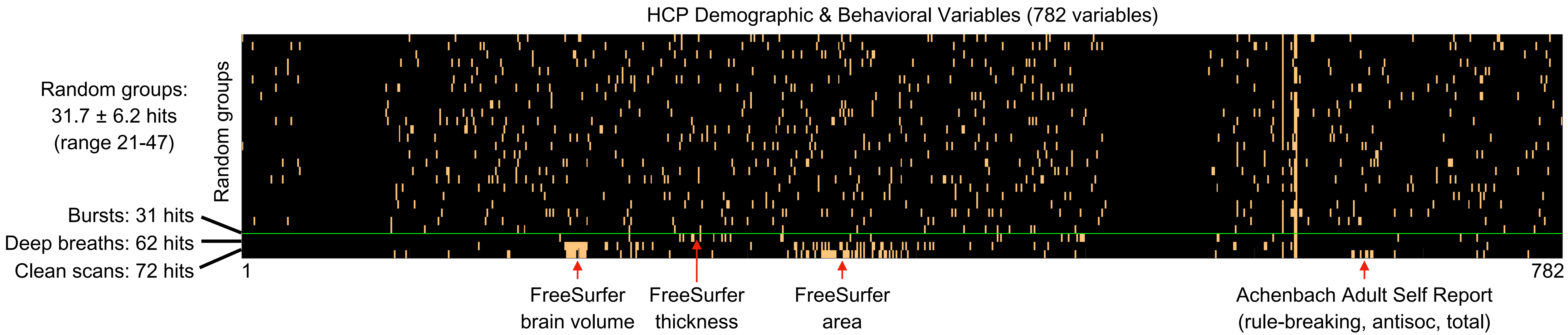

BURSTS		SINGLE DEEP BREATHS		CLEAN SCANS	
Notable Variables (31 hits)	Rank	Notable Variables (62 hits)	Rank	Notable Variables (72 hits)	Rank
FreeSurfer thickness (11 vars, all thin)	11-223	FreeSurfer volume (21 vars, all small)	14-248	FreeSurfer volume (15 vars, all small)	34-237
		FreeSurfer area (19 vars, all small)	0-234	FreeSurfer area (32 vars, all small)	2-230
SSAGA: alcohol quantity, dependence	9807, 9844	Tobacco use on same day	9795	SSAGA: alcohol quantity (2 vars, both low)	0, 8
Hemoglobin A1C	9924	Hematocrit (2 vars, both low)	24, 28	SSAGA: age at first tobacco use	9856
				Sleep latency (2 vars, both low)	143, 187
				Pain Interfering in Life	225
				Achenbach ASR (7 vars, all low)	19-219

FD	8545	FD	3807	FD	150
FD (filtered, 4 TR)	3735	FD (filtered, 4 TR)	4674	FD (filtered, 4 TR)	0
RVT std	9995	RVT std	3	RVT std	0
RV std	9768	RV std	98	RV std	0
ENV std	9727	ENV std	55	ENV std	0
RVT mean	7948	RVT mean	9517	RVT mean	7410
RV mean	1224	RV mean	9618	RV mean	10000
ENV mean	717	ENV mean	9870	ENV mean	10000

Sex (M/F)	14/7	Sex	5/16	Sex	6/15
Age (yrs)	28 ± 4	Age	29 ± 4	Age	29 ± 4
BMI	27 ± 6	BMI	26 ± 6	BMI	25 ± 5
Height (in)	68 ± 4	Height	66 ± 4	Height	66 ± 4
Weight (lb) *	180 ± 36	Weight	160 ± 33	Weight	159 ± 33

Supplementary Figure 10: Variables associated with bursts, with single deep breaths, and with scans without either phenomenon ("clean" scans). Groups of 21 subjects prominently displaying each kind of respiratory pattern were identified, and several hundred randomly composed groups of 21 subjects were also formed. These groups all underwent 10,000 permutation tests amidst all subjects to identify HCP variables associated with respiratory phenomena. The heatmap shows 25 of the random groups, and at bottom under the green line the 3 groups of interest. The groups of interest display differences clustered in several categories, summarized in the table (with ranks denoting the mean value amongst the 10,000 permutation values), and listed entirely in Table S2. In the heat map, the two blank areas of the chart are several dozen completeness measures for various parts of the HCP study, and the 60 individual questions related to the five factor model of personality. The 3 variables with very high rates of "significant" differences in the random groups (columns 617, 624, and 625) are questions about "OtherEndocrine_ProbOnset, Menstrual_MonthsSinceStop, and Menstrual_AgeStop.

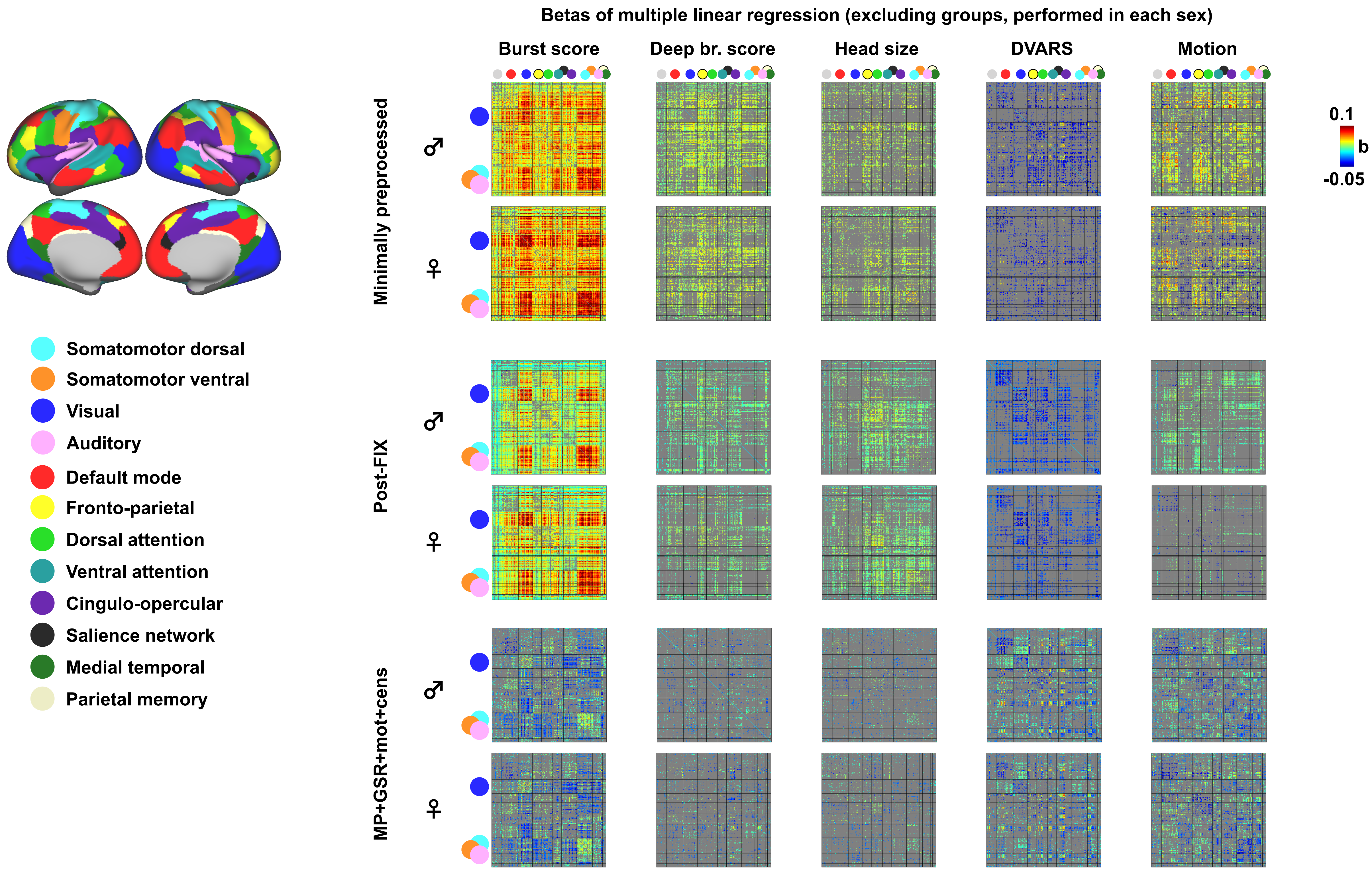

Supplementary Figure 11: Full network legend and full fits of the models shown in Figure 6C for 3 signal processing strategies. Scales and models are identical to those of Figure 6C.

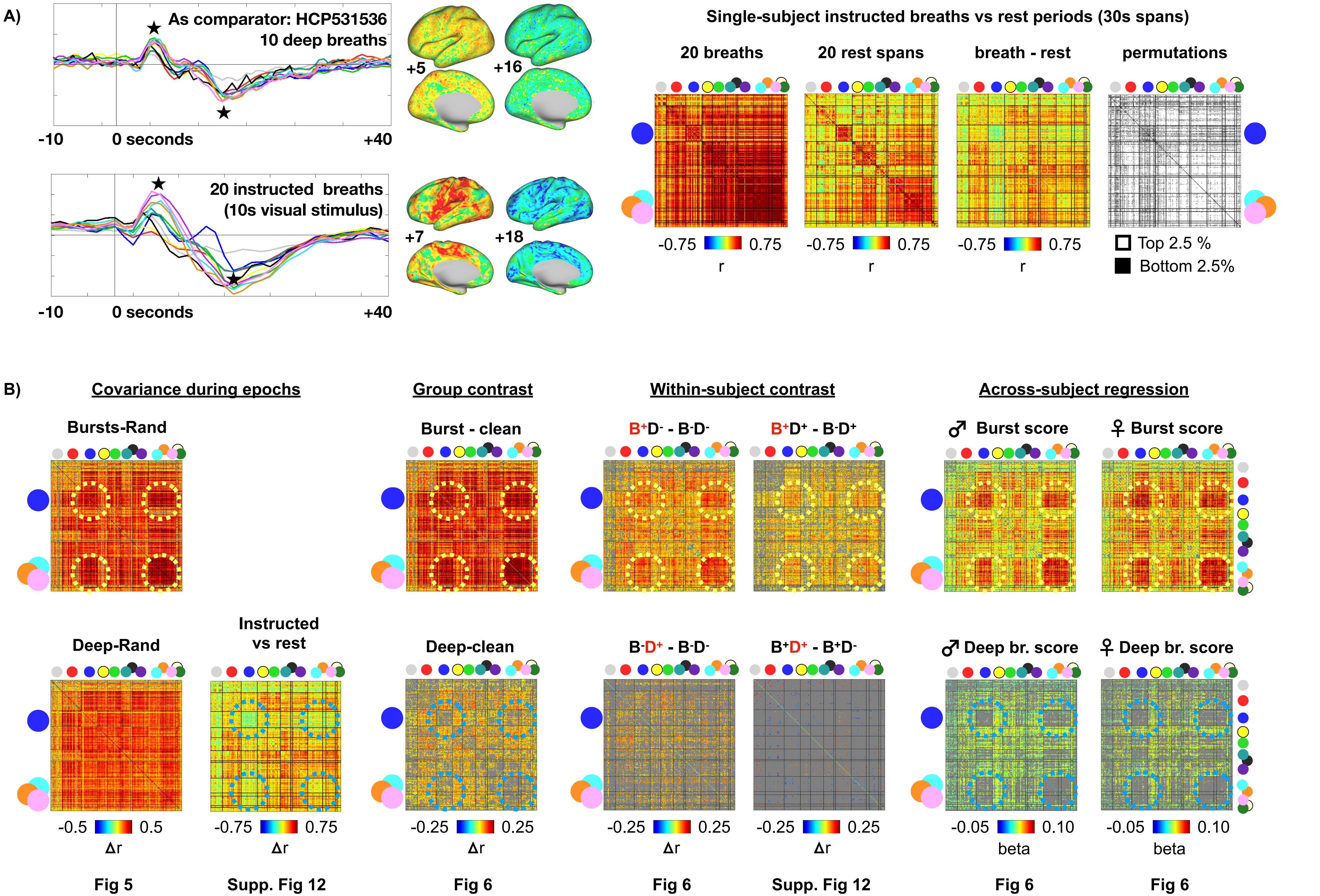

Supplementary Figure 12: Re-presentation of breathing patterns, with instructed breathing. A) An example of a single HCP subject with many deep breaths, mean signal in each network over 10 deep breaths are shown. A single subject performing widely-spaced instructed deep breaths is shown below, with similar timeseries (data reported in Power et al., 2020). Surfaces show locations of peak and trough signal changes, with much stronger signals in the multi-echo denoised data (with twice as many breath examples). The matrices at right show correlations in the instructed breathing scans from timepoints 0-30 (breaths), 35-65 (rest), and the difference and permutation tests of those matrices. B) Re-arrangement of previously presented matrices by breathing type. Also shown in the within-subject section are the within-subject contrasts in the presence of both breathing patterns (i.e., B^+D^+ - B^-D^+, contrasting bursts with deep breath always present, rather than when the other pattern is always absent).

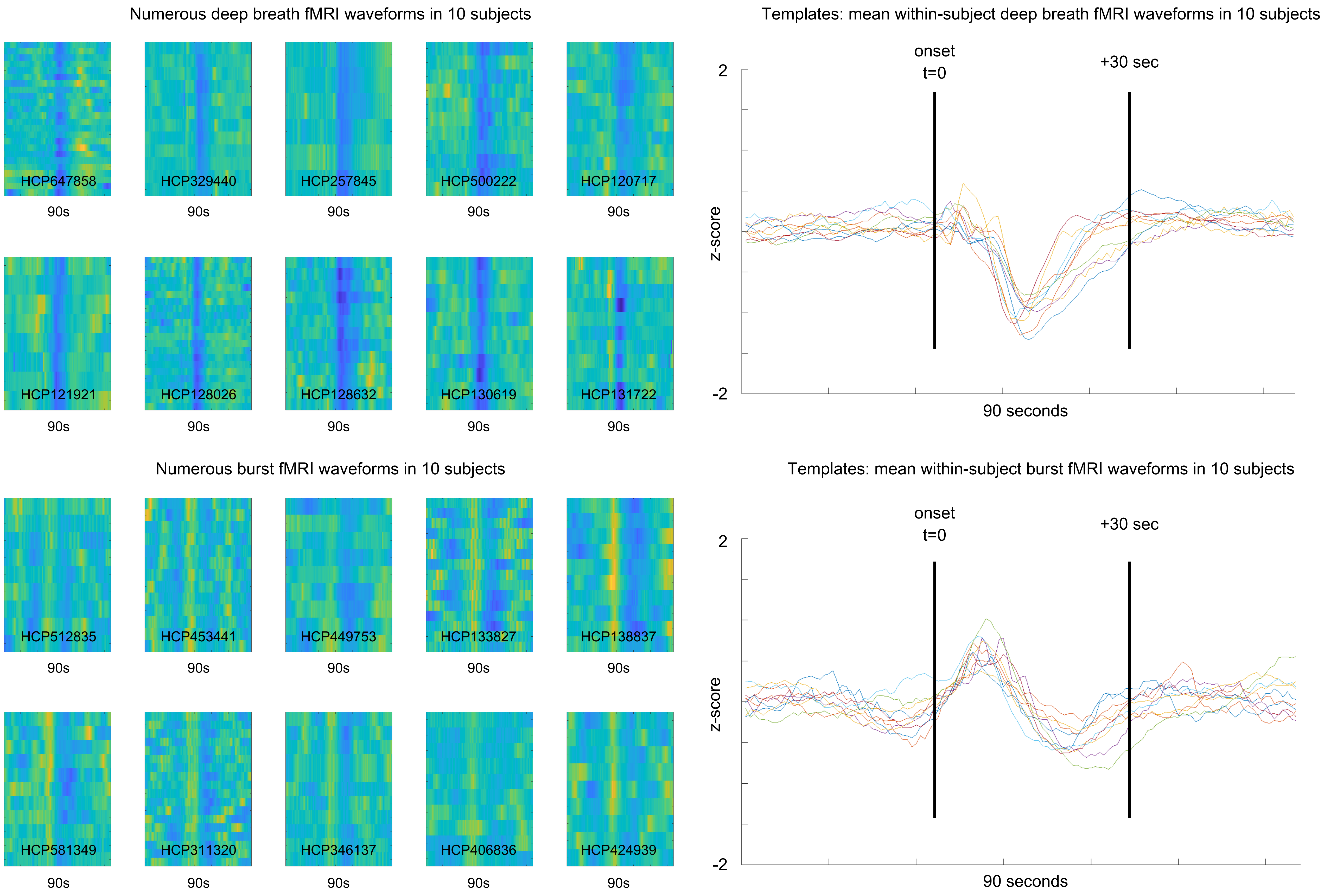

Supplementary Figure 13: Template construction for respiratory events. 10 subjects with numerous deep breaths and 10 subjects with numerous bursts were identified, onsets were visually marked and permitted to vary slightly algorithmically (see Supplementary Note 4) to maximize cross-correlation of global signal surrounding the events. All global signals are shown for all subjects for all events as heatmaps at left. Mean waveforms are shown at right, defining the fMRI templates of respiratory events.

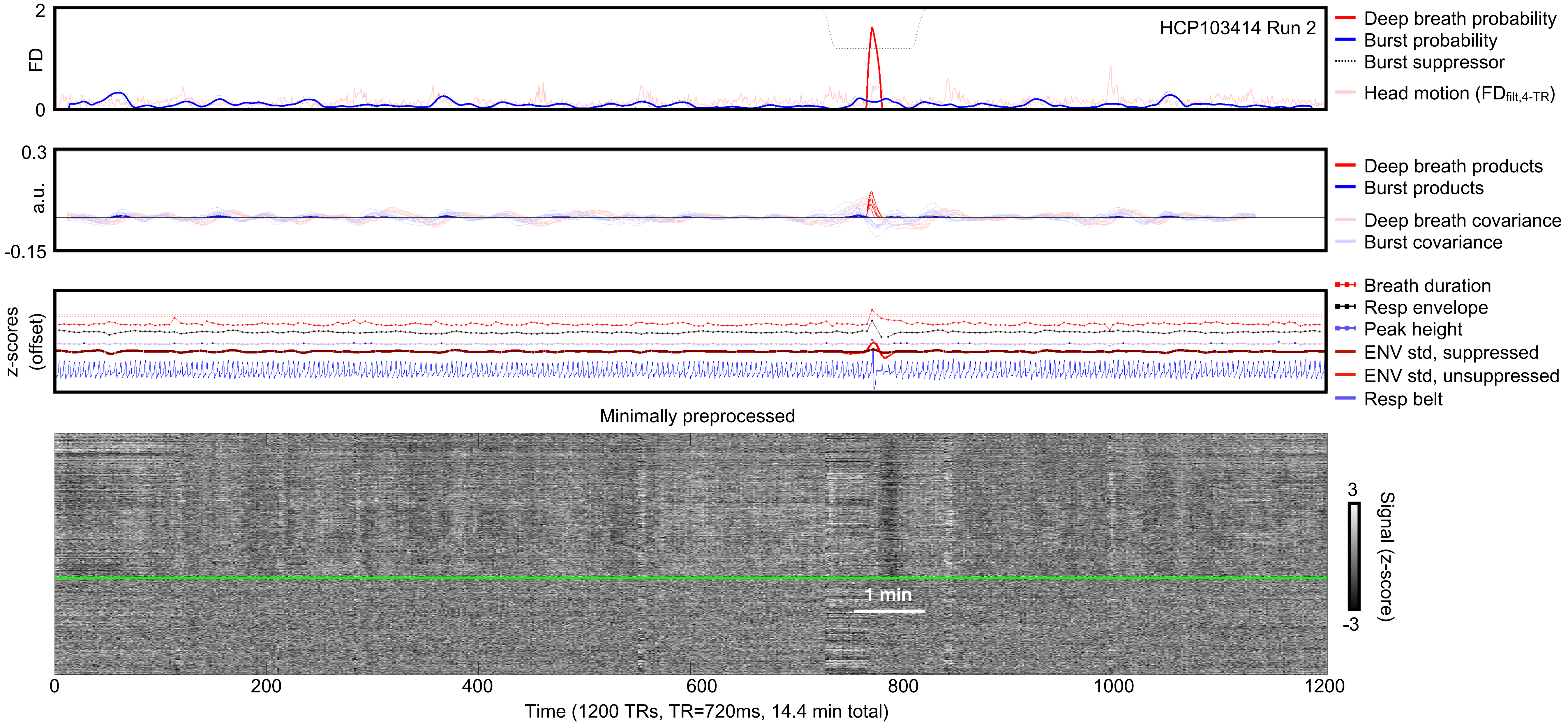

Supplementary Figure 14: Identification of deep breaths. Peak-finding breath duration is the red dotted line of third panel, respiratory envelope is the black dotted line (which can augment breath duration if both high), and peak-finding peak amplitude is in blue dotted line (gets multiplied into breath duration, if high, only at the darker points qualifying for multiplication). Together, these factors create the red spike in the top panel, the probability vector for deep breaths. In the second panel, the faded red bundle of traces are the 10 covariance vectors for deep breath templates to the global fMRI timeseries of the scan. These covariance vectors are multiplied by the red probability trace to provide the bright red traces of the second panel, reflecting the joint action of the probability vector and the fMRI covariance vectors to indicate the occurrence and severity of a deep breath. The area under the curves is the index of deep breaths for the scan.

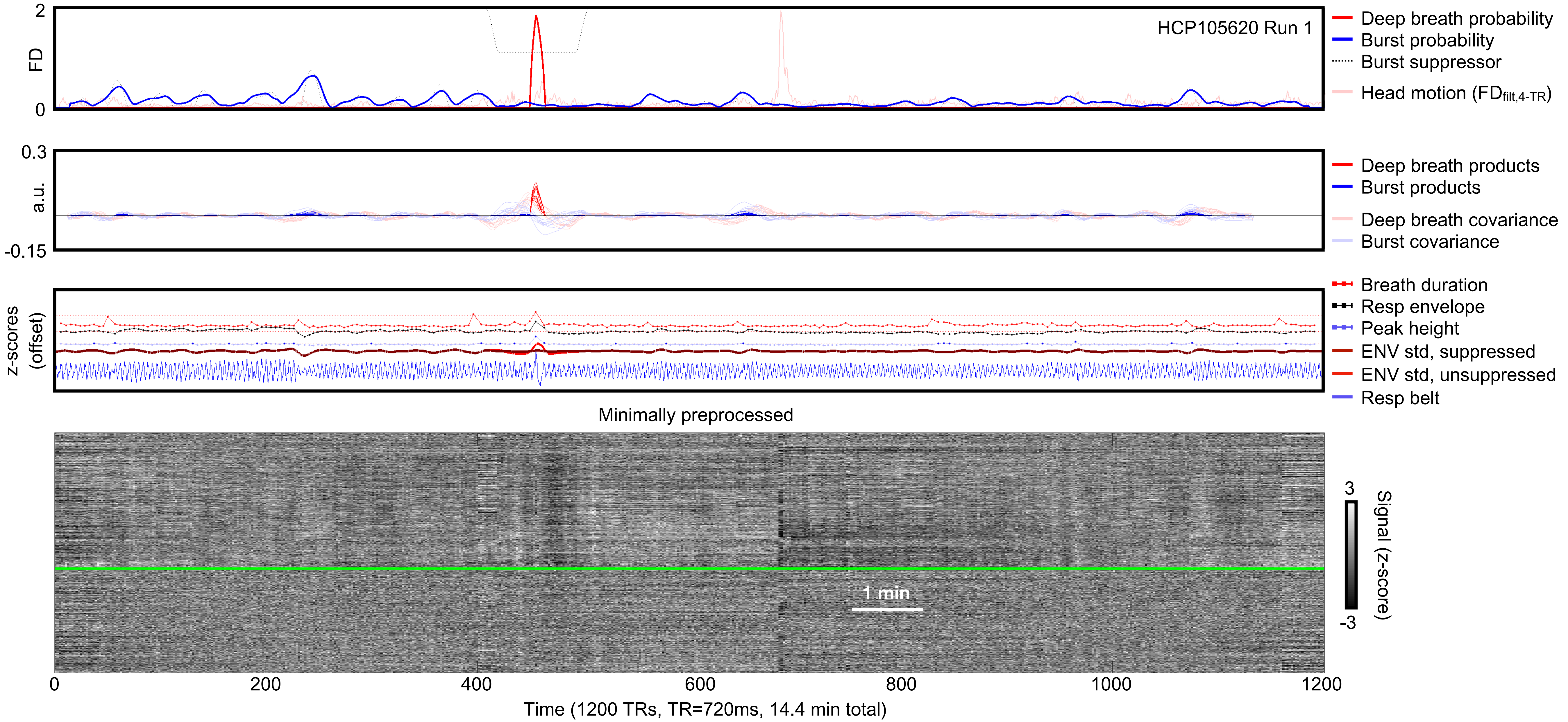

Supplementary Figure 15: Identification of deep breaths. As for Supplementary Figure 14, in a different subject with a single deep breath around timepoint 450. Note the large motion spike midway through the scan near timepoint 700, producing temporally limited effects that are very distinct from those of deep breaths. This scan also illustrates the algorithm not identifying long breaths per se as deep breaths, see the numerous spikes in breath duration (dotted red line of third panel) not flagged as probable deep breaths.

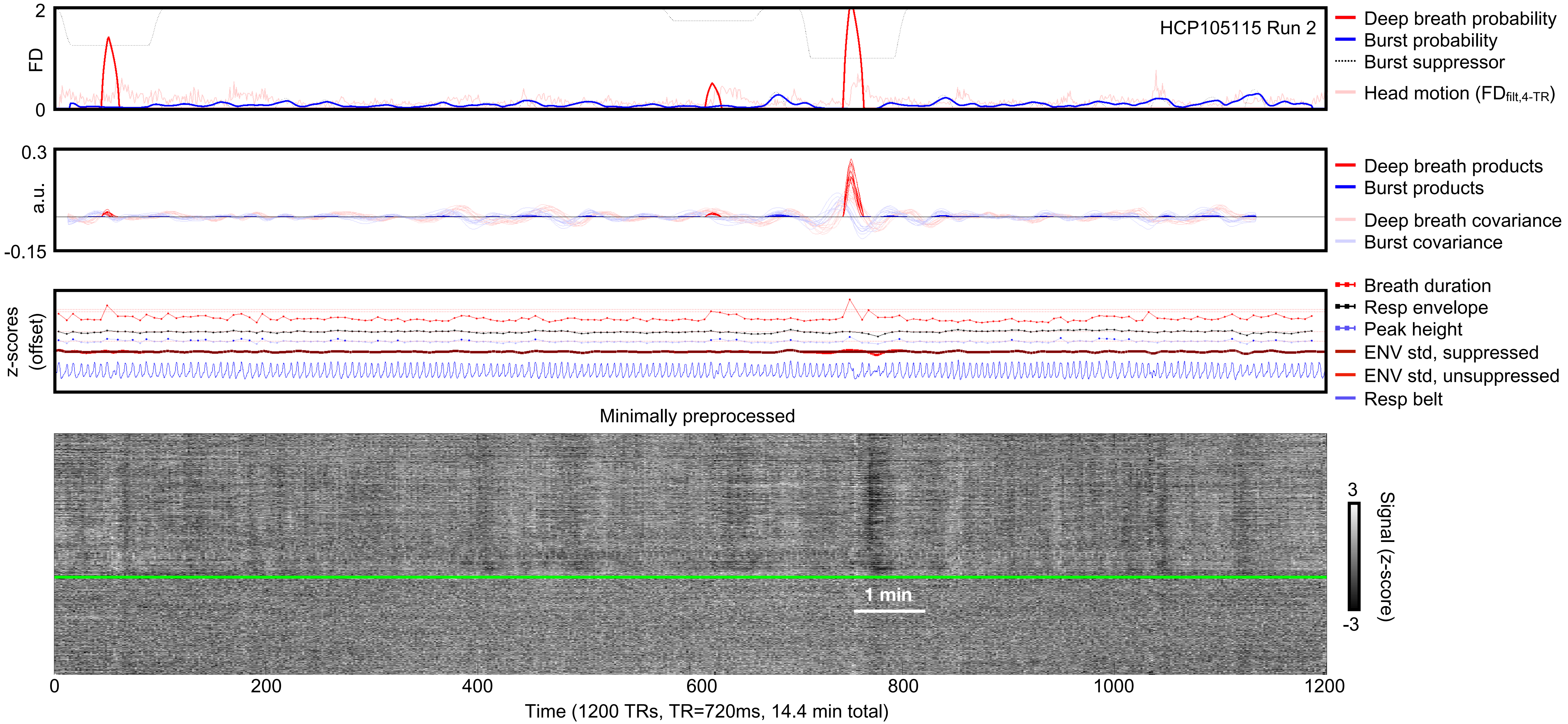

Supplementary Figure 16: Identification of deep breaths, illustrating interaction of event probability and fMRI template covariance. Panels are as in Supplementary Figure 14, but now note 3 potential deep breaths identified in the red probability vectors of the top panel, whereas only a single large product spike is seen in the second panel, due to deep breath fMRI template only matching the fMRI timeseries strongly in the deep breath near timepoint 800.

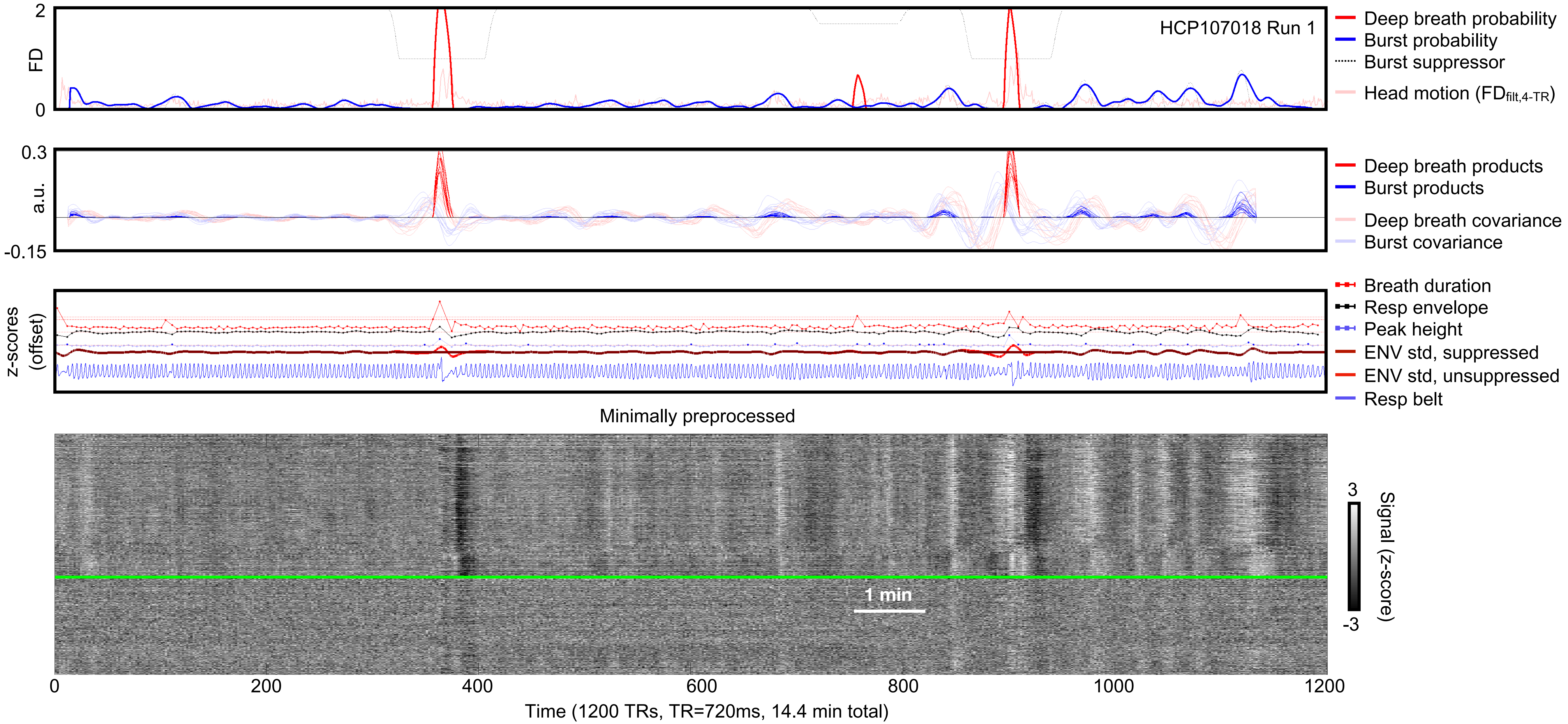

Supplementary Figure 17: Identification of multiple deep breaths, and bursts. Panels are as in Supplementary Figure 14. Two deep breaths are correctly identified, a third is seen as possible near timepoint 800 but has no corresponding evidence in the deep breath fMRI template covariance traces and is thus negated with no effect in the product trace. Additionally, bursts emerge in the second half of the scan, and are properly identified by the blue product traces.

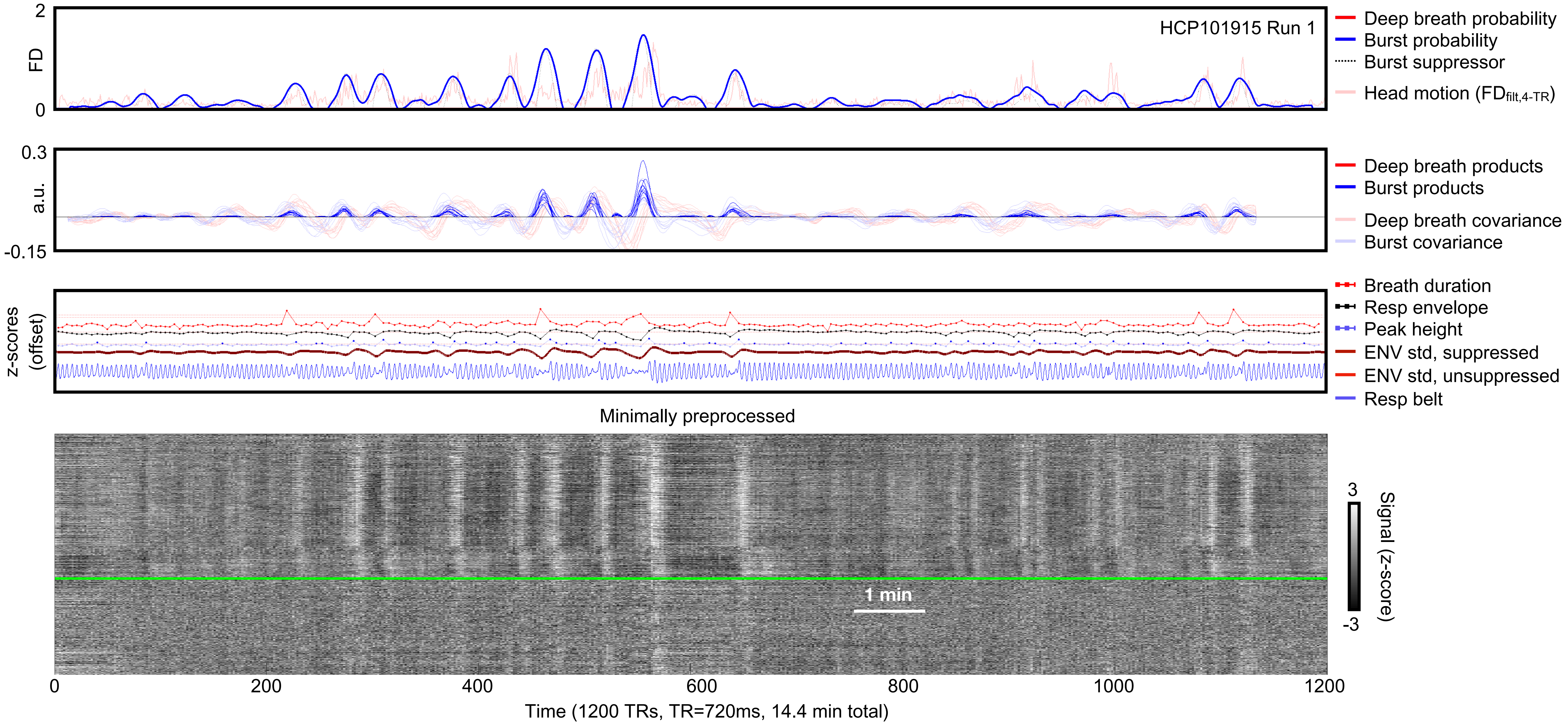

Supplementary Figure 18: Identification of bursts. Panels are as in Supplementary Figure 14. Several bursts are identified, none are mis-identified as deep breaths. Note that tapers of bursts display slowed breathing (dotted red line, sometimes artificially slowed by peak-finding not detecting the very small breaths) but that these slow breaths occur with low envelope and low peak height usually, and are thus unlikely to be identified as deep breaths in the algorithm, which augments only in instances with high envelope and high peak height.

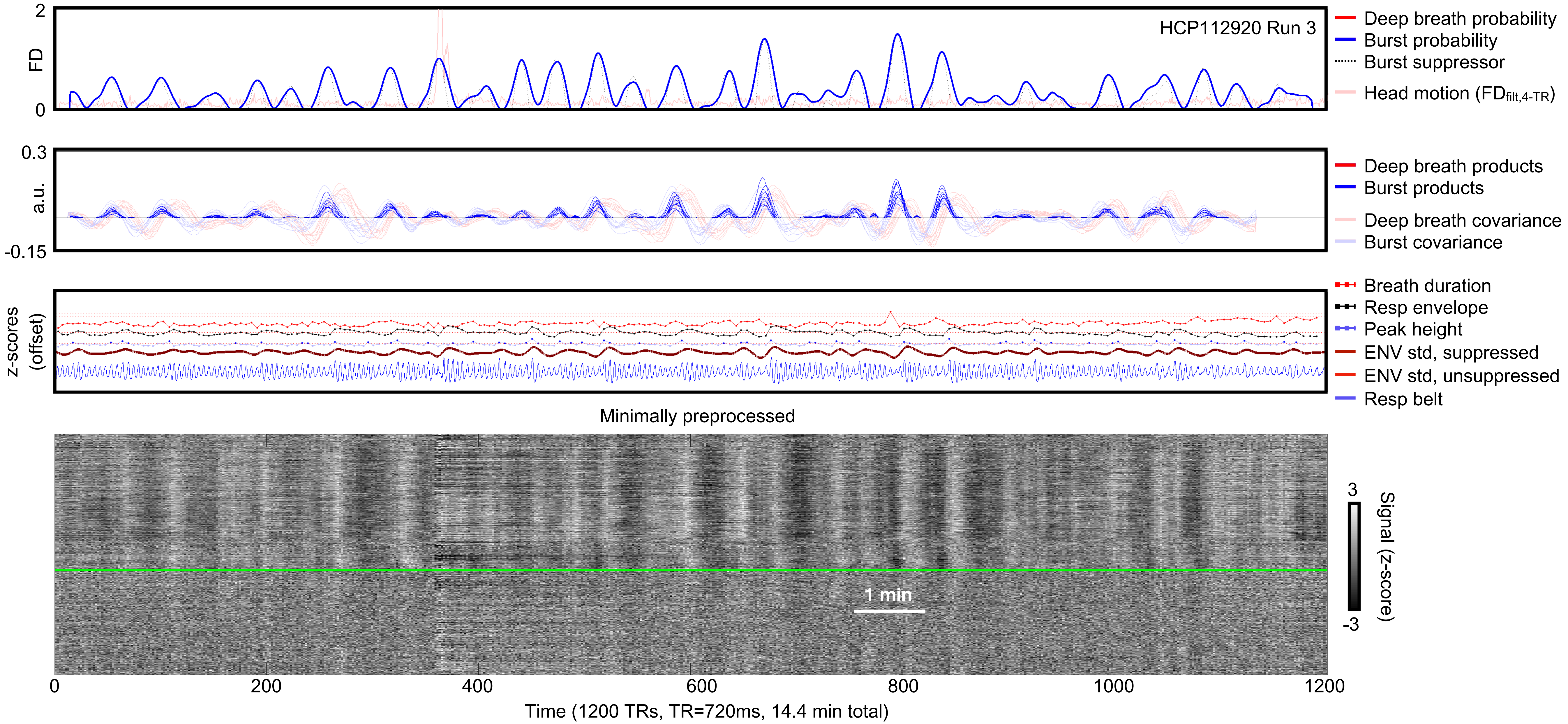

Supplementary Figure 19: Identification of bursts. Panels are as in Supplementary Figure 14. Another instance of a scan with pure burst pattterning.

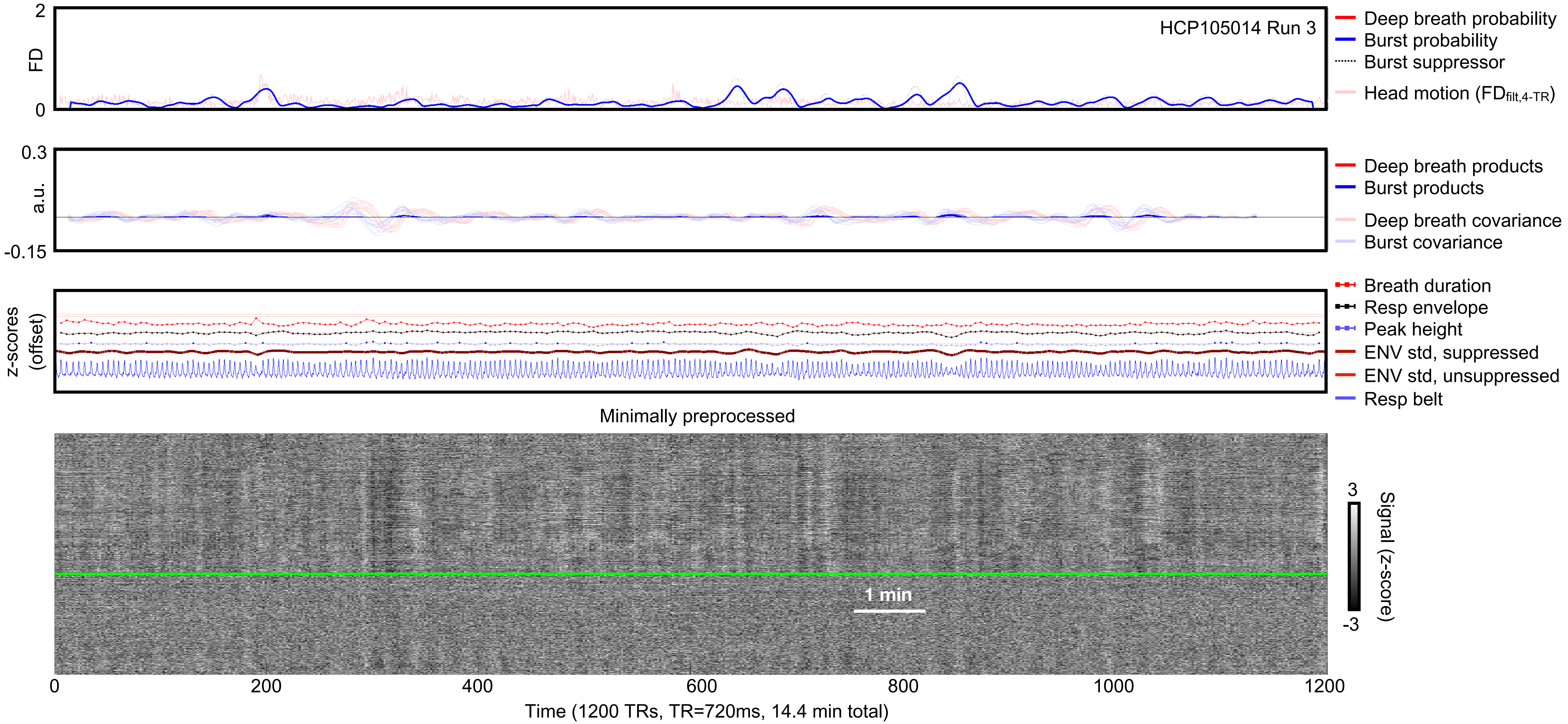

Supplementary Figure 20: A scan with no events. Panels are as in Supplementary Figure 14. No substantial respiratory events are identified at all.

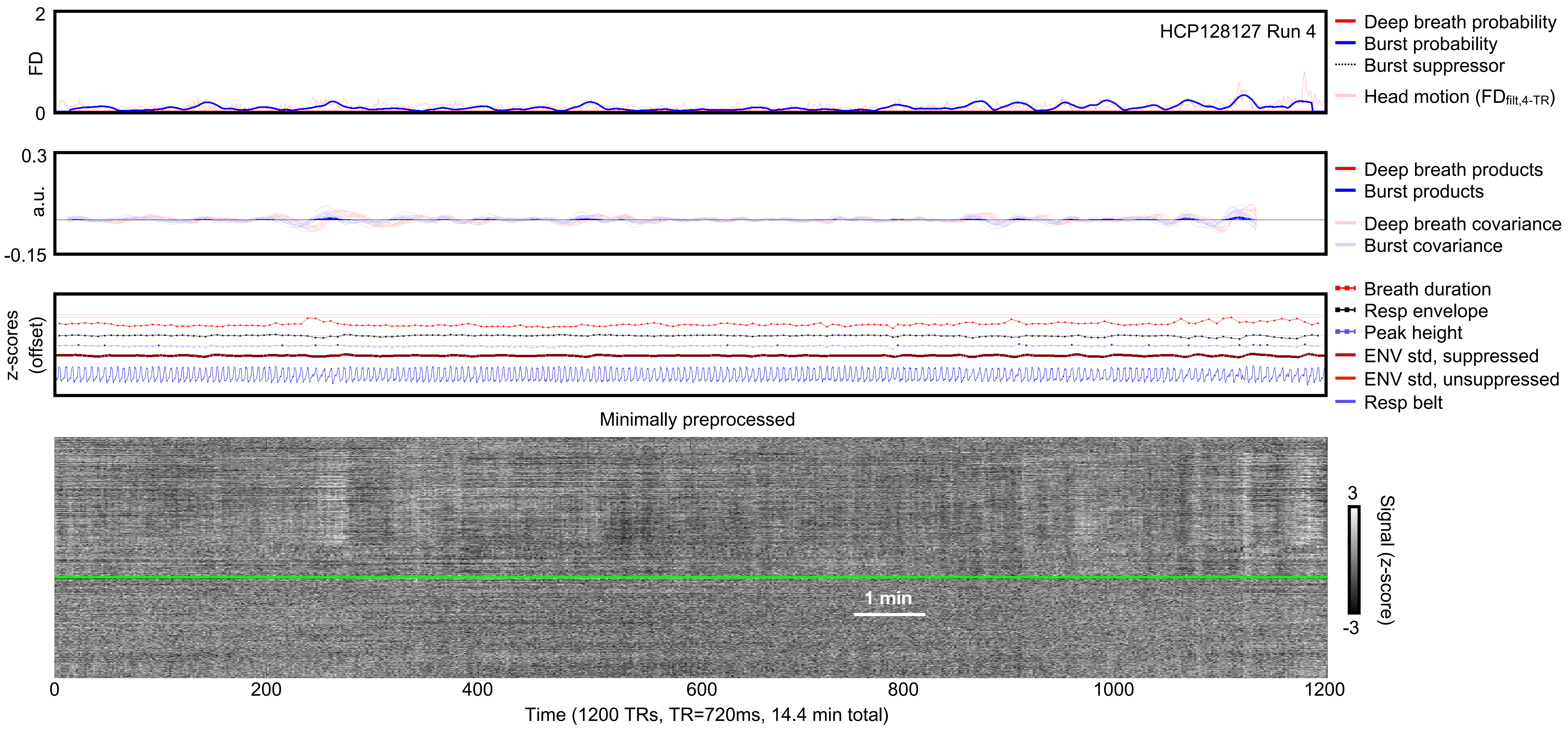

Supplementary Figure 21: A scan with no events. Panels are as in Supplementary Figure 14. No substantial respiratory events are identified at all.

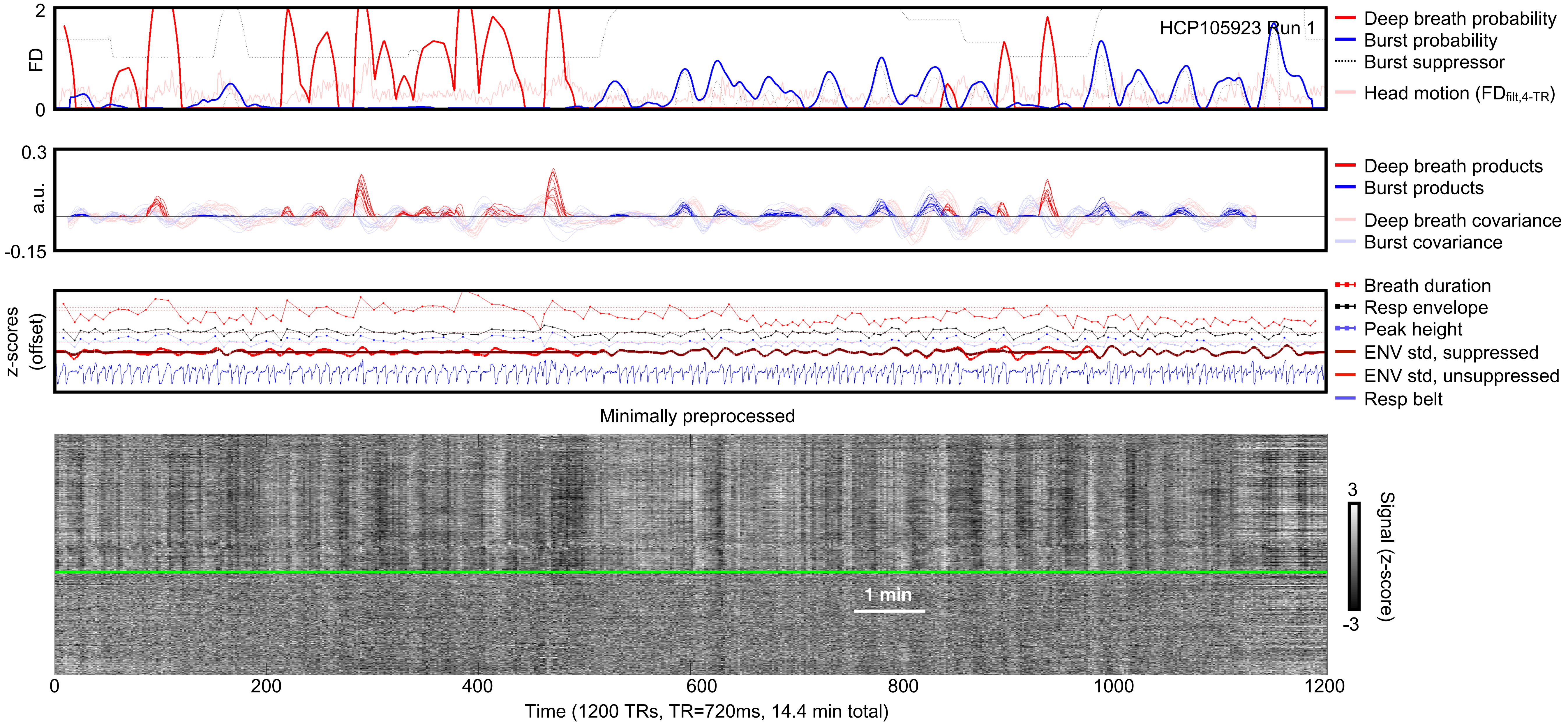

Supplementary Figure 22: A scan with haphazard breathing. Panels are as in Supplementary Figure 14. Visual examination of respiration gives little clarity on how such a scan should be scored, for the timeseries are incredibly noisy, and the breathing style is irregular. Note the high variability in breath duration (the dotted red trace of the third panel). Variance in this breath duration measure is used to downweigh area under curve scores in order to dampen the influence of difficult-to-assess scans like this one.

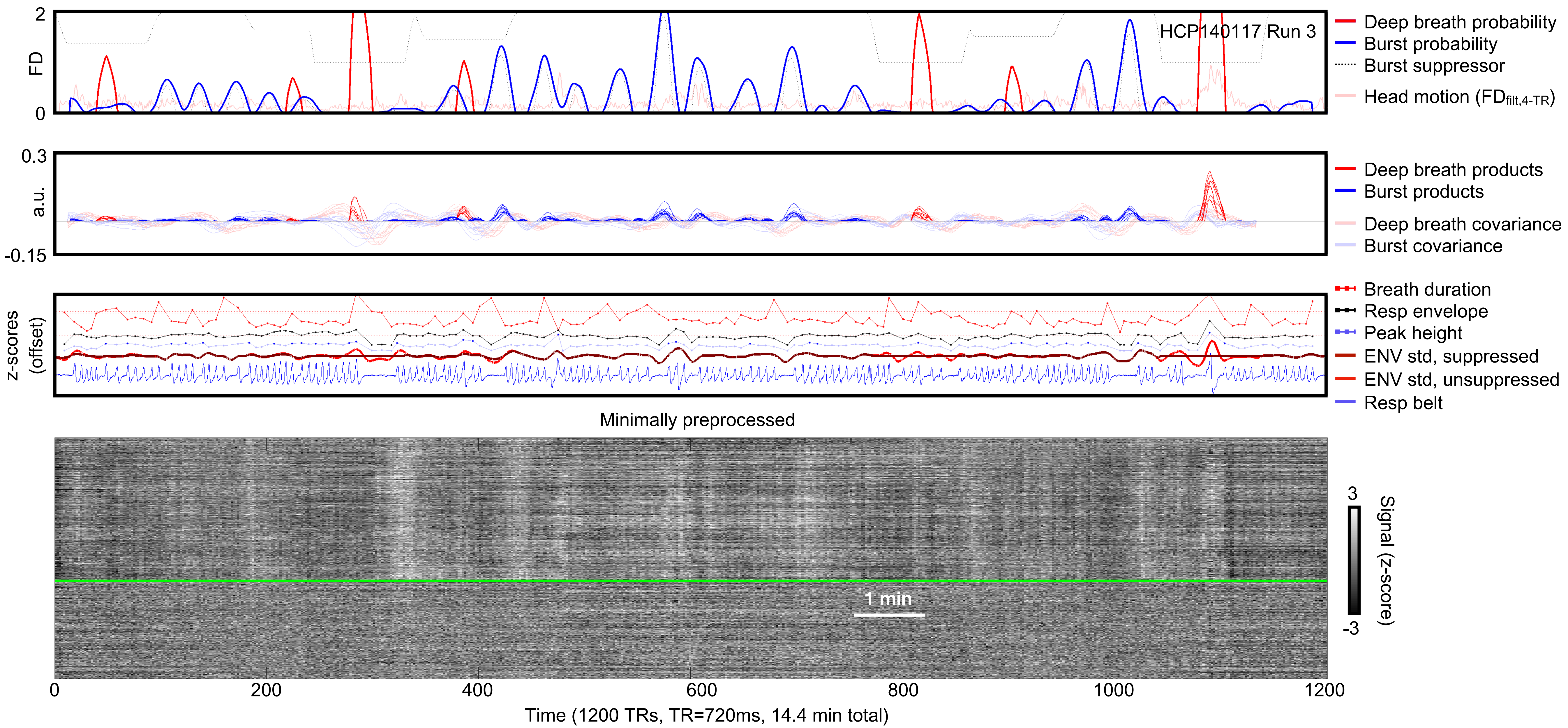

Supplementary Figure 23: A scan with haphazard breathing. Panels are as in Supplementary Figure 14. Visual examination of respiration gives little clarity on how such a scan should be scored, for the timeseries are noisy, and the breathing style is irregular. There is one fairly clear deep breath at timepoint 1100. Note the high variability in breath duration (the dotted red trace of the third panel). Variance in this breath duration measure is used to downweigh area under curve scores in group-level analyses, in order to dampen the influence of difficult-to-assess scans like this one.
