# Supplementary material for: Prevalent and sex-biased breathing patterns modify functional connectivity MRI in young adults
Source: Nat Commun. 2020 Oct 20;11:5290. doi: 10.1038/s41467-020-18974-9 (PMC7576607; doi:10.1038/s41467-020-18974-9)
Supplement: Supplementary file 2 — Description of Additional Supplementary Files [file 41467_2020_18974_MOESM2_ESM.pdf]

Description of Additional Supplementary Files

Supplementary Data 1: Onsets of deep breaths, bursts, and motions in sets of 35 subjects.

Supplementary Data 2: Results of group contrasts for all variables in the HCP dataset, listed for each of the 3 groups. For comparison, results from 50 randomly composed and equally sized groups are listed.

Supplementary Movie 1: Gray plots of all 440 HCP subjects. The top panel shows head position traces (gray) and motion traces (red), the second panel shows DVARS traces before (light green) and after (dark green) FIX-ICA, the third panel shows in blue the respiratory belt trace and the derived ENV (red), RV (blue), and RVT (black) traces. The fourth panel shows as a grayscale heat map all in-brain timeseries of the fMRI scan before and after FIX-ICA (as successive frames of the movie). Download at https://osf.io/u35f8/

Supplementary Movie 2: Gray plots of 35 deep breaths, with red bars marking t = -30, 0, and +60 seconds relative to event onset.

Supplementary Movie 3: Gray plots of 35 bursts, with red bars marking t = -30, 0, and +60 seconds relative to event onset.

Supplementary Movie 4: Gray plots of 35 non-respiratory head motions, with red bars marking t = -30, 0, and +60 seconds relative to event onset.

Supplementary Movie 5: Movies of fMRI signals on brain surfaces, showing the average spatiotemporal patterns from the 35 instances of deep breaths, bursts, and non-respiratory head motions. A random comparator is derived from the motion group, by selecting random onset times.
