# Supplementary material for: Prevalent and sex-biased breathing patterns modify functional connectivity MRI in young adults
Source: Nat Commun. 2020 Oct 20;11:5290. doi: 10.1038/s41467-020-18974-9 (PMC7576607; doi:10.1038/s41467-020-18974-9)
Supplement: Supplementary file 9 — Reporting Summary [file 41467_2020_18974_MOESM9_ESM.pdf]

nature research

Corresponding author(s): Jonathan Power

Last updated by author(s): Sep 1, 2020

Reporting Summary

Nature Research wishes to improve the reproducibility of the work that we publish. This form provides structure for consistency and transparency in reporting. For further information on Nature Research policies, see Authors & Referees and the Editorial Policy Checklist .

Statistics

For all statistical analyses, confirm that the following items are present in the figure legend, table legend, main text, or Methods section.

n/a	Confirmed	
☐	☒	The exact sample size (*n*) for each experimental group/condition, given as a discrete number and unit of measurement
☐	☒	A statement on whether measurements were taken from distinct samples or whether the same sample was measured repeatedly
☐	☒	The statistical test(s) used AND whether they are one- or two-sided<br>*Only common tests should be described solely by name; describe more complex techniques in the Methods section.*
☐	☒	A description of all covariates tested
☐	☒	A description of any assumptions or corrections, such as tests of normality and adjustment for multiple comparisons
☐	☒	A full description of the statistical parameters including central tendency (e.g. means) or other basic estimates (e.g. regression coefficient) AND variation (e.g. standard deviation) or associated estimates of uncertainty (e.g. confidence intervals)
☐	☒	For null hypothesis testing, the test statistic (e.g. *F*, *t*, *r*) with confidence intervals, effect sizes, degrees of freedom and *P* value noted<br>*Give P values as exact values whenever suitable.*
☒	☐	For Bayesian analysis, information on the choice of priors and Markov chain Monte Carlo settings
☒	☐	For hierarchical and complex designs, identification of the appropriate level for tests and full reporting of outcomes
☐	☒	Estimates of effect sizes (e.g. Cohen's *d*, Pearson's *r*), indicating how they were calculated

Our web collection on statistics for biologists contains articles on many of the points above.

Software and code

Policy information about availability of computer code

Data collection	No software were used for data collection
Data analysis	Code is custom-written in Matlab 2019b (v1.8.0_202), code is available at https://osf.io/u35f8/ except redactions to protect restricted HCP information

For manuscripts utilizing custom algorithms or software that are central to the research but not yet described in published literature, software must be made available to editors/reviewers. We strongly encourage code deposition in a community repository (e.g. GitHub). See the Nature Research guidelines for submitting code & software for further information.

Data

Policy information about availability of data

All manuscripts must include a data availability statement. This statement should provide the following information, where applicable:

- Accession codes, unique identifiers, or web links for publicly available datasets
- A list of figures that have associated raw data
- A description of any restrictions on data availability

HCP data are publicly available at www.humanconnectome.org. Source data are provided with this paper as Source Data files for Figs 3 and 4, available at https://osf.io/u35f8/ (DOI 10.17605/OSF.IO/U35F8), as are all supplemental movies (~2GB). Certain HCP data are restricted to protect subject privacy, such as genetic, medical, and neuropsychiatric information. The three 21-subject groups of this paper demonstrate associations to such variables, and their identities are only available by Subject Key within HCP Restricted Access accounts and are otherwise obscured in the publicly available data.

Field-specific reporting

Please select the one below that is the best fit for your research. If you are not sure, read the appropriate sections before making your selection.

- [x] Life sciences
- [ ] Behavioural & social sciences
- [ ] Ecological, evolutionary & environmental sciences

For a reference copy of the document with all sections, see nature.com/documents/nr-reporting-summary-flat.pdf

Life sciences study design

All studies must disclose on these points even when the disclosure is negative.

Sample size	N=440 healthy young adults (often part of sibling sets); this is the maximal subset of the 900-subject release with high quality physiology records
Data exclusions	Original sample was 900 subjects, 460 were excluded due to poor/corrupted physiology data quality, leaving N=440 studied; quality exclusion criteria were preestablished and published in Power et al., 2019.
Replication	Multiple independent analytic techniques converge on the same findings (group contrasts, regression analyses in independent subjects, within-subject changes across scans). Congruent main conclusions are drawn across all analytic techniques.
Randomization	Permutation testing and bootstrap analyses are used, drawing from unrelated subjects
Blinding	Investigators were blind to all properties of subjects other than their respiratory traces and heatmaps of their fMRI timeseries - we knew nothing of patient sex, or of spatial patterns in the fMRI signals, or anything else about a given subject at the time of relevant assessments.

Reporting for specific materials, systems and methods

We require information from authors about some types of materials, experimental systems and methods used in many studies. Here, indicate whether each material, system or method listed is relevant to your study. If you are not sure if a list item applies to your research, read the appropriate section before selecting a response.

Materials & experimental systems

n/a	Involved in the study	
x		Antibodies
x		Eukaryotic cell lines
x		Palaeontology
x		Animals and other organisms
	x	Human research participants
x		Clinical data

Methods

n/a	Involved in the study	
x		ChIP-seq
x		Flow cytometry
	x	MRI-based neuroimaging

Human research participants

Policy information about studies involving human research participants

Population characteristics	Subjects were N=440 healthy young adults recruited as part of the Human Connectome Project, a completed and now publicly available dataset funded by the NIH. Subjects were 20-40 years old, living in the American Midwest, and denied neuropsychiatric illness or hypertension or diabetes. Studied subjects comprised 228 males, 212 females, with average age 28.6. This is one of the largest datasets of respiratory records in awake, healthy young adults in existence.
Recruitment	Subjects were recruited via a Missouri state registry of siblings sets that included twins. Self-selection bias may have occurred if eligible subjects declined to participate, but this is unlikely to affect the present results, which are prevalent across subjects.
Ethics oversight	The HCP-YA protocol was approved by The Washington University in St. Louis Institutional Review Board, WUSTL DHHS Federalwide Assurance #FWA00002284 BJH DHHS Federalwide Assurance #FWA00002281 SLCH DHHS Federalwide Assurance #FWA00002282, and the imaging and physiology data under study, and sex of participants, are publicly available. Full demographic variables (e.g., height) were accessed via the Restricted Use agreement of the HCP and are reported only at a group level without individual identification, as specified in the agreement. No local IRB was involved in this study of public data.

Note that full information on the approval of the study protocol must also be provided in the manuscript.

Magnetic resonance imaging

Experimental design

Design type	Resting state
Design specifications	4 14.4 min resting state scans, 2 scans on 2 days each
Behavioral performance measures	respiratory belts and pulse oximetry waveforms

Acquisition

Imaging type(s)	functional
Field strength	3T
Sequence & imaging parameters	HCP protocol: TR = 720 ms, TE = 33.1 ms, flip angle 52 degrees, multi-band factor 8, and 2 mm isotropic voxels.
Area of acquisition	whole brain

Diffusion MRI ☐ Used ☒ Not used

Preprocessing

Preprocessing software	The "stock" HCP preprocessing pipeline, data were obtained after preprocessing was complete, the full pipeline is presented in Glasser et al., 2013 in Neuroimage: DOI: 10.1016/j.neuroimage.2013.04.127
Normalization	Data were in fs_LR surfaces and also MNI space after registration, linear affine transforms were used for motion and nonlinear FNIRT was used to register to atlas space, among other normalizations
Normalization template	MNI
Noise and artifact removal	Most of the manuscript focuses on the fMRI data before denoising (i.e., "minimally preprocessed"), in order to characterize signals and effects arising from respiratory events during scanning. Data after FIX-ICA denoising are shown for all scans individually in an online movie, illustrating that these respiratory signals are not removed in general, and denoised data are specifically studied in terms of functional connectivity effects at the end of the paper after common steps of global signal regression, motion parameter regression, and censoring (zscore(DVARS) > 2)
Volume censoring	Censoring was not done in most analyses of "raw" data, but its effect is illustrated in Fig 6

Statistical modeling & inference

Model type and settings	The only timeseries modeling was of mean whole-brain fMRI signals, defining ~60-second "event responses" to respiratory events whose onsets were temporally defined by the respiratory belt records.
Effect(s) tested	Effects of bursts and deep breaths are modeled via FIR, with onsets defined by respiratory belt records.

Specify type of analysis: ☐ Whole brain ☐ ROI-based ☒ Both

Anatomical location(s)	The surface parcellation of Gordon et al., 2016 (DOI: 10.1093/cercor/bhu239) was used at times, which groups all cortical vertex signals into regions of interest with pre-defined cluster structure
Statistic type for inference (See Eklund et al. 2016)	Voxel-wise or cluster-wise inference was not performed.
Correction	Permutation tests (10,000 cycles) are used to establish significance.

Models & analysis

n/a	Involved in the study
☐	☒ Functional and/or effective connectivity
☒	☐ Graph analysis
☒	☐ Multivariate modeling or predictive analysis

Functional and/or effective connectivity	In functional connectivity terms, dependent variables were "global functional connectivity", defined as the median voxelwise gray matter Pearson correlation in a scan (averaged over 4 scans, at times), or simply the Pearson correlation matrix between the Gordon parcels above in the scans (averaged over 4 scans, at times). These dependent variables were modeled in different analyses (via ANCOVA, multiple linear regression, etc.) by independent variables such as variance in the respiratory trace, intracranial volume, sex, or rater or algorithmic scorings of a subject's 4 scans.
