# Supplementary material for: Prevalent and sex-biased breathing patterns modify functional connectivity MRI in young adults
Source: Nat Commun. 2020 Oct 20;11:5290. doi: 10.1038/s41467-020-18974-9 (PMC7576607; doi:10.1038/s41467-020-18974-9)
Supplement: Supplementary file 10 — Source Data [file 41467_2020_18974_MOESM10_ESM.zip › Figure4/README.rtf]

Simply direct Matlab to this folder and run “fig4simple()”This is a stripped down version of the figure. Group statistics are represented in pre-defined variables to obscure the identify of the groups. For similar reasons, the figures in this script are made with all subjects, whereas in the manuscript the groups are excluded in certain analyses as indicated in the text. Groups were retained here a) because effects are unchanged and b) because removing groups would render them discoverable by their absence. Group composition is available to those with HCP Restricted Access, as a Subject Key.
