# Supplementary figures and images for: Prevalent and sex-biased breathing patterns modify functional connectivity MRI in young adults
Source: Nat Commun. 2020 Oct 20;11:5290. doi: 10.1038/s41467-020-18974-9 (PMC7576607; doi:10.1038/s41467-020-18974-9)

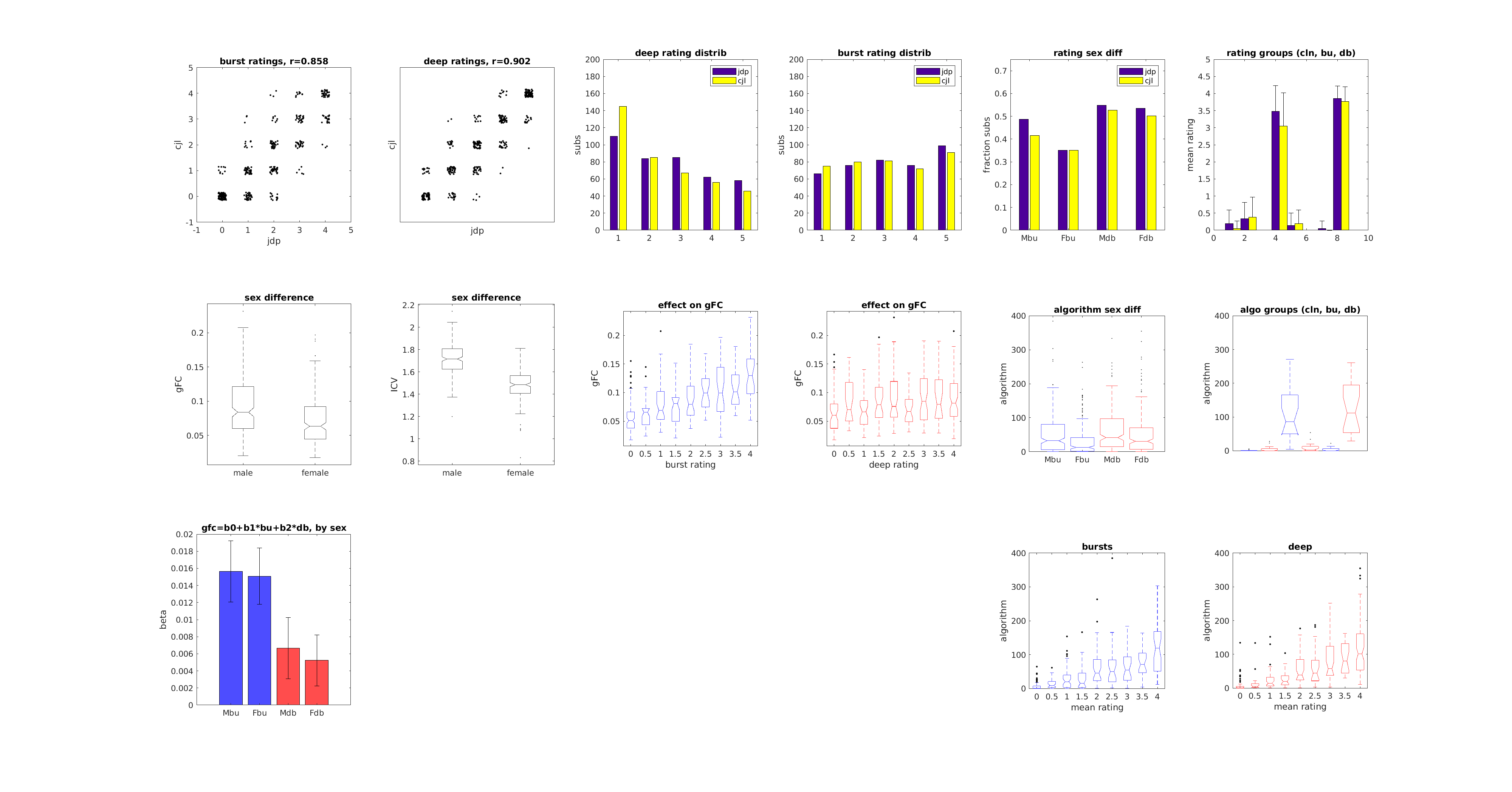

Supplement: Supplementary file 10 — Source Data [file 41467_2020_18974_MOESM10_ESM.zip › Figure4/fig4simple.png]
